# Comparative proteomics reveals that YK51, a 4-Hydroxypandurantin-A analogue, downregulates the expression of proteins associated with dengue virus infection

**DOI:** 10.7717/peerj.3939

**Published:** 2018-01-30

**Authors:** Wei-Lian Tan, Yean Kee Lee, Yen Fong Ho, Rohana Yusof, Noorsaadah Abdul Rahman, Saiful Anuar Karsani

**Affiliations:** 1 Institute of Biological Sciences, Faculty of Science, University of Malaya, Kuala Lumpur, Malaysia; 2 Drug Design and Development Research Group (DDDRG), University of Malaya, Kuala Lumpur, Malaysia; 3 Department of Chemistry, Faculty of Science, University of Malaya, Kuala Lumpur, Malaysia; 4 Department of Molecular Medicine, Faculty of Medicine, University of Malaya, Kuala Lumpur, Malaysia; 5 University of Malaya Centre for Proteomics Research (UMCPR), Medical Biotechnology Laboratory, University of Malaya, Kuala Lumpur, Malaysia

**Keywords:** Dengue virus type-2, Proteomics, Anti-viral compound, Inhibitory activity

## Abstract

Dengue is endemic throughout tropical and subtropical regions of the world. Currently, there is no clinically approved therapeutic drug available for this acute viral infection. Although the first dengue vaccine Dengvaxia has been approved for use in certain countries, it is limited to those without a previous dengue infection while the safety and efficacy of the vaccine in those elderly and younger children still need to be identified. Therefore, it is becoming increasingly important to develop therapeutics/drugs to combat dengue virus (DENV) infection. YK51 is a synthetic analogue of 4-Hydroxypandurantin A (a compound found in the crude extract of the rhizomes of *Boesenbergia rotunda*) that has been extensively studied by our research group. It has been shown to possess outstanding antiviral activity due to its inhibitory activity against NS2B/NS3 DENV2 protease. However, it is not known how YK51 affects the proteome of DENV infected cells. Therefore, we performed a comparative proteomics analysis to identify changes in protein expression in DENV infected HepG2 cells treated with YK51. Classical two-dimensional gel electrophoresis followed by protein identification using tandem mass spectrometry was employed in this study. Thirty proteins were found to be down-regulated with YK51 treatment. *In silico* analysis predicted that the down-regulation of eight of these proteins may inhibit viral infection. Our results suggested that apart from inhibiting the NS2B/NS3 DENV2 protease, YK51 may also be causing the down-regulation of a number of proteins that may be responsible in, and/or essential to virus infection. However, functional characterization of these proteins will be necessary before we can conclusively determine their roles in DENV infection.

## Introduction

Dengue is an acute mosquito-borne viral infectious disease. It is caused by DENV that is transmitted by female *Aedes aegypti* and *Aedes albopictus* mosquitoes. Dengue is endemic throughout tropical and subtropical regions of the world with more than 125 dengue-endemic countries and 3.6 billion individuals at risk of infection. The disease is widespread due to several factors such as the modern dynamics of climate change, globalization, travel, trade, socioeconomics, settlement and viral evolution (Murray, Quam & Wilder-Smith, 2013). The most effective and long-term solution against dengue will be vaccination. Currently, there is no clinically approved therapeutic drug available for this acute viral infection (Wahab, Yusof & Rahman, 2007). Although the first dengue vaccine Dengvaxia has been approved for use in certain countries, it is limited to those without a previous dengue infection and the safety and efficacy of the vaccine in those elderly and younger children still needs to be identified. Thus, there is a dire need for the development of therapeutics against dengue virus infection.

The viral encoded protease NS2B/NS3 is pivotal for viral replication and virus assembly. The NS3serine protease together with the cofactor NS2B functions to cleave the nonstructural region of the viral polyprotein at the NS2A/NS2B, NS2B/NS3, NS3/NA4A and NS4B/NS5 junctions. Therefore, NS2B/NS3 represents an attractive target for drug design against viral replication. Such targeted drug may reduce the viral load at an early stage of the mild disease form and prevent patients from developing acute severe infection. A promising source for anti-DENV agents are natural or synthetic non-peptide inhibitors (Li et al., 2014). Compounds from the roots of the plant *Boesenbergia rotunda* has been explored and screened for its inhibitory activity against the DENV protease (Kiat et al., 2006). One compound, 4-hydroxypanduratin A, was found to be particularly potent. This compound competitively inhibited the activity of the DENV2 serine protease with an inhibition constant (Ki) of 25 µM. Therefore, the molecular structure of 4-hydroxypanduratin A was used as a model for designing potentially more potent drug analogs.

**Figure 1:**
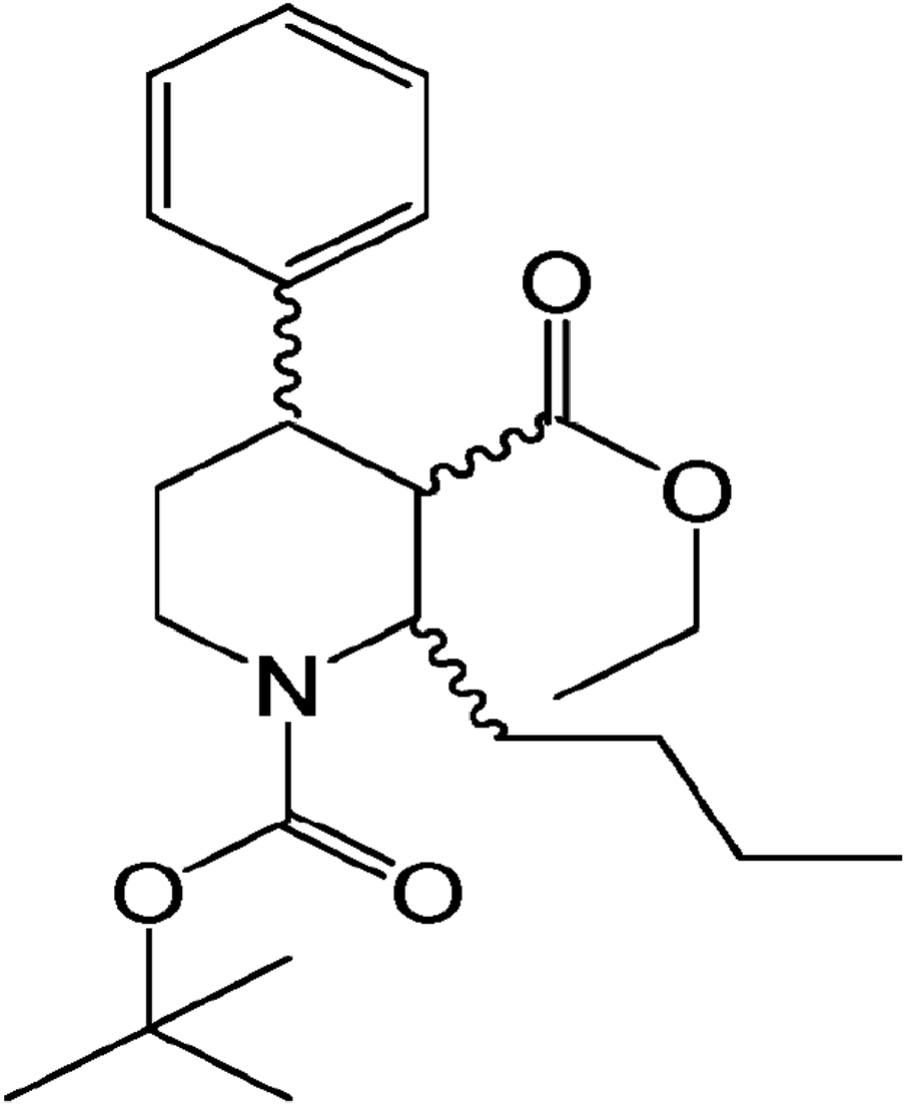
Chemical structure of YK51. Its chemical name is ethyl (1-tert-butoxycarbonyl-2-butyl-4-phenyl)-piperidinyl-3-carboxylate.

Based on the structure of 4-hydroxypanduratin A, several analogs of this molecule have been synthesized, which involved the synthetic strategies of dihydropyridine synthesis, 1,4-Michael addition and Weinreb ketone synthesis by using nicotinic acid as the starting material (Gan et al., 2017; Muhamad et al., 2010b). The efficacy and inhibitory effects of five of these molecules (YK38, YK51, YK73, YK73X, and YK101) have been determined in an **in vitro** model where the compound YK51 was found to completely prevent DENV2 infection in HepG2 cells as indicated by the absence of any cytopathic effect (Muhamad et al., 2010a). YK51 chemical structure is illustrated in Fig. 1. The results strongly suggested the potential of YK51 as an antiviral agent for dengue. However, the molecular mechanism of the cytopathic/antiviral/inhibitor action of YK51 remains unknown.

Different anti-viral compounds may affect different molecular pathways within host cells during virus infection. For instance, interleukin-4 treatment has been shown to interrupt HepG2 cells transcription and proteolysis during Hepatitis B virus (HBV) infection whereas interferon alpha treatment on HBV infected HepG2 cells influenced cytoskeletal matrix,heat shock stress, kinases/signal transduction, and protease/proteasome components of host cells (Wang et al., 2009; Yao et al., 2011). HepG2 cells which are human liver origin have always been used as a study model for dengue infection because human liver is one of the primary organs to present symptomatic dengue disease for clinical diagnosis (Jessie et al., 2004; Thepparit et al., 2013). Nevertheless, several studies using hepatocyte cell line have been successfully demonstrating the anti-DENV properties of the selected drugs like AR-12 which has known inhibitory effects on DENV-associated host protein, GRP78 expression and HS-72, inhibitor of inducible heat shock protein 70 (Hsp70i). Hsp70i is a DENV host factor and novel drug target that has been discovered using proteomic approach (Chen et al., 2017; Howe et al., 2016). Hence, understanding the effects of YK51 compound at the protein expression level in virus infected host cells may lead to the design of more effective drugs. As the anti-DENV effect of YK51 has been substantiated in previous study, a robust top-down approach will be employed to elucidate all YK51-inhibited DENV host factors and other YK51-associated host proteins that against DENV post-infection. Therefore, we have performed a comparative proteomics analysis to determine the effects of YK51 compound at the protein expression profile in DENV infected HepG2 cells to gain an insight on its mode of action.

## Materials and Methods

### Cell lines

HepG2 human hepatic cells (HB-8065, ATCC, USA), African green monkey kidney (Vero) cells (CCL-81, ATCC, USA) and *Aedes albopictus* clone C6/36 mosquito cells (CRL-1660, ATCC, Manassas, VA, USA) were used in this study. HepG2 and Vero cells were grown in DMEM medium (Gibco, Carlsbad, CA, USA) supplemented with 10% heat-inactivated fetal bovine serum (FBS) (Gibco, Carlsbad, CA, USA) at 37 °C in a humidified incubator with 5% carbon dioxide. C6/36 cells were cultivated in L-15 medium (Sigma-Aldrich^®^, St. Louis, MO, USA) supplemented with 10% FBS and 10% tryptose phosphate broth (Sigma-Aldrich^®^, St. Louis, MO, USA) at 28 °C in a non-humidified environment.

### Virus stock propagation and titration

DENV2 New Guinea C strain was propagated in a monolayer of C6/36 cells when the cells are 80% confluent. The cell monolayer was inoculated with DENV2 at a multiplicity of infection (MOI) of 1.0 and incubated at 28 °C until cytopathic effect (CPE) were observed (seven to 10 days). MOI is the average number of virus particles infecting each cell. It is calculated by dividing the total number of infectious virus particle (ffu) used with the number of cells being infected. The inoculated cells were then lysed by freeze-thawing. Virus stocks were harvested from the culture supernatant and stored at −80 °C . Mock control cells were cultured in parallel with infected cells but without virus infection and processed in the same manner. Virus titer was determined by fluorescent focus assay (FFA) on Vero cells and expressed as fluorescent forming units (FFU)/ml (Payne et al., 2006).

### Virus inhibitor

YK51 compound was dissolved in methanol to a concentration of 1 mg/ml and filtered with 0.2 µm pore size sterile syringe filter (Sartorius, Göttingen, Germany) before use.

### Flow cytometry analysis

The percentage of viral infection in HepG2 cells at various MOI values and incubation times was quantified by flow cytometry analysis as previously described (Thio et al., 2013). Briefly, HepG2 cells were seeded overnight at a density of 2 × 10^6^ cells per 25 cm^2^ culture flask and then inoculated with DENV2 at MOI values of 5, 10 and 15 respectively. The viral inoculum was removed after 2 h of infection and the cells were further in for incubated DMEM maintenance medium (2% FBS) for 24 and 48 h. Control cells were incubated with culture supernatant of mock control C6/36 cells and processed in the same manner. The cells were harvested after incubation at their designated time-points. Subsequently, 1.5 × 10^6^ cells from each sample were fixed with 3.7% formaldehyde in phosphate buffered saline (PBS) (Sigma-Aldrich, St. Louis, MO, USA) for 30 min. The fixed cells were washed twice with staining buffer (0.1% sodium azide and 1% FBS in PBS) and incubated with dengue virus type 2 monoclonal antibodies (Novus Biologicals, San Diego, CA, USA) diluted 1:50 in staining buffer at 37 °C for 60 min. The cells were then washed thrice with staining buffer and further incubated with goat anti-mouse IgG secondary antibody conjugated with FITC (Novus Biologicals, San Diego, CA, USA) diluted 1:50 in staining buffer for 30 min at 37 °C in the dark. After extensive washing, the stained cells were re-suspended in PBS and analyzed with FACSCanto II flow cytometer (BD Biosciences, San Jose, CA, USA) using FACSDiva v6.1 software.

### ApoTox-Glo^®^ Triplex assay

Cell viability, cytotoxicity, and apoptosis of infected HepG2 cells in response to different concentrations of YK51 compound were analyzed using ApoTox-Glo™ Triplex Assay kit (Promega, Madison, WI, USA) following the manufacturer’s instructions. Briefly, HepG2 cells were seeded overnight in 96-well optical bottom plate (Nunc™, Rochester, NY, USA) at a density of 1 × 10^4^ cells per well and then inoculated with DENV2 at the optimized MOI of 15. The viral inoculum was removed after 2 h of infection and the cells were further incubated in DMEM maintenance medium (2% FBS) containing 2, 5, and 10 µg/ml of YK51 compound respectively for 24 and 48 h at 37 °C . Control cells were cultured in parallel and processed in the same manner. Thereafter, viability/cytotoxicity reagent was added to each well of 96-well plate and incubated at 37 °C for 30–180 min. Fluorescence was measured at 400 nm excitation/505 nm emission for viability and 485 nm excitation/520 nm emission for cytotoxicity using Infinite^®^ M1000 PRO multiplate reader (Tecan, Männedorf, Switzerland). Caspase-Glo 3/7 reagent was then added to each well and incubated at room temperature for 30–180 min. Luminescence was measured at 1.0 s using luminescence protocol. Cell viability and apoptosis of DENV2-infected HepG2 cells at various MOI values and time points were also evaluated using the same assay.

### Protein sample preparation

HepG2 cells were seeded overnight at a density of 1 × 10^6^ cells per 25 cm^2^ culture flasks and then inoculated with DENV2 at the optimized MOI of 15. The viral inoculum was removed after 2 h of infection and the cells were further incubated at the optimized time-point (24 h) in DMEM maintenance medium containing the optimized concentration of 5 µg/ml YK51 compound. Samples with different conditions were also cultured accordingly and processed in the same manner. Subsequently, the cells were harvested for protein extraction. The whole cell proteome was extracted in lysis buffer (7 M urea, 2 M thiourea, 4% CHAPS, 2% IPG buffer, 40 mM DTT) on ice for 30 min. The extracted proteins were centrifuged at 13,000 rpm for 5 min to remove cellular debris. The resulting supernatant was cleaned using 2-D clean-up kit (GE Heathcare, Danderyd, Sweden) following the manufacturer’s instructions. Quick Start™ Bradford protein assay kit 1 (Bio-Rad Laboratories, Hercules, CA, USA) was then used to quantify the protein concentration.

### Two-dimensional gel electrophoresis (2-DGE)

Samples containing 40 µg (analytical gel) and 80 µg of protein (preparative gel) were mixed with rehydration solution (7 M urea, 2 M thiourea, 2% CHAPS, 0.5% IPG buffer, 0.002% bromophenol blue and DTT) to a final volume of 250 µl per DryStrip. Protein samples were loaded onto 13 cm linear Immobilized pH gradient strips (IPG, GE Healthcare, Danderyd, Sweden) pH 3–10 by in-gel rehydration for 18 h at room temperature using Immobiline™ DryStrip re-swelling tray (GE Healthcare, Danderyd, Sweden). The first-dimension separation was performed at 20 °C using Ettan IPGphor II IEF System (GE Healthcare, Danderyd, Sweden) under the following conditions: cycle 1 (step-and-hold, 550 Vh, 500 V); cycle 2 (gradient, 1,000 Vh, 1,000 V); cycle 3 (gradient, 11,000 Vh, 8,000 V), and cycle 4 (step-and-hold, 7,000 Vh, 8,000 V). The IPG strips were then reduced with SDS equilibration buffer (6 M urea, 75 mM Tris-HCl at pH 8.8, 29.3% glycerol, 2% SDS and 0.002% bromophenol blue) containing 1% DTT (Gold Biotechnology^®^, St. Louis, MO, USA) for 15 min and alkylated in SDS equilibration buffer containing 2.5% Iodoacetaminde (Merck, Darmstadt, Germany) for a further 15 min. The second-dimensional separation was carried out using SE 600 Ruby electrophoresis system (GE Healthcare, Danderyd, Sweden). Proteins were resolved on 12.5% SDS-PAGE homogenous gels under the following conditions: 40 mA/gel, 100 V, 50 W for 30 min followed by 40 mA/gel, 100 V, 500 W for approximately 100 min (until the tracking dye reached the end of the gel).

### Gel visualization: silver staining

Upon completion of 2-DGE, gels were stained with an MS-compatible silver staining protocol (Yan et al., 2000). Briefly, the gels were fixed (10% acetic acid and 40% ethanol) for at least 30 min followed by sensitization (0.3% ethanol, 25% glutaraldehyde, 12.65mM sodium thiosulfate and 0.83 M sodium acetate) for 30 min. The gels were washed thrice with deionized water for 5 min and stained with silver nitrate solution (14.72 mM silver nitrate and 0.015% formaldehyde) for 20 min. The gels were then washed twice with deionized water for 1 min and developed with developing solution (0.24 M sodium carbonate and 0.015% formaldehyde) for 4 min. The gels were placed in stopping solution (0.05 M EDTA disodium salt) for 10 min followed by washing with deionized water for 10 min. Glutaraldehyde and 37% formaldehyde was omitted from the sensitizing and staining steps when staining preparative gels.

### Gel image analysis

Silver stained gels were scanned using Image Scanner™ III (GE Healthcare, Danderyd, Sweden) and analyzed using Progenesis Same Spot v2.0 software (Nonlinear Dynamics, USA) for gel alignment, spot detection and statistical analysis. The comparison was performed between two experiment groups. The proteome profile of DENV2-infected HepG2 cells were compared with the protein profile of infected HepG2 cells treated with YK51 compound. On the other hand, the protein profile of mock control cells was compared with the protein profile of YK51 compound treated HepG2 cells. Each experiment group consisted of five biological replicates (*n* = 5). Protein spots that can be observed in all 5 biological replicates and changed >1.5 folds with an ANOVA *p*-value <0.05 were considered have significantly different protein expression. The selected protein spots were then excised from ten preparative gels for protein identification.

### In-gel trypsin digestion

In-gel digestion was performed using MS grade Trypsin Gold (Promega, Madison, WI, USA) [11]. The silver stained gel plugs were destained twice with destaining solution (15 mM potassium ferricyanate in 50 mM sodium thiosulphate) for 10 min and washed twice with deionized water for 2 min. The destained gel plugs were reduced with reducing solution (10 mM DTT in 100 mM ammonium bicarbonate) at 60 °C for 30 min and allowed to cool to room temperature for 5 min. The reduced gel plugs were then alkylated with alkylating solution (55 mM IAA in 100 mM ammonium bicarbonate) in the dark for 20 min and washed thrice with washing solution (50 mM ammonium bicarbonate in 50% acetonitrile) for 20 min. The alkylated gel plugs were dehydrated with 100% acetonitrile (Merck, Darmstadt, Germany) for 15 min and the opaque white gel plugs were dried in a vacuum concentrator for 15 min. The plugs were then digested with 25 µl of 6 ng/µl trypsin gold in 50 mM ammonium bicarbonate at 37 °C for 16 h in a water bath. After incubation, the first extraction was carried out with 50% acetonitrile for 15 min followed by second extraction with 50 µl of 100% acetonitrile for 15 min. The extracted peptides were pooled into the same tube and dried using a vacuum concentrator.

### Mass spectrometry (MS)

The dried peptides were reconstituted in 10 µl of 0.1% formic acid and desalted using ZipTip C18 (Millipore, USA) as described by the manufacturer. The eluted peptides were dried using a vacuum concentrator. Liquid chromatography-mass spectrometric analysis (LC/MS) was then performed using Agilent 1200 HPLC-Chip/MS Interface coupled with Agilent 6520 Accurate Mass Q-TOF LC/MS instrument (Agilent Technologies, Santa Clara, CA, USA). The dried peptides were reconstituted with 5 µl of 0.1% formic acid and loaded onto a large capillary chip, C18, enrichment column with a volume of 160 nl for sample enrichment followed by separation on an analytical column with a 75 µm inner diameter and 150 mm length (Agilent part no: G4240-62010). Both capillary and Nano pumps used binary solvents. The eluents A and B were 0.1% formic acid in water and 0.1% formic acid in 90% acetonitrile respectively. A 4 µl/min flow rate of solvent A was used for sample loading with 2 µl injection volume. In contrast, 47 min gradient delivered by Nano pump with a flow rate of 0.3 µl/min for solvent B was used for separation using the following gradient: Initial 3% B, 30 min 50% B, 32 min 95% B and 39 min 95% B. Thereafter, Agilent ESI Q-TOF (Agilent Technologies, Santa Clara, CA, USA) was used for MS analysis. Electrospray ionization source was operated in positive ion mode with Vcap and fragmentor voltage of 1,900 V and 175 V respectively. Gas temperature was set at 325 °C and drying gas flow was set at 5.0 L/min. The slope collision energy was 3.7 V/ (100 Da) offset 2.5 V. Spectra were acquired in MS/MS mode with an MS scan range of 110–3,000 m/z and MS/MS scan range of 50–3,000 m/z. The acquisition rate for MS and MS/MS was 8 and 4 spectra/sec and acquisition time was 125 and 250 ms/spectrum respectively. The double, triple and above triple charged ions are included in the precursor charge state selection and preference.

The data obtained from LC-MS/MS was analyzed using Agilent Spectrum Mill MS Proteomics Workbench software (version Rev A.03.03.084 SR4) and searched against the NCBI (National Center for Biotechnology Information) *Homosapien* and *Flavivirus* protein databases. The mass range of precursor ions was set from 600 to 4,000 Da. Fixed modification of Carbamidomethylation (c) was selected for protein identification and characterization. The program filtered results by overall protein score more than 11 and individual peptide score greater than 6 with % SPI greater than 60. Additional autovalidation was then performed to filter proteins by score of 20 based on the default protein rules.

### Pathway analysis

Ingenuity Pathways Analysis (IPA) software (Ingenuity^®^ Systems, Redwood City, CA, USA; http://www.ingenuity.com) was used to identify relationships, mechanisms, functions, and related pathways affected by the changes in expressed proteins. A data set containing gene the identifiers (details of the proteins) and their corresponding expression values were uploaded to the IPA software. The filter parameters and other core setting options were set before the analysis. Cutoff values at least 1.5 fold change difference and *p*-values less than 0.05 was selected. Direct and indirect relationships of proteins were restricted to human species only in IPA analysis. Each identifier was mapped to its corresponding object and overlaid onto a global molecular network established from information contained in the Ingenuity Knowledge Base. The related biological networks were generated algorithmically and functional and canonical pathway analysis was performed using the association of the proteins with the IPA Knowledge Base. The significance of the associations was assessed with Fisher’s exact test.

### Quantitative Real-Time PCR

Total RNA of DENV2-infected HepG2 cells treated and untreated with YK51 compound from three biological replicates were extracted using Qiagen RNeasy Mini Kit (Qiagen, Valencia, CA, USA) following the manufacturer’s instruction. Genomic DNA contamination was eliminated from the extracted RNA using Deoxyribonuclease I treatment (Invitrogen, USA). The purified RNA was qualitatively assessed and quantified using GeneQuant™ 1300 spectrophotometer (GE Healthcare, Danderyd, Sweden). The RNA concentration was measured at absorbance 260 nm (A260) and RNA purity was determined at absorbance ratio A260/A280 and A230/A260. The integrity and size distribution of total RNA was confirmed by visualization of distinct 18S and 28S ribosomal RNA bands resolved on 1% agarose gel. RNA was then converted to cDNA using a High Capacity RNA-to-cDNA Kit (Applied Biosystems, USA) as described by the manufacturer. The cDNA template (100 ng) was mixed with a probe specific for the gene of interest, TaqMan Gene Expression Assay and TaqMan Fast Advance Master Mix (Applied Biosystems™, USA). This was followed by real-time PCR quantification using StepOne Plus™ Real-Time PCR System (Applied Biosystems, Foster City, CA, USA) and analyzed using StepOne Software v2.2 (Applied Biosystems, Foster City, CA, USA). The expression level of each targeted gene was normalized against the expression of an endogenous control gene, glyceraldehyde-3-phosphate dehydrogenase (GAPDH). Differences in gene expression, expressed as fold-change, were calculated based on the delta-delta Ct (ΔΔCt) algorithm, 2^(−ΔΔCt)^ method. Statistical significance of altered gene expression was determined using Student’s *t*-test where a *p* < 0.05 was considered significant (Chang et al., 2013).

**Figure 2:**
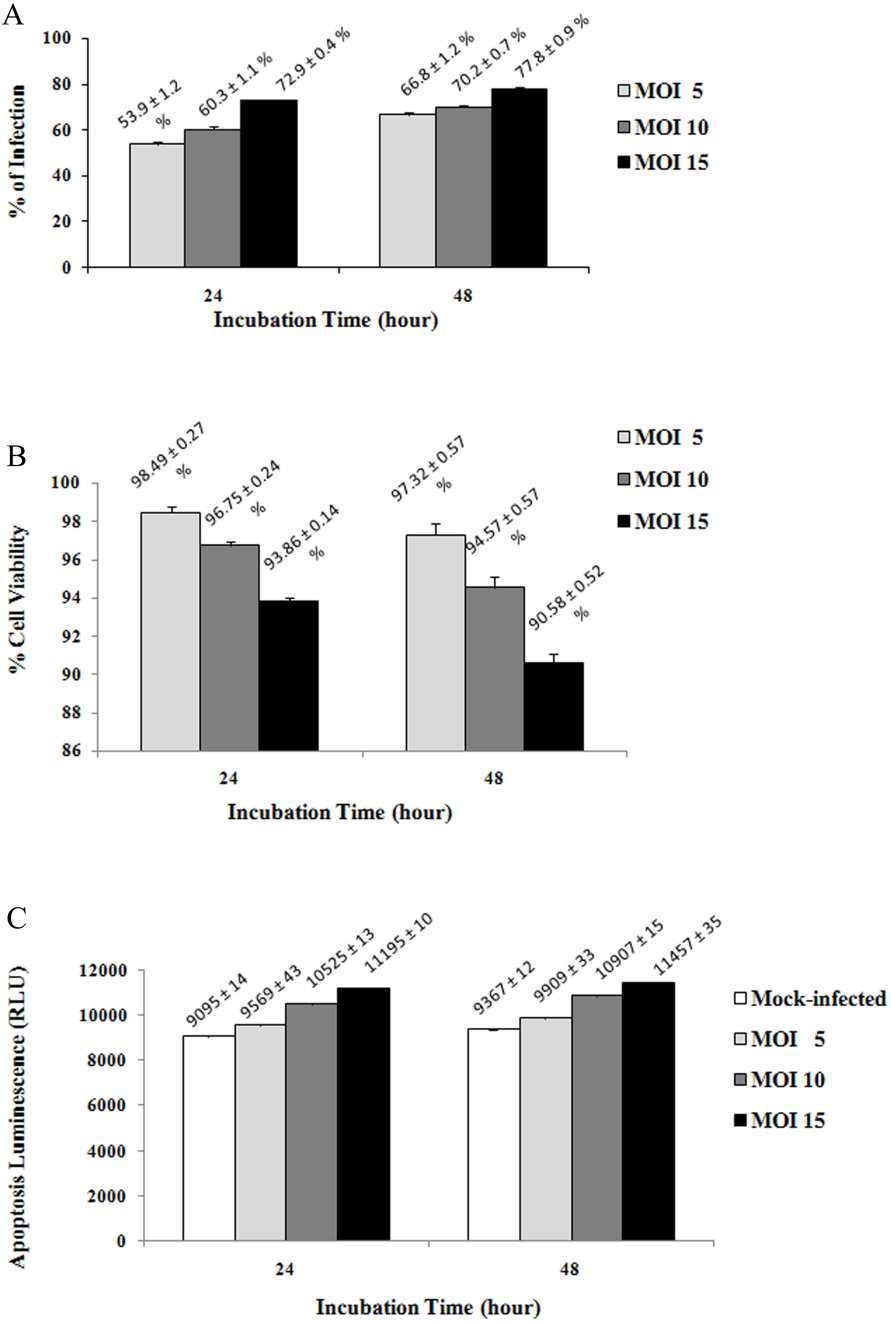
Percentage of viral infection (A), cell viability (B) and apoptosis (C) of infected HepG2 cells at different MOI values and incubation times. The highest percentage of infection was observed at MOI of 15 at both 24 (72.93% ± 0.38) and 48 h (77.76% ± 0.90) post infection with respect to the uninfected control (100%). No significant reduction in the percentage of cell viability was observed and a low level of apoptosis event was detected during the early stages of infection. However, the percentage of viable cells at 48 h post infection was found to be slightly lower than the cell viability at 24 h post infection. The caspase-3/7 activity of HepG2 cells at 48 h post infection was also greater than the apoptosis events at 24 h post infection. Hence, the percentage of infection, cell viability and apoptosis of infected HepG2 cells were observed to vary in a MOI and time-dependent manner. The uninfected cells were normalized with infected cells in 2(A) and (2b). Results are expressed as the mean ± standard deviation of three independent experiments.

## Results

### Quantitative analysis of viral infection, cell viability and apoptosis in infected HepG2 cells

Prior to 2-DGEanalysis, optimization of the MOI and incubation time were performed to identify the condition that defined as early infection, where is the stage before the incipient cytopathic effect or cell death. This is to ensure that response of the whole cell proteome is caused by early-infection only, but not due to cytopatic effect and apoptosis event. The percentage of DENV2 infection in HepG2 cells at MOI of 5, 10, and 15 at 24 and 48 h post infection was determined by cytoplasmic staining with anti-dengue monoclonal antibody followed by FITC-conjugated secondary antibody. The different staining intensity and number of infected cells were quantified using flow cytometry for the selection of optimal MOI and incubation time. The results (Fig. 2A) showed that higher MOI values and longer incubation times resulted in a higher percentage of infected cells. The highest percentage of infection was observed at MOI of 15 at both 24 (72.93% ± 0.38) and 48 h (77.76% ± 0.90) post infection.

The early effects of DENV2 infection on cell viability and apoptosis of HepG2 cells were analyzed using ApoTox-Glo™ Triplex Assay. The difference in the percentage of cell viability at the various MOI values was not significant at 24 h post infection (Fig. 2B) where only <7% cell death was observed. At 48 h post infection, cell death increased to a maximum of 10%. Virus-induced apoptosis event in cells was examined by measuring caspase-3/7 activity. DENV2 induced a low level of apoptosis in HepG2 cells during the early stages of infection (Fig. 2C). The relative luminescent units (RLU) were found to increase when the MOI and incubation time were increased. The caspase-3/7 activities of HepG2 cells at various MOI values at 48 h post infection were higher than at 24 h post infection. Based on these results, MOI 15 and 24 h incubation time were selected as the optimal conditions for viral infection as it provided a high percentage of infection with low virus-induced cytopathology and minimal percentage of cell death and apoptosis when compared to the mock control.

**Figure 3:**
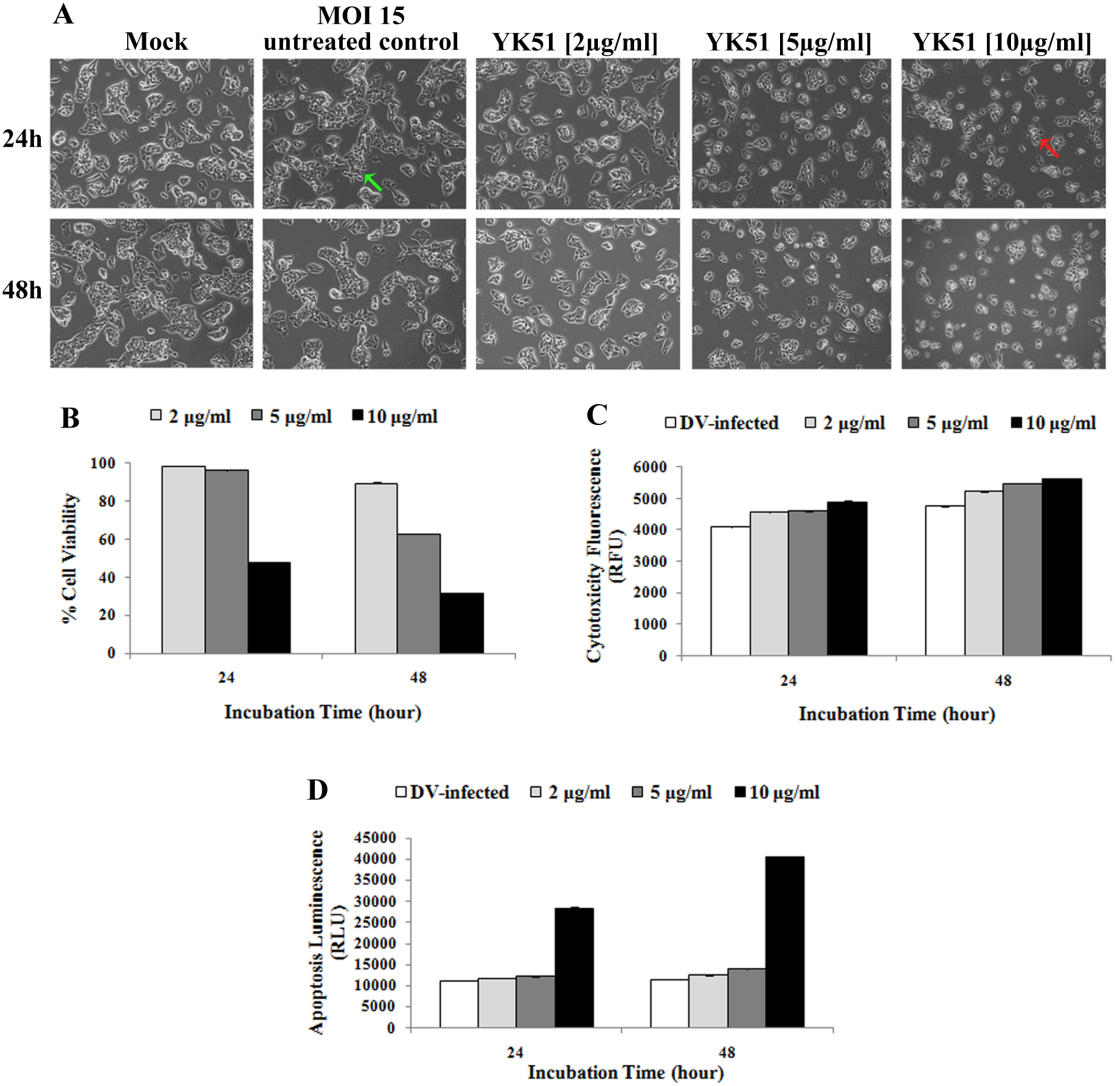
Cell morphology (A), viability (B), cytotoxicity (C) and apoptosis (D) of infected HepG2 cells at different compound concentrations and incubation times. Cell shrinkage and rounded morphology (red arrow) were observed in HepG2 cells with increasing concentrations of YK51 compound. Reduction of cell populations was also detected during the course of prolonged incubation with the compound. Uninfected and untreated HepG2 cells served as the negative control. Green arrow indicated the normal infected HepG2 cells. All images were captured at 100× magnification. High compound dose and prolonged exposure period significantly decreased the percentage of cell viability to more than 50% and markedly increased the cytotoxicity and caspase-3/7 activities (>2-fold) when compared to the untreated control. Thus, YK51 compound reduced the cell viability while increased the cytotoxicity and apoptosis rates in a dose- and time-dependent manner. The results are presented as the mean ± standard deviation of three independent experiments.

### Determination of optimal conditions for drug treatment

The concentration and exposure time of YK51 need to be determined in order to avoid any cytotoxic effects on host cells and ensure host cells proteome reflected the true response on the effect of YK51. Optimal YK51 concentration to study the early effects of treatment was determined by examining the morphology of YK51-treated HepG2 cells and by using the ApoTox-Glo™ Triplex Assay. Morphological alterations induced by different compound dosage and exposure times were assessed under an inverted microscope at 100× magnification. Cell shrinkage, rounding and detachment were observed in HepG2 cells with increasing concentrations of YK51. Destruction of cell monolayer and reduction of cell populations were also detected during the course of prolonged incubation with the synthetic compound which was not seen in untreated HepG2 cells (Fig. 3A).

ApoTox-Glo™ Triplex Assay was then performed to assess cell viability, cytotoxicity and apoptosis of infected HepG2 cells upon drug treatment. The infected HepG2 cells were incubated with YK51 compound at 2, 5 and 10 µg/ml for 24 and 48 h. DENV2-infected HepG2 cells without YK51 treatment served as the negative control. Methanol is used as a solvent for YK51 compound in previous study (Muhamad et al., 2010a). The maximum non-toxic dose of methanol in HepG2 cells has been determined to be 6% (v/v). Control cultures treated with 6% methanol did not exhibit any adverse effect compared to untreated controls. Therefore, YK51 compound was also dissolved in methanol in this study and we only added 0.1% methanol to the cells. After 24 h exposure to YK51 (at 2 and 5 µg/ml), cell viability was not significantly affected when normalized with control cells. However, a reduction of >50% in cell viability was observed at 10 µg/ml of YK51. Increasing the duration of exposure to 48 h also resulted in a marked decrease of cell viability (Fig. 3B).

Results of the cytotoxic assay following exposure to YK51 compound showed that there was no appreciable difference in the relative fluorescent units (RFU) after 24 h exposure to varying YK51 concentrations compared to untreated control (Fig. 3C). This suggested that YK51 had negligible toxicity in HepG2 cells under these conditions. YK51 has been previously shown to only show cytotoxic effect in HepG2 cells at 40 µg/ml (Muhamad et al., 2010a). In addition, apoptosis assay revealed that exposure to YK51 at 10 µg/ml significantly increased the number of apoptotic HepG2 cells where a two-fold increase in RLU was observed. No significant increase was observed at <10 µg/ml (Fig. 3D).

Taken together, these results suggested that YK51 treatment lead to a dose- and time-dependent decrease in the percentage of viable cells and an increase in cytotoxicity and caspase-3/7 activity consistent with the apoptotic process. However, these effects were only significant at a high dosage of YK51. Therefore, for further experiments, YK51 concentration of 5 µg/ml and 24 h incubation time was selected as the optimal dose and exposure time as it was the highest concentration and time point that caused minimal cytotoxic effect and cell death. Under the optimal condition, no abnormal morphological changes should observe on HepG2 cells. This indicated that HepG2 cells are remaining healthy at early stage of viral infection and drug treatment.

### Comparative proteomics analysis: DENV2-infected HepG2 (DENV2 + YK51) versus DENV2-infected HepG2 cells (DENV2-YK51)

**Figure 4:**
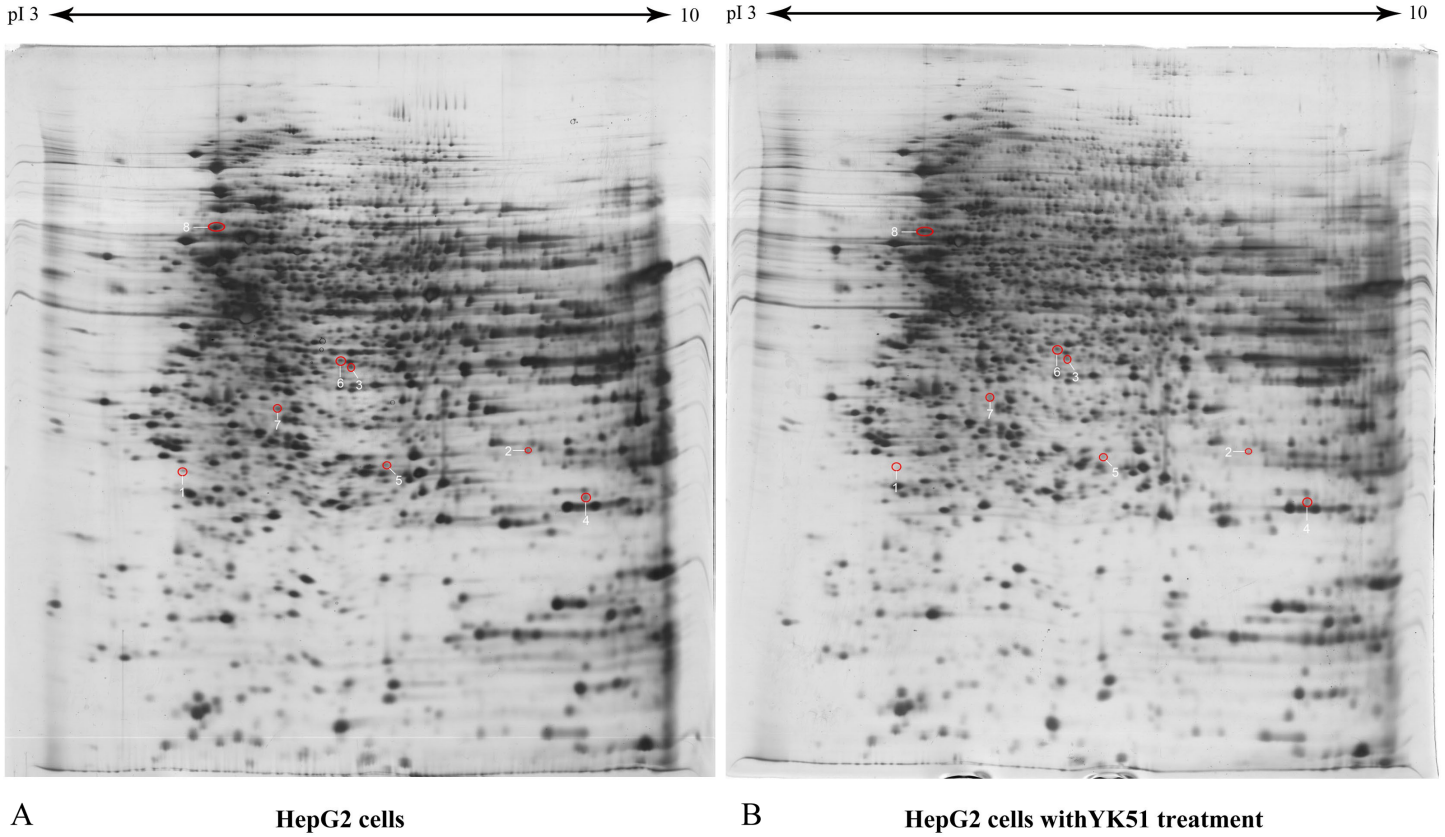
The proteome maps of differentially expressed whole cell proteins in control and YK51-treated HepG2 cells. HepG2 proteomes (40 µg) were profiled using 13 cm, pH 3 to 10 linear IPG strip and 12.5% SDS gel. There were eight identified protein spots in the gel map. Red circles denote identified protein spots which changed in abundance compared to proteome of HepG2 cells without YK51 treatment. The spot numbers correspond to spot numbers in Table 1.

HepG2 cells were subjected to four different experiments: mock control (untreated), YK51-treated, DENV2-infected HepG2 cells without YK51 treatment (DENV2-YK51), and DENV2-infected HepG2 cells with YK51 treatment (DENV2 + YK51). To determine the effects of YK51 treatment on uninfected cells, the proteome profiles of mock control and YK51-treated HepG2 cells were compared. The comparison was performed between two experiment groups. Each experiment group consisted of five biological replicates (*n* = 5). Our results showed that treatment with YK51 compound did not cause significant changes in the proteome profile of HepG2 cells. There are only eight proteins were found altered in abundance (≥1.5 folds with an ANOVA *p*-value ≤ 0.05), which depicted in Table 1. Figure 4 showed the proteome maps of differentially expressed whole cell proteins in control and YK51-treated HepG2 cells.

**Table 1:** List of differentially expressed proteins in the HepG2 cells treated with YK51 compound.

Spot ID	Protein name (Gene symbols)	Swiss-Prot accession	Log normalized volume ± SD^a^		Fold change^b^	Student’s *t*-test	Distinct sum MS/MS score^c^	% Seq/Matched peptides^d^	MW (kDa)/pI^e^
			Uninfected, Untreated (*n* = 5)	Uninfected, Treated (*n* = 5)					
1	T-complex protein 1 subunit theta (CCT8)	P50990	6.12 ± 0.20	5.56 ± 0.59	−2.2	0.027	51.28	8/4	60.4/5.5
2	Sepiapterin reductase (SPR)	P35270	6.51 ± 0.14	6.20 ± 0.30	−1.9	0.021	43.36	12/3	31.3/8.5
3	Protein phosphatase 1, catalytic subunit, alphaisoform (PPP1CA)	P62136	6.66 ± 0.07	6.41 ± 0.22	−1.7	0.014	21.05	5/2	45.8/7.2
4	Deoxythymidylate kinase (DTYMK)	P23919	6.49 ± 0.08	6.33 ± 0.08	−1.5	0.004	1114.62	24/7	28.6/6.7
5	Platelet-activating factor acetylhydrolase IB subunit gamma (PAFAH1B3)	Q15102	6.43 ± 0.07	6.24 ± 0.10	−1.5	0.002	38.53	11/3	25.7/6.3
6	COP9 signalosome complex subunit 5 (COPS5)	Q92905	6.59 ± 0.04	6.40 ± 0.17	−1.5	0.013	44.20	6/3	42.6/9.4
7	Annexin IV (ANXA4)	P09525	6.42 ± 0.04	6.22 ± 0.18	−1.5	0.013	29.90	9/3	36.1/5.8
8	Serine/threonine-protein phosphatase 2A 65 kDa regulatory subunit A alpha isoform (PPP2R1A)	P30153	6.98 ± 0.10	6.80 ± 0.21	−1.5	0.048	267.22	32/19	65.3/5.0

**Notes.**

a Logarithm of normalized spot volume with standard deviation.

b Negative sign (−) of fold change signifies decreased abundance of identified proteins compared to HepG2 cells without YK51 treatment.

c Sum of the *z*-scores from each distinct detected peptide in a same protein species.

d Percentage of protein sequence coverage (% Seq) and number of distinct detected peptides.

e Molecular weight of protein in kilo Dalton and isoelectric focusing point of a protein.

**Figure 5:**
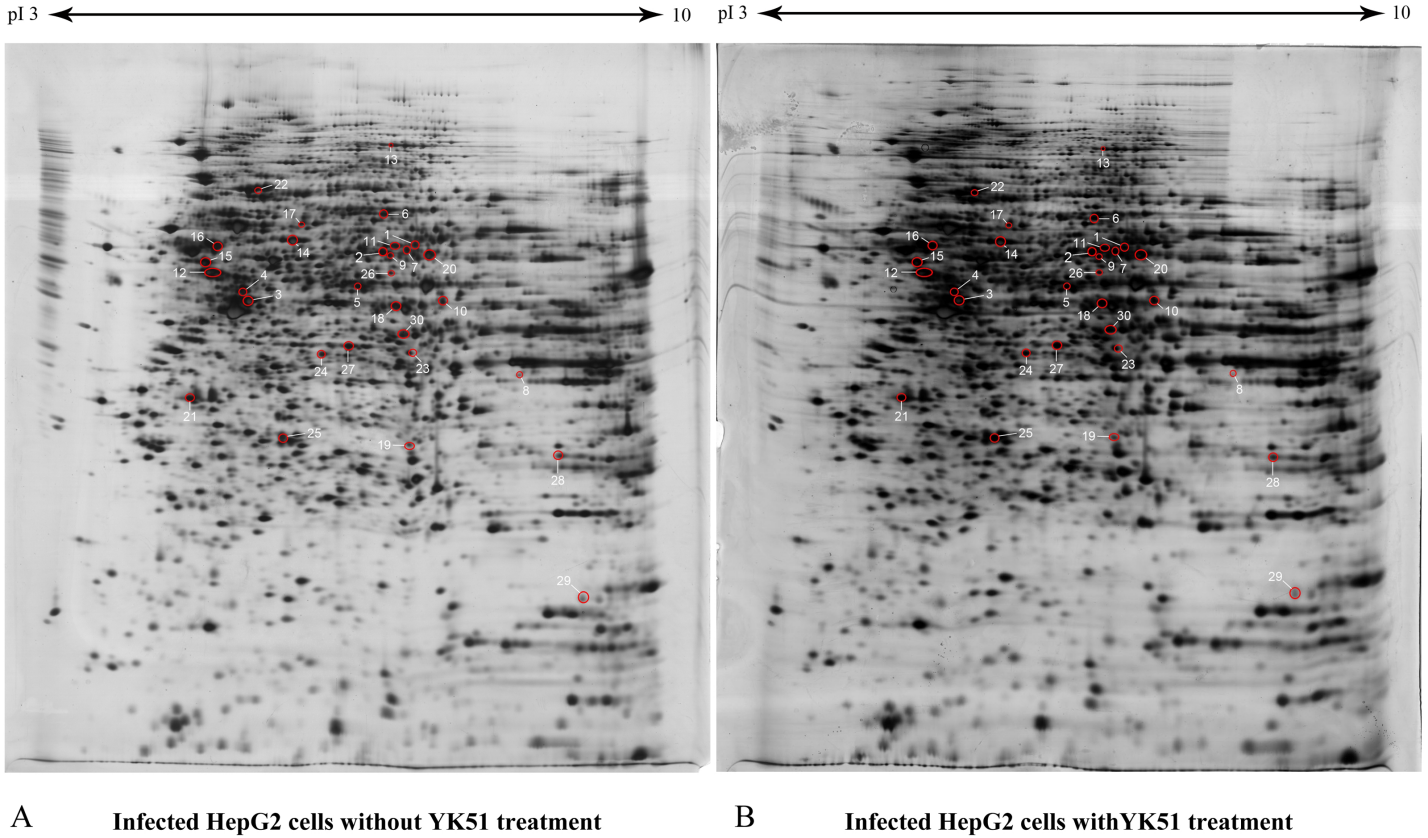
The proteome maps of differentially expressed whole cell proteins in DENV2-infected HepG2 cells with and without YK51 treatment. HepG2 proteomes (40 µg) were profiled using 13 cm, pH 3 to 10 linear IPG strip and 12.5% SDS gel. There were 30 identified protein spots in the gel map. Red circles denote identified protein spots which changed in abundance compared to proteome of DENV2-infected HepG2 cells without YK51 treatment. The spot numbers correspond to spot numbers in Table 2.

We then proceeded to compare the proteome profiles of DENV2+YK51 versus DENV2-YK51 HepG2 cells. A total of 30 protein spots (≥ 1.5 folds with an ANOVA *p*-value ≤0.05) showed a significant decrease in abundance in DENV+YK51 (Table 2). Figure 5 showed the proteome maps of differentially expressed whole cell proteins in DENV2-infected HepG2 cells with and without YK51 treatment. These spots were subsequently excised and subjected to mass spectrometric protein identification.

**Table 2:** List of differentially expressed proteins in the DENV2-infected HepG2 cell with YK51 treatment.

SSpot #	Protein name (Gene symbols)	Swiss-Prot accession	Log normalized volume ± SD^a^		Fold change^b^	Student’s *t*-test	Distinct sum MS/MS score^c^	% Seq/ Matched peptides^d^	MW (kDa)/pI^e^
			Infected, Untreated (*n* = 5)	Infected, Treated (*n* = 5)					
1	Retinal dehydrogenase 1 (AL1A1)	P00352	6.64 ± 0.26	6.32 ± 0.20	−2.1	0.02	58.92	9/5	54.9/6.3
2	Retinal dehydrogenase 1 (AL1A1)	P00352	6.98 ± 0.18	6.63 ± 0.29	−2.1	0.01	328.30	39/20	54.9/6.3
3	Interleukin enhancer-binding factor 2 (ILF2)	Q12905	7.15 ± 0.18	6.79 ± 0.37	−2.0	0.03	134.79	27/10	44.7/5.2
4	Eukaryotic initiation factor 4A-I (eIF4A-1)	P60842	6.57 ± 0.16	6.25 ± 0.24	−2.0	0.01	291.69	45/19	46.2/5.3
5	Proliferation-associated protein 2G4 (PA2G4)	Q9UQ80	6.60 ± 0.19	6.26 ± 0.31	−2.0	0.02	77.32	13/4	43.8/6.1
6	Inosinicase (PUR9)	P31939	6.73 ± 0.13	6.43 ± 0.19	−2.0	0.01	359.25	28/24	64.6/6.27
7	Alanine aminotransferase 2 (ALAT2)	Q8TD30	6.97 ± 0.15	6.66 ± 0.25	−1.9	0.01	143.27	19/11	57.9/7.9
8	ATP synthase subunit beta (ATPB)	P06576	6.53 ± 0.14	6.29 ± 0.11	−1.8	0.00	23.53	6/2	56.9/5.4
9	leucineaminopeptidase 3 (AMPL)	P28838	6.54 ± 0.16	6.25 ± 0.32	−1.8	0.04	138.69	26/11	56.2/8.0
10	26S protease regulatory subunit 8 (PRS8)	P62195	6.78 ± 0.22	6.53 ± 0.17	−1.8	0.03	55.22	13/5	45.6/7.11
11	Glucose-6-phosphate 1-dehydrogenase (G6PDH)	P11413	6.91 ± 0.14	6.62 ± 0.25	−1.8	0.02	141.97	18/11	62.5/8.23
12	Protein disulfide-isomerase A6 (PDIA6)	Q15084	7.48 ± 0.17	7.21 ± 0.17	−1.8	0.01	281.29	33/18	53.9/5.17
13	Elongation factor 2 (EF-2)	P13639	6.54 ± 0.01	6.28 ± 0.21	−1.8	0.01	227	19/17	95.3/6.41
14	Protein disulfide-isomerase A3 (PDIA3)	P30101	7.32 ± 0.15	7.10 ± 0.20	−1.7	0.03	314.27	40/21	56.8/5.99
15	ATP synthase subunit beta (ATPB)	P06576	7.26 ± 0.17	7.04 ± 0.18	−1.7	0.03	270.75	41/15	56.6/5.26
16	Tubulin alpha-1B chain (TBA1B)	P68363	7.28 ± 0.17	7.03 ± 0.27	−1.7	0.04	415.15	55/23	50.2/4.94
17	T-complex protein 1 subunit alpha (TCP1A)	P17987	6.59 ± 0.01	6.33 ± 0.21	−1.7	0.01	66.01	8/4	60.3/5.8
18	Elongation factor Tu(EF-Tu)	P49411	7.06 ± 0.17	6.82 ± 0.24	−1.7	0.04	356.83	48/21	49.9/7.26
19	Protein disulfide-isomerase A6 (PDIA6)	Q15084	6.83 ± 0.12	6.62 ± 0.01	−1.7	0.00	28.31	7/2	48.1/4.95
20	Glutamate dehydrogenase 1 (GDH 1)	P00367	7.24 ± 0.14	7.01 ± 0.17	−1.5	0.05	200.33	31/12	61.4/7.66
21	Annexin A5 (ANXA5)	P08758	7.22 ± 0.01	7.03 ± 0.17	−1.5	0.02	224.97	39/14	35.9/4.94
22	Heat shock 70 kDa protein 9 (HSPA9)	P38646	7.23 ± 0.01	7.04 ± 0.20	−1.5	0.03	197.84	24/21	73.9/6.04
23	26S proteasome non-ATPase regulatory subunit 7 (PSMD7)	P51665	6.87 ± 0.01	6.70 ± 0.01	−1.5	0.00	59.57	19/5	37.0/6.29
24	Transaldolase, 1 (TALDO1)	P37837	7.09 ± 0.10	6.90 ± 0.19	−1.5	0.03	25.11	5/2	37.5/6.36
25	Proteasome activator complex subunit 1 (PSME1)	Q06323	7.30 ± 0.15	7.12 ± 0.12	−1.5	0.02	20.02	9/2	28.7/5.78
26	ATP-citrate synthase, isoform X1 (ACL)	P53396	6.50 ± 0.12	6.33 ± 0.13	−1.5	0.02	133.61	8/9	12.6/8.40
27	biliverdin IX alpha reductase (BVR A)	P53004	7.36 ± 0.01	7.19 ± 0.12	−1.5	0.01	39.14	9/3	33.5/5.91
28	Electron-transfer-flavoprotein, beta polypeptide (EFTB)	P38117	7.30 ± 0.13	7.13 ± 0.10	−1.5	0.01	50.81	14/4	27.9/8.55
29	Actin-related protein 2/3 complex subunit 4 (p20-ARC)	P59998	7.12 ± 0.12	6.94 ± 0.12	−1.5	0.01	49.75	22/4	19.7/8.54
30	Mitogen-activated protein kinase 1 (MAPK 1)	P28482	7.07 ± 0.12	6.87 ± 0.19	−1.5	0.03	62.04	16/4	41.4/6.5

**Notes.**

a Logarithm of normalized spot volume with standard deviation.

b Negative sign (−) of fold change signifies decreased abundance of identified proteins compared to DENV2-infected HepG2 without YK51 treatment.

c Sum of the *z*-scores from each distinct detected peptide in a same protein species.

d Percentage of protein sequence coverage (% Seq) and number of distinct detected peptides.

e Molecular weight of protein in kilo Dalton and isoelectric focusing point of a protein.

### Protein identification and *In silico* analysis

**Figure 6:**
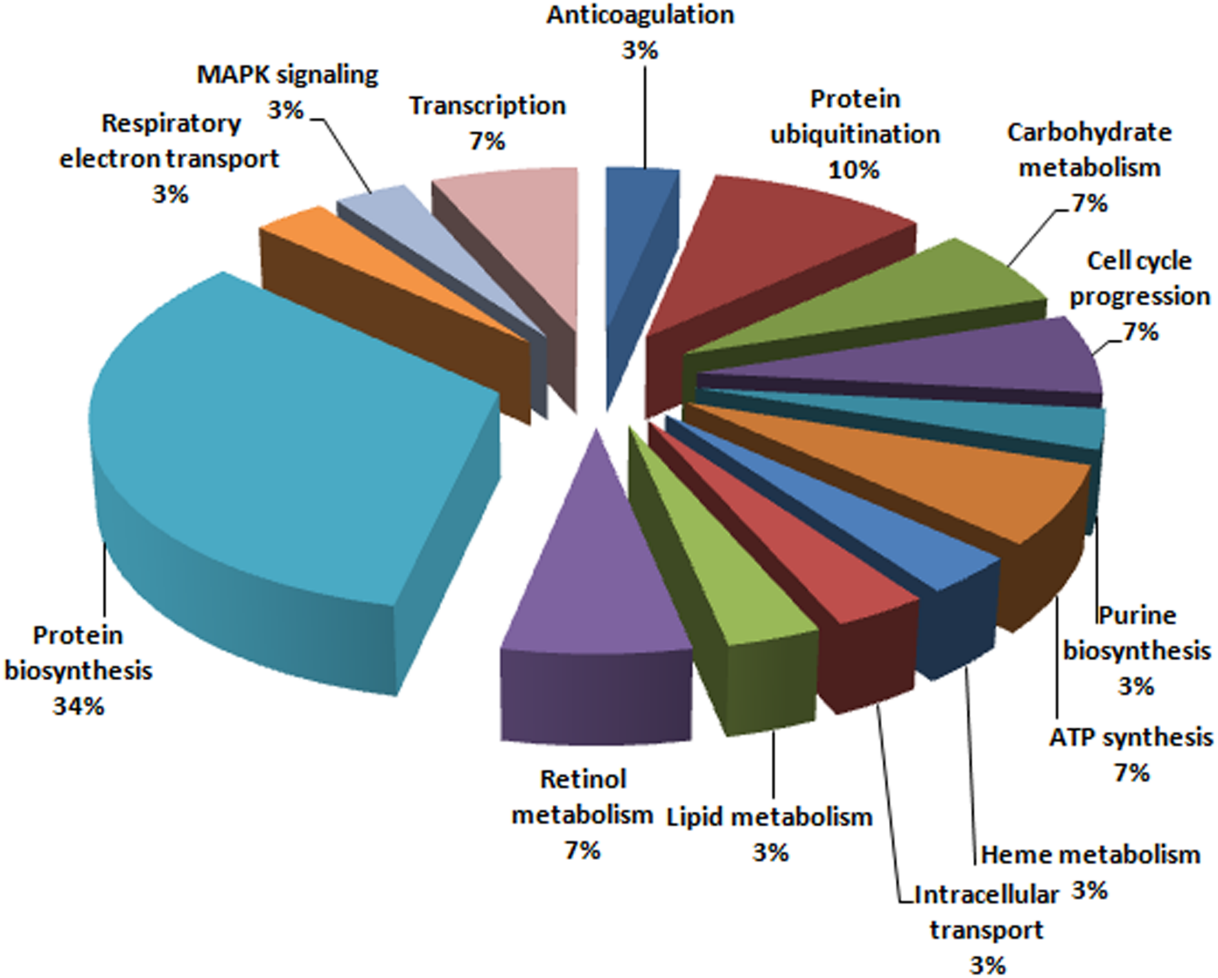
Functional categorization of identified protein spots.

Eight protein spots in gel image of HepG2 cells with YK51 treatment were successfully identified. Pathway analysis showed that these proteins were involved in cell proliferation and cell death, shown in Table 3. Besides that, all 30 protein spots in the gel image of DENV2-infected HepG2 with YK51 treatment were also successfully identified by LC-MS/MS and their identities are listed in Table 2. We then categorized the identified proteins in the DENV2-infected HepG2 with YK51 treatment based on their known functions refer to the Gene Ontology (GO) database (Fig. 6). These proteins were grouped into functional categories, such as protein biosynthesis, which constituted 34% of the total altered proteins, followed by proteins involved in protein ubiquitination (10%). The analysis suggested that these biological functions may be affected by treatment of DENV2-infected HepG2 cells with YK51 compound. This may or may not be related to the antiviral properties of YK51 compound.

In addition, all 30 identified proteins were also subjected to analysis with the IPA software. Eight proteins (GPT2, HSPA9, MAPK1, PDIA3, PDIA6, PSMC5, TALDO1, and ATP5B) were predicted/known to be involved in viral infection (Table 3). IPA predicted that down-regulation of these proteins collectively inhibit viral infection (activation *z*-score as −2.619 and *p*-value of 0.007). Due to their possible role(s) in viral inhibition, transcript analysis was performed to determine if the regulation of protein expression for these proteins were at the transcript level.

**Table 3:** List of predicted molecular functions with a group of identified proteins in the DENV2-infected and not infected HepG2 cells with YK51 treatment based on Ingenuity Pathway analysis.

Samples	Gene names	Gene symbols	Types	Locations	Diseases and functions annotation	
DENV2-infected HepG2 cells with YK51 treatment	Protein disulfide isomerase family A, member 6	PDIA6	Enzyme	Cytoplasm	Viral Infection	
	Protein disulfide isomerase family A, member 3	PDIA3	Peptidase	Cytoplasm	
	Proteasome 26S subunit, ATPase, 5	PSMC5	Transcription regulator	Nucleus	
	Mitogen-activated protein kinase 1	MAPK1	Kinase	Cytoplasm	
	Transaldolase 1	TALDO1	Enzyme	Cytoplasm	
	Glutamic pyruvate transaminase 2	GPT2	Enzyme	Cytoplasm	
	ATP synthase	ATP5B	Transporter	Cytoplasm	
	Heat shock 70kDa protein 9	HSPA9	Other	Cytoplasm	
YK51-treated HepG2 cells	Annexin IV	ANXA4	Other	Plasma membrane	–	Cell death
	COP9 signalosome complex subunit 5	COPS5	Transcription regulator	Nucleus	Proliferation of cells	
	Deoxythymidylate kinase	DTYMK	Kinase	Cytoplasm		
	Protein phosphatase 1, catalytic subunit, alpha isoform	PPP1CA	Phosphatase	Cytoplasm		
	Serine/threonine-protein phosphatase 2A 65 kDa regulatory subunit A alpha isoform	PPP2R1A	Phosphatase	Cytoplasm		

### Transcript analysis

The transcript expression of 8 targeted proteins was evaluated using quantitative Real-Time PCR and normalised against GAPDH. The changes in mRNA expression of three proteins (PDIA6, PDIA3 and PSMC5) were the same with that observed in the proteomic analysis, while the other five showed the opposite direction of expression change (Table 4). RNA levels are known to not always correlate with protein expression levels (Guo et al., 2008). It is because protein may undergo post transcriptional modification become different form of functional proteins even though they are derived from same RNA transcript. As a result, the lower protein abundance of PDIA6, PDIA3, and PSMC5 may partly be explained by the lower expression of their transcript level.

## Discussions

Dengue is not only life-threatening but also an economic burden for both developing and highly-developed countries. As a result, various anti-dengue drug researches have been intensively carried out worldwide to find a cure. Among other natural compounds, 4-hydroxypandurantin-A has been found to strongly inhibit dengue virus serine protease NS2B/NS3. Hence, the synthetic compound YK51 was synthesized to mimic the structure of 4-hydroxypandurantin-A and its effect of inhibition of DENV infection was remarkably demonstrated in the plaque assay (Lee, 2010; Muhamad et al., 2010a). However, the interaction between the host cellular proteins and YK51 compound remain elusive and is one of our main research interests.

As this is the first study on the effects of YK51 compound, the less expensive 2-DGE and silver staining was employed to identify changes in protein expression at different conditions. The whole cell proteome was profiled in a wider range of resolution of 2DE gel to get more protein characters information. Silver staining is the most sensitive colorimetric method that can detect lower protein amount up to nanogram level. However, the use of glutaraldehyde or formaldehyde in staining is incompatible with mass spectrometry which may cause chemical cross linking of the proteins. Therefore, our silver staining protocol had omitted formaldehyde to offer greater MS compatibility at the sacrifice of sensitivity in preparative gels only. In addition, silver stain’s sensitivity to common post-translational modifications, such as glycoproteins and phosphoproteins is greatly reduced due to steric hindrance of glycan-/phospho- moieties on the protein.

Our 2-DGE analysis revealed that YK51 compound slightly affected the proteome profile of uninfected HepG2 cells and pathway analysis showed that the identified proteins are involved in cell proliferation and cell death. Hence, this observation proved that HepG2 cells respond to YK51 treatment by regulating its cell growth and cell death. Besides, in DENV2infected HepG2 cells, the expression of 30 protein spots were decreased in abundance with YK51 treatment. Functional classification of these proteins revealed that most have roles in protein biosynthesis. Host cellular protein biosynthesis machinery is known to be utilized by dengue virus for synthesizing its structural and non-structural proteins. YK51 compound was initially designed as an NS2B/NS3 DENV2 protease inhibitor. However, our results showed that this compound also affected the expression of host proteins that involved in viral infection. Analysis using IPA software suggested that eight of the targeted proteins (ATP5B, GPT2, HSPA9, MAPK1, PDIA3, PDIA6, PSMC5, and TALDO1) were involved in virus infection and their lower protein abundance may inhibit viral infection. Here we discuss the possible roles of these proteins and how they may be involved in virus infection.

ATP5B primarily functions in catalyzing ATP synthesis. It has been proven as one of the dengue NS4B interacting protein targets in mitochondria of liver huh7 cells. DENV NS4B promotes mitochondria elongation which is connected to DENV-induced convoluted membranes leads to evasion of host innate immunity. ATP synthase subunits were suggested to be modulated by DENV to regulate host ATP generation for the biogenesis of membranous replication (Chatel-Chaix et al., 2016). However, other studies revealed that ATP5B may also involve in mediating the entry of chikungunya virus (CHIKV) into insect cells. During virus entry, it can interact with CHIKV and co-localizes in host cells (Fongsaran et al., 2014). Furthermore, ATP5B has also been found to be increased in abundance in avian reovirus infected host cells (Chen et al., 2014).

HSP70 (gene symbol, HSPA9) acts as a chaperone in the mitochondrion. HSP70 has been reported to have roles in many different viral infections. In rabies, the expression of HSP70 was shown to be induced with virus infection and treatment with HSP70 inhibitor (quercetin) resulted in a significant decrease of viral mRNAs, proteins, and particles (Lahaye et al., 2012). Nevertheless, the key roles of Hsp70 in dengue infection have been clearly elucidated by a robust functional study. It has demonstrated that Hsp70 is vital for DENV infection of human and even mosquito cells. It is a key component at DENV virus entry, replication, and other post-entry steps. Small molecule inhibitors targeting Hsp70, like JG40is a highly potential antiviral drug candidate because DENV virus could not develop resistance to them even by 10 virus passages compared to NS5 polymerase inhibitor, 2′ C-methyladenosine. Viral NS5 polymerase can also directly bind to Hsp70 chaperone and gain stability from being degraded in host cell as well as acquisition of its native folded state (Taguwa et al., 2015). Hence, we postulate that YK51 may attenuate DENV infection in liver cells by down-regulate Hsp70 and it could be a potential drug candidate that mutational DENV cannot easily develop resistance to it.

Virulent DENV hijacks the host cellular machinery for virus replication. It has been known to regulate host cellular signaling pathways to complete its replication. The MAPK signalling pathway consists of four common components: the extracellular signal-regulated kinase 1/2 (ERK1/2), ERK5, Jun-N-terminal kinase or stress-activated protein kinase (JNK/SAPK) and p38. They are involved in many cellular functions such as apoptosis, differentiation, proliferation and immune response (DeSilva et al., 1996; Dong, Davis & Flavell, 2002; Pearson et al., 2001). MAPK1 (also known as ERK2) was decreased in abundance with YK51 treatment. Involvement of the MAPK signaling pathway in DENV infection has been reported in patients with severe life-threatening dengue hemorrhagic fever (DHF) (Yeh et al., 2013). It has also been implicated in virus entry via endosomal trafficking and in virus propagation (Ndjomou et al., 2009). MAPK1 has been known as a kinase that can phosphorylate or activate downstream proteins like Mnk kinase, eIF4e and ribosomal protein S6 (RPS6) which are orchestrated in cap-dependent translation (Roux et al., 2007). Up-regulation of eIF4e and other elongation initiation factors were only observed in the early event of DENV infection at/before 24-hour post-infection (Villas-Boas et al., 2009). Therefore, down-regulation of MAPK1 possibly inhibits viral polypeptide translation by dampening host cell translational machinery.

**Table 4:** Transcript levels of eight proteins which were predicted to be involved in virus inhibition.

Gene name	Gene symbol	NCBI RefSeq	Fold change^a^	Student’s *t*-test	Taqman assay ID^b^
Protein disulfide isomerase family A, member 6	PDIA6	NM_005742.2	−2.51	0.002	Hs01012543_m1
Protein disulfide isomerase family A, member 3	PDIA3	NM_005313.4	−2.22	0.000	Hs00607126_m1
Proteasome 26S subunit, ATPase, 5	PSMC5	NM_001199163.1	−2.03	0.036	Hs01029472_g1
Mitogen-activated protein kinase 1	MAPK1	NM_002745.4	1.57	0.001	Hs01046830_m1
Transaldolase 1	TALDO1	NM_006755.1	1.70	0.028	Hs00997203_m1
Glutamic pyruvate transaminase 2	GPT2	NM_133443.2	1.77	0.006	Hs00370287_m1
ATP synthase	ATP5B	NM_001686.3	1.80	0.048	Hs00969569_m1
Heat shock 70 kDa protein 9	HSPA9	NM_004134.6	1.84	0.001	Hs00269818_m1
Glyceraldehyde-3-phosphate dehydrogenase^c^	GAPDH	NM_001256799.1	–	–	Hs02758991_g1

**Notes.**

a Negative value represents decreased abundance of transcripts, whereas positive value represents increased abundance of transcripts.

b Taqman gene assay IDs were derived through Applied Biosystem TaqMan^®^ Gene Expression Assay Search.

c Housekeeping gene for real time PCR assay.

PDIA3 and PDIA6 are membrane associated enzymes of the endoplasmic reticulum and are implicated in translational modification of proteins. PDIs mediate platelet aggregation and platelet adhesion (Essex & Li, 1999; Essex et al., 2001; Lahav et al., 2000; Manickam et al., 2008) and a study showed that inhibition of PDI using anti-DENV NS1 antibodies leading to the inhibition of platelet aggregation (Cheng et al., 2009). Most of the researches always suggest PDI has a vital role in the presentation of DENV infection symptoms. Intriguingly, our result showed that up-regulation of PDI may also facilitate DENV infection in liver cells. This result was in coherent with 1DLC-MS/MS proteomic results of a study on DENV infected HepG2 secretome (Higa et al., 2008). Cell surface PDI have been suggested to be involved in facilitating DENV entry/infection in endothelial cells through activation of β1 and β3 integrins (Wan et al., 2012). Similar mechanisms and functions of PDI probably take place in trafficking DENV into liver cells as well.

Several studies also demonstrated the effects of down-regulation of PDIA3 and PDIA6 individually upon virus infection. Decreased abundance of PDIA3 in THP1 cells has been shown to be related to the reduction of DENV replication and associated with the production of TNF-β which is a host defense system involved in the elimination of viruses (Mishra et al., 2012). PDIA6 has also been revealed to be actively expressed in hepatoma cells during Hepatitis C virus replication (Blais et al., 2009).

Furthermore, our analysis found that the abundance of PSMC5, and metabolism-related proteins, TALDO1 and GPT2 were reduced in the infected HepG2 with YK51 treatment. GPT2 plays a role in cell nitrogen metabolism and responsible for catalyzing the reversible transamination between alanine and 2-oxoglutarate to form pyruvate and glutamate. An elevated serum alanine aminotransferase level serves as a marker for hepatitis E virus infection (Adjei et al., 2010). GPT2 and other liver metabolites like bile and bilirubins are essential parts of liver nitrogen metabolism and therefore always the selected markers for liver pathogenesis. An untargeted metabolomic study on DENV infected patient sera has shown the liver metabolites like serotonin, bile and biliverdin were significantly increased (Cui et al., 2017). Elevation of GPT2 in DENV patient sera were also reported in the absence of any fluid leakage or a rise in the haematocrit (Fernando et al., 2016). Moreover, an elevated serum alanine aminotransferase level also serves as a marker for hepatitis E virus infection (Adjei et al., 2010). Taken together, this suggests interplay between YK51 induced reduction of GPT2 and liver metabolites production during dengue infection course that requires further researches to explain.

TALDO1 is involved in the pentose-phosphate pathway that is important to synthesis nucleotide precursors for RNA replication. It has been shown to be increased in abundance in Hepatitis C virus infected hepatoma cells (Diamond et al., 2010) as well as in human monocytic cells infected with dengue 2 virus. The study made a postulation that over expression of transaldolase may enhance the DENV-2 (16681 strain) replication rate (Martínez-Betancur & Martínez-Gutierrez, 2016). In addition, a study related to JC virus showed that its T-antigen was able to regulate host cell metabolism by increasing the expression of TALDO1, and thus enhancing the host cell biosynthesis activities in favor of virus production (Noch et al., 2012).

Proteasome, PSMC5 in the cytoplasm is responsible for the degradation of ubiquitinated proteins. It has been reported to be increased in abundance by 3 folds in HIV-infected host cells (Krishnan & Zeichner, 2004). Inhibition of ubiquitin proteasome using β-lactone has also been reported to hamper DENV virus egress by suppressing transcript level of exoc7, exoc1, TC10 proteins which are required for exocytic vesicle formation and exocytosis (Choy et al., 2015). Furthermore, some of ubiquitination related-proteins in HepG2 cells were found being decreased in abundance upon treatment of YK51 like PSMD7 (Spot 23) and PSME1 (Spot 25). The ubiquitin-proteasome pathway is found important for dengue virus infection in a study on primary human endothelial cells. Inhibition of ubiquitin-proteasome pathway by inhibitor UBEI-41 in the study had caused a significheant reduction in the level of viral protein synthesis and its infectivity (Kanlaya et al., 2010).

## Conclusions

YK51 is a protease inhibitor that has been shown to effectively inhibit DENV2 infection. Comparative proteomics analysis revealed that treatment with YK51 compound has altered the expression of a number of proteins in DENV infected HepG2 cells. This suggested that the antiviral properties of YK51 compound may not only limit to its protease inhibitor activity. YK51 also affected the protein expression in DENV2- infected cells. The change in abundance of eight of the targeted proteins (GPT2, HSPA9, MAPK1, PDIA3, PDIA6, PSMC5, TALDO1, and ATP5B) was predicted to collectively inhibit viral infection (pathway analysis). At this point however, further functional characterization will be necessary before this possibility can be confirmed. Targeted knockdown experiments may be performed on these proteins individually using RNAi interference and subsequently evaluate the functional effects on virus replication, entry or assemblies.

##  Supplemental Information

10.7717/peerj.3939/supp-1 Table S1 List of differentially expressed proteins in the YK51-treated HepG2 cells and their identified peptide sequences

10.7717/peerj.3939/supp-2 Table S2 List of differentially expressed proteins in the DENV2-infected HepG2 cells treated with YK51 compound and their identified peptide sequences

10.7717/peerj.3939/supp-3 Table S3 List of identified protein spots in Table 2 and their functional categorization

10.7717/peerj.3939/supp-4 Supplemental Information 1 MSMS spectrum of MAPK1

## Additional Information and Declarations

### Data Availability

The following information was supplied regarding data availability: The raw data has been provided in  Supplemental Files.
