# Supplementary material for: Comparative proteomics reveals that YK51, a 4-Hydroxypandurantin-A analogue, downregulates the expression of proteins associated with dengue virus infection
Source: PeerJ. 2018 Jan 30;5:e3939. doi: 10.7717/peerj.3939 (PMC5796277; doi:10.7717/peerj.3939)
Supplement: Table S1 [file peerj-06-3939-s001.docx]

**Supplementary Table S1:** List of differentially expressed proteins in the YK51-treated HepG2 cells and their identified peptide sequences.

| **Spot ID** | **Protein Name** | **Score** | **Fwd-Rev Score** | **SPI%** | **Retention Time (min)** | **Identified Peptides + Modification** |
| --- | --- | --- | --- | --- | --- | --- |
| 1 | T-complex protein 1 | 18.91 | 8.55 | 84.5 | 13.91 | (R)LVPGGGATEIELAK(Q) |
|  | subunit theta | 15.36 | 15.36 | 79.7 | 15.56 | (K)FAEAFEAIPR(A) |
|  |  | 9.61 | 9.61 | 63.5 | 17.14 | (K)NVGLDIEAEVPAVK(D) |
|  |  | 7.40 | 2.92 | 68.9 | 9.91 | (K)TVGATALPR(L) |
|  |  |  |  |  |  |  |
| 2 | Sepiapterinreductase | 17.47 | 8.76 | 88.7 | 12.28 | (R)AVCLLTGASR(G) |
|  |  | 15.95 | 9.83 | 82.7 | 16.97 | (R)LLLINNAGSLGDVSK(G) |
|  |  | 9.94 | 9.94 | 65.1 | 11.67 | (K)AFPDSPGLNR(T) |
|  |  |  |  |  |  |  |
| 3 | Protein phosphatase 1, | 11.35 | 6.47 | [73.3](file:///E:\millscripts\viewfeed.pl%3fviewer=viewMaster.jar&side=spectrumWin&spectrumFiles=msdataSM\140307-00027-DP\4432\cpick_in\4432-Pos-aMSMS-30_47.6783.6790.0.pkl&mstagHits=NQDLAPNSAEQASILSLVTK&cycle=1&fixedMods=carbamidomethylation&varMods=) | 12.06 | (K)HDLDLICR(A) |
|  | catalytic subunit, alpha | 9.70 | 9.70 | [63.0](file:///E:\millscripts\viewfeed.pl%3fviewer=viewMaster.jar&side=spectrumWin&spectrumFiles=msdataSM\140307-00027-DP\4432\cpick_in\4432-Pos-aMSMS-30_47.6872.6875.0.pkl&mstagHits=ILPTLEAVAALGNK&cycle=1&fixedMods=carbamidomethylation&varMods=) | 17.18 | (R)GVSFTFGAEVVAK(F) |
|  | isoform | 9.04 | 9.04 | [63.6](file:///E:\millscripts\viewfeed.pl%3fviewer=viewMaster.jar&side=spectrumWin&spectrumFiles=msdataSM\140307-00027-DP\4432\cpick_in\4432-Pos-aMSMS-30_47.4363.4393.0.pkl&mstagHits=WFEENASQSTVK&cycle=1&fixedMods=carbamidomethylation&varMods=) | 17.15 | (R)GVSFTFGAEVVAK(F) |
|  |  |  |  |  |  |  |
| 4 | Deoxythymidylate kinase | 21.81 | 21.81 | [94.3](file:///E:\millscripts\viewfeed.pl%3fviewer=viewMaster.jar&side=spectrumWin&spectrumFiles=msdataSM\140307-00027-DP\14188\cpick_in\14188-Pos-aMSMS-30_47.7443.7477.0.pkl&mstagHits=MFVLDEADEMLSR&cycle=1&fixedMods=carbamidomethylation&varMods=) | 13.45 | (K)LSQGVTLVVDR(Y) |
|  |  | 19.91 | 10.66 | [88.9](file:///E:\millscripts\viewfeed.pl%3fviewer=viewMaster.jar&side=spectrumWin&spectrumFiles=msdataSM\140307-00027-DP\14188\cpick_in\14188-Pos-aMSMS-30_47.4381.4411.2.pkl&mstagHits=GYDVIAQAQSGTGK&cycle=1&fixedMods=carbamidomethylation&varMods=) | 15.38 | (R)YAFSGVAFTGAK(E) |
|  |  | 19.23 | 13.02 | [86.4](file:///E:\millscripts\viewfeed.pl%3fviewer=viewMaster.jar&side=spectrumWin&spectrumFiles=msdataSM\140307-00027-DP\14188\cpick_in\14188-Pos-aMSMS-30_47.5181.5184.0.pkl&mstagHits=GIYAYGFEKPSAIQQR&cycle=1&fixedMods=carbamidomethylation&varMods=) | 9.72 | (K)SIEAVHEDIR(V) |
|  |  | 17.91 | 5.87 | [89.1](file:///E:\millscripts\viewfeed.pl%3fviewer=viewMaster.jar&side=spectrumWin&spectrumFiles=msdataSM\140307-00027-DP\14188\cpick_in\14188-Pos-aMSMS-30_47.6288.6343.2.pkl&mstagHits=VLITTDLLAR&cycle=1&fixedMods=carbamidomethylation&varMods=) | 12.82 | (K)LLSSYLQK(K) |
|  |  | 15.02 | 5.30 | [89.3](file:///E:\millscripts\viewfeed.pl%3fviewer=viewMaster.jar&side=spectrumWin&spectrumFiles=msdataSM\140307-00027-DP\14188\cpick_in\14188-Pos-aMSMS-30_47.4766.4816.0.pkl&mstagHits=ATQALVLAPTR&cycle=1&fixedMods=carbamidomethylation&varMods=) | 9.95 | (R)VLSEDAIR(T) |
|  |  | 12.47 | 6.69 | [77.0](file:///E:\millscripts\viewfeed.pl%3fviewer=viewMaster.jar&side=spectrumWin&spectrumFiles=msdataSM\140307-00027-DP\14188\cpick_in\14188-Pos-aMSMS-30_47.4861.4866.2.pkl&mstagHits=KEELTLEGIR&cycle=1&fixedMods=carbamidomethylation&varMods=) | 16.66 | (R)GALIVLEGVDR(A) |
|  |  | 8.27 | 8.27 | [74.6](file:///E:\millscripts\viewfeed.pl%3fviewer=viewMaster.jar&side=spectrumWin&spectrumFiles=msdataSM\140307-00027-DP\14188\cpick_in\14188-Pos-aMSMS-30_47.4451.4534.0.pkl&mstagHits=KVDWLTEK&cycle=1&fixedMods=carbamidomethylation&varMods=) | 15.31 | (R)RGALIVLEGVDR(A) |
|  |  |  |  |  |  |  |
| 5 | Platelet-activating factor | 14.79 | 14.79 | [90.9](file:///E:\millscripts\viewfeed.pl%3fviewer=viewMaster.jar&side=spectrumWin&spectrumFiles=msdataSM\140307-00027-DP\16925\cpick_in\16925-Pos-aMSMS-30_47.6885.6923.0.pkl&mstagHits=SLVEASSSGVSVLSLCEK&cycle=1&fixedMods=carbamidomethylation&varMods=) | 18.64 | (R)VVVLGLLPR(G) |
|  | acetylhydrolase IB | 12.21 | 12.21 | [69.0](file:///E:\millscripts\viewfeed.pl%3fviewer=viewMaster.jar&side=spectrumWin&spectrumFiles=msdataSM\140307-00027-DP\16925\cpick_in\16925-Pos-aMSMS-30_47.6284.6305.0.pkl&mstagHits=ITSGPFEPDLYK&cycle=1&fixedMods=carbamidomethylation&varMods=) | 10.23 | (R)LGYTPVCR(A) + C |
|  | subunit gamma | 11.53 | 5.77 | [80.2](file:///E:\millscripts\viewfeed.pl%3fviewer=viewMaster.jar&side=spectrumWin&spectrumFiles=msdataSM\140307-00027-DP\16925\cpick_in\16925-Pos-aMSMS-30_47.7553.7553.0.pkl&mstagHits=HELLQPFNVLYEK&cycle=1&fixedMods=carbamidomethylation&varMods=) | 13.01 | (K)AIVQLVNER(Q) |
|  |  |  |  |  |  |  |
| 6 | COP9 signalosome | 15.25 | 4.79 | [88.8](file:///E:\millscripts\viewfeed.pl%3fviewer=viewMaster.jar&side=spectrumWin&spectrumFiles=msdataSM\140210-00020-DP\18665\cpick_in\18665-Pos-aMSMS-30_47.6781.6826.2.pkl&mstagHits=NLTALGLNLVASGGTAK&cycle=1&fixedMods=carbamidomethylation&varMods=) | 17.19 | (K)ISALALLK(M) |
|  | complex subunit 5 | 14.55 | 4.89 | [86.4](file:///E:\millscripts\viewfeed.pl%3fviewer=viewMaster.jar&side=spectrumWin&spectrumFiles=msdataSM\140210-00020-DP\18665\cpick_in\18665-Pos-aMSMS-30_47.6979.7004.0.pkl&mstagHits=DVSELTGFPEMLGGR&cycle=1&fixedMods=carbamidomethylation&varMods=) | 12.88 | (K)VNLGAFR(T) |
|  |  | 14.40 | 14.40 | [77.6](file:///E:\millscripts\viewfeed.pl%3fviewer=viewMaster.jar&side=spectrumWin&spectrumFiles=msdataSM\140210-00020-DP\18665\cpick_in\18665-Pos-aMSMS-30_47.7398.7438.3.pkl&mstagHits=ALFEEVPELLTEAEKK&cycle=1&fixedMods=carbamidomethylation&varMods=) | 8.40 | (K)LEQSEAQLGR(G) |
|  |  |  |  |  |  |  |
| 7 | Annexin IV | 13.16 | 8.66 | [71.2](file:///E:\millscripts\viewfeed.pl%3fviewer=viewMaster.jar&side=spectrumWin&spectrumFiles=msdataSM\140210-00020-DP\5951\cpick_in\5951-Pos-aMSMS-30_47.3174.3191.0.pkl&mstagHits=EGTYHFR&cycle=1&fixedMods=carbamidomethylation&varMods=) | 12.37 | (K)GLGTDDNTLIR(V) |
|  |  | 8.55 | 8.55 | [76.9](file:///E:\millscripts\viewfeed.pl%3fviewer=viewMaster.jar&side=spectrumWin&spectrumFiles=msdataSM\140210-00020-DP\5951\cpick_in\5951-Pos-aMSMS-30_47.5429.5432.0.pkl&mstagHits=AGEIELELQR&cycle=1&fixedMods=carbamidomethylation&varMods=) | 12.48 | (R)VLVSLSAGGR(D) |
|  |  | 8.19 | 8.19 | [60.2](file:///E:\millscripts\viewfeed.pl%3fviewer=viewMaster.jar&side=spectrumWin&spectrumFiles=msdataSM\140210-00020-DP\5951\cpick_in\5951-Pos-aMSMS-30_47.5843.5855.0.pkl&mstagHits=VLCIINPGNPTGQVQSR&cycle=1&fixedMods=carbamidomethylation&varMods=) | 14.90 | (K)FLTVLCSR(N) + C |
|  |  |  |  |  |  |  |
| 8 | Serine/threonine-protein | 20.50 | 17.10 | [94.7](file:///E:\millscripts\viewfeed.pl%3fviewer=viewMaster.jar&side=spectrumWin&spectrumFiles=msdataSM\140307-00027-DP\2575\cpick_in\2575-Pos-aMSMS-30_47.8371.8371.0.pkl&mstagHits=VALTGLTVAEYFR&cycle=1&fixedMods=carbamidomethylation&varMods=) | 14.85 | (R)LAGGDWFTSR(T) |
|  | phosphatase 2A 65 kDa | 17.31 | 17.31 | [83.0](file:///E:\millscripts\viewfeed.pl%3fviewer=viewMaster.jar&side=spectrumWin&spectrumFiles=msdataSM\140307-00027-DP\2575\cpick_in\2575-Pos-aMSMS-30_47.7972.7972.0.pkl&mstagHits=AIAELGIYPAVDPLDSTSR&cycle=1&fixedMods=carbamidomethylation&varMods=) | 17.84 | (K)LSTIALALGVER(T) |
|  | regulatory subunit A | 17.13 | 17.13 | 81.6 | 10.82 | (R)NLCSDDTPMVR(R) + C |
|  | alpha isoform | 16.96 | 16.96 | 84.7 | 19.60 | (R)ENVIMSQILPCIK(E) + C |
|  |  | 16.84 | 16.84 | 73.9 | 16.51 | (R)TSACGLFSVCYPR(V) + C |
|  |  | 16.04 | 16.04 | 79.1 | 18.48 | (K)IGPILDNSTLQSEVKPILEK  (L) |
|  |  | 15.88 | 8.42 | 90.8 | 12.14 | (K)VLELDNVK(S) |
|  |  | 15.69 | 6.94 | 86.4 | 8.52 | (K)AVGPEITK(T) |
|  |  | 15.63 | 15.63 | 81.6 | 7.98 | (K)LTQDQDVDVK(Y) |
|  |  | 14.64 | 10.26 | 85.8 | 19.63 | (K)TDLVPAFQNLMK(D) |
|  |  | 14.35 | 14.35 | 83.7 | 9.95 | (R)MAGDPVANVR(F) |
|  |  | 14.16 | 11.14 | 76.7 | 7.82 | (K)ELVSDANQHVK(S) |
|  |  | 12.77 | 12.77 | 67.4 | 10.75 | (K)EFCENLSADCR(E) + C |
|  |  | 11.70 | 11.70 | 72.3 | 12.78 | (K)EWAHATIIPK(V) |
|  |  | 11.32 | 11.32 | 70.3 | 16.74 | (K)KLSTIALALGVER(T) |
|  |  | 10.63 | 10.63 | 64.2 | 10.73 | (K)EFCENLSADCR(E) + C |
|  |  | 9.86 | 9.86 | 65.3 | 13.85 | (R)YMVADKFTELQK(A) |
|  |  | 9.41 | 9.41 | 63.8 | 8.01 | (R)NEDVQLR(L) |
|  |  |  |  |  |  |  |

* **C** represents carbamidomethylation modification on peptide sequence.
