# Supplementary material for: Comparative proteomics reveals that YK51, a 4-Hydroxypandurantin-A analogue, downregulates the expression of proteins associated with dengue virus infection
Source: PeerJ. 2018 Jan 30;5:e3939. doi: 10.7717/peerj.3939 (PMC5796277; doi:10.7717/peerj.3939)
Supplement: Table S2 [file peerj-06-3939-s002.docx]

**Supplementary Table S2:** List of differentially expressed proteins in the DENV2-infected HepG2 cells treated with YK51 compound and their identified peptide sequences.

| **Spot ID** | **Protein Name** | **Score** | **Fwd-Rev Score** | **SPI%** | **Retention Time (min)** | **Identified Peptides + Modification** |
| --- | --- | --- | --- | --- | --- | --- |
| 1 | Retinal Dehydrogenase 1 | 16.49 | 5.28 | 78.9 | 10.58 | (K)LADLIER(D) |
|  |  | 12.37 | 9.35 | 63.7 | 13.20 | (R)TIPIDGNFFTYTR(H) |
|  |  | 10.95 | 9.76 | 65.4 | 11.76 | (R)VTLELGGK(S) |
|  |  | 9.76 | 6.79 | 74.4 | 11.56 | (K)ILDLIESGKK(E) |
|  |  | 9.35 | 6.33 | 91.8 | 18.63 | (K)SLDDVIKR(A) |
|  |  |  |  |  |  |  |
| 2 | Retinal Dehydrogenase 1 | 22.05 | 22.05 | 93.4 | 18.08 | (K)LYSNAYLNDLAGCIK(T) + C* |
|  |  | 21.84 | 21.84 | 95.9 | 20.17 | (R)IFVEESIYDEFVR(R) |
|  |  | 20.80 | 20.80 | 85.6 | 13.77 | (K)IFINNEWHDSVSGK(K) |
|  |  | 20.72 | 14.52 | 91.5 | 13.72 | (R)ELGEYGFHEYTEVK(T) |
|  |  | 20.07 | 20.07 | 93.9 | 18.75 | (R)TIPIDGNFFTYTR(H) |
|  |  | 20.02 | 20.02 | [90.7](file:///E:\millscripts\viewfeed.pl%3fviewer=viewMaster.jar&side=spectrumWin&spectrumFiles=msdataSM\140307-00027-DP\19910\cpick_in\19910-Pos-aMSMS-30_47.6670.6755.0.pkl&mstagHits=IAKEEIFGPVQQIMK&cycle=1&fixedMods=carbamidomethylation&varMods=) | 17.66 | (R)IAKEEIFGPVQQIMK(F) |
|  |  | 19.80 | 11.27 | [90.1](file:///E:\millscripts\viewfeed.pl%3fviewer=viewMaster.jar&side=spectrumWin&spectrumFiles=msdataSM\140307-00027-DP\19910\cpick_in\19910-Pos-aMSMS-30_47.4103.4138.2.pkl&mstagHits=VAFTGSTEVGK&cycle=1&fixedMods=carbamidomethylation&varMods=) | 10.14 | (K)VAFTGSTEVGK(L) |
|  |  | 16.82 | 7.53 | [88.3](file:///E:\millscripts\viewfeed.pl%3fviewer=viewMaster.jar&side=spectrumWin&spectrumFiles=msdataSM\140307-00027-DP\19910\cpick_in\19910-Pos-aMSMS-30_47.4292.4297.0.pkl&mstagHits=SLDDVIKR&cycle=1&fixedMods=carbamidomethylation&varMods=) | 10.67 | (K)SLDDVIKR(A) |
|  |  | 16.46 | 5.70 | [89.2](file:///E:\millscripts\viewfeed.pl%3fviewer=viewMaster.jar&side=spectrumWin&spectrumFiles=msdataSM\140307-00027-DP\19910\cpick_in\19910-Pos-aMSMS-30_47.5179.5182.0.pkl&mstagHits=ILDLIESGKK&cycle=1&fixedMods=carbamidomethylation&varMods=) | 13.30 | (K)ILDLIESGKK(E) |
|  |  | 16.15 | 16.15 | [80.7](file:///E:\millscripts\viewfeed.pl%3fviewer=viewMaster.jar&side=spectrumWin&spectrumFiles=msdataSM\140307-00027-DP\19910\cpick_in\19910-Pos-aMSMS-30_47.6129.6132.2.pkl&mstagHits=QAFQIGSPWR&cycle=1&fixedMods=carbamidomethylation&varMods=) | 16.01 | (R)QAFQIGSPWR(T) |
|  |  | 15.91 | 5.25 | [87.8](file:///E:\millscripts\viewfeed.pl%3fviewer=viewMaster.jar&side=spectrumWin&spectrumFiles=msdataSM\140307-00027-DP\19910\cpick_in\19910-Pos-aMSMS-30_47.5605.5608.0.pkl&mstagHits=ILDLIESGK&cycle=1&fixedMods=carbamidomethylation&varMods=) | 14.67 | (K)ILDLIESGK(K) |
|  |  | 15.62 | 15.62 | [67.3](file:///E:\millscripts\viewfeed.pl%3fviewer=viewMaster.jar&side=spectrumWin&spectrumFiles=msdataSM\140307-00027-DP\19910\cpick_in\19910-Pos-aMSMS-30_47.3763.3766.0.pkl&mstagHits=YCAGWADK&cycle=1&fixedMods=carbamidomethylation&varMods=) | 9.23 | (R)YCAGWADK(I) + C |
|  |  | 15.42 | 15.42 | [76.6](file:///E:\millscripts\viewfeed.pl%3fviewer=viewMaster.jar&side=spectrumWin&spectrumFiles=msdataSM\140307-00027-DP\19910\cpick_in\19910-Pos-aMSMS-30_47.6853.6857.0.pkl&mstagHits=EEIFGPVQQIMK&cycle=1&fixedMods=carbamidomethylation&varMods=) | 18.24 | (K)EEIFGPVQQIMK(F) |
|  |  | 14.26 | 3.15 | [85.3](file:///E:\millscripts\viewfeed.pl%3fviewer=viewMaster.jar&side=spectrumWin&spectrumFiles=msdataSM\140307-00027-DP\19910\cpick_in\19910-Pos-aMSMS-30_47.4557.4561.0.pkl&mstagHits=SLDDVIK&cycle=1&fixedMods=carbamidomethylation&varMods=) | 11.45 | (K)SLDDVIK(R) |
|  |  | 13.66 | 3.67 | [94.9](file:///E:\millscripts\viewfeed.pl%3fviewer=viewMaster.jar&side=spectrumWin&spectrumFiles=msdataSM\140307-00027-DP\19910\cpick_in\19910-Pos-aMSMS-30_47.4649.4714.2.pkl&mstagHits=LADLIER&cycle=1&fixedMods=carbamidomethylation&varMods=) | 11.74 | (K)LADLIER(D) |
|  |  | 12.27 | 12.27 | [72.6](file:///E:\millscripts\viewfeed.pl%3fviewer=viewMaster.jar&side=spectrumWin&spectrumFiles=msdataSM\140307-00027-DP\19910\cpick_in\19910-Pos-aMSMS-30_47.6576.6576.0.pkl&mstagHits=YILGNPLTPGVTQGPQIDK&cycle=1&fixedMods=carbamidomethylation&varMods=) | 17.39 | (K)YILGNPLTPGVTQGPQIDK(E) |
|  |  | 12.20 | 12.20 | [75.4](file:///E:\millscripts\viewfeed.pl%3fviewer=viewMaster.jar&side=spectrumWin&spectrumFiles=msdataSM\140307-00027-DP\19910\cpick_in\19910-Pos-aMSMS-30_47.4084.4087.0.pkl&mstagHits=LECGGGPWGNK&cycle=1&fixedMods=carbamidomethylation&varMods=) | 10.07 | (K)LECGGGPWGNK(G) + C |
|  |  | 11.81 | 11.81 | [69.9](file:///E:\millscripts\viewfeed.pl%3fviewer=viewMaster.jar&side=spectrumWin&spectrumFiles=msdataSM\140307-00027-DP\19910\cpick_in\19910-Pos-aMSMS-30_47.6758.6761.0.pkl&mstagHits=ANNTFYGLSAGVFTK&cycle=1&fixedMods=carbamidomethylation&varMods=) | 17.94 | (R)ANNTFYGLSAGVFTK(D) |
|  |  | 11.77 | 11.77 | [67.9](file:///E:\millscripts\viewfeed.pl%3fviewer=viewMaster.jar&side=spectrumWin&spectrumFiles=msdataSM\140307-00027-DP\19910\cpick_in\19910-Pos-aMSMS-30_47.4587.4590.2.pkl&mstagHits=VTLELGGK&cycle=1&fixedMods=carbamidomethylation&varMods=) | 11.57 | (R)VTLELGGK(S) |
|  |  | 10.63 | 10.63 | [66.5](file:///E:\millscripts\viewfeed.pl%3fviewer=viewMaster.jar&side=spectrumWin&spectrumFiles=msdataSM\140307-00027-DP\19910\cpick_in\19910-Pos-aMSMS-30_47.6291.6297.0.pkl&mstagHits=YILGNPLTPGVTQGPQIDKEQYDK&cycle=1&fixedMods=carbamidomethylation&varMods=) | 16.60 | (K)YILGNPLTPGVTQGPQIDKEQ YDK(I) |
|  |  |  |  |  |  |  |
| 3 | Interleukin enhancer-binding factor 2 | 20.62 | 14.97 | [94.6](file:///E:\millscripts\viewfeed.pl%3fviewer=viewMaster.jar&side=spectrumWin&spectrumFiles=msdataSM\140307-00027-DP\4432\cpick_in\4432-Pos-aMSMS-30_47.6783.6790.0.pkl&mstagHits=NQDLAPNSAEQASILSLVTK&cycle=1&fixedMods=carbamidomethylation&varMods=) | 19.42 | (R)NQDLAPNSAEQASILSLVTK  (I) |
|  |  | 20.27 | 20.27 | [89.3](file:///E:\millscripts\viewfeed.pl%3fviewer=viewMaster.jar&side=spectrumWin&spectrumFiles=msdataSM\140307-00027-DP\4432\cpick_in\4432-Pos-aMSMS-30_47.6872.6875.0.pkl&mstagHits=ILPTLEAVAALGNK&cycle=1&fixedMods=carbamidomethylation&varMods=) | 19.67 | (K)ILPTLEAVAALGNK(V) |
|  |  | 16.71 | 16.71 | [66.2](file:///E:\millscripts\viewfeed.pl%3fviewer=viewMaster.jar&side=spectrumWin&spectrumFiles=msdataSM\140307-00027-DP\4432\cpick_in\4432-Pos-aMSMS-30_47.4363.4393.0.pkl&mstagHits=WFEENASQSTVK&cycle=1&fixedMods=carbamidomethylation&varMods=) | 11.76 | (R)WFEENASQSTVK(V) |
|  |  | 13.88 | 13.88 | [82.9](file:///E:\millscripts\viewfeed.pl%3fviewer=viewMaster.jar&side=spectrumWin&spectrumFiles=msdataSM\140307-00027-DP\4432\cpick_in\4432-Pos-aMSMS-30_47.5710.5716.0.pkl&mstagHits=ILITTVPPNLR&cycle=1&fixedMods=carbamidomethylation&varMods=) | 16.16 | (K)ILITTVPPNLR(K) |
|  |  | 12.64 | 12.64 | [66.9](file:///E:\millscripts\viewfeed.pl%3fviewer=viewMaster.jar&side=spectrumWin&spectrumFiles=msdataSM\140307-00027-DP\4432\cpick_in\4432-Pos-aMSMS-30_47.5502.5512.0.pkl&mstagHits=VKPAPDETSFSEALLK&cycle=1&fixedMods=carbamidomethylation&varMods=) | 15.51 | (R)VKPAPDETSFSEALLK(R) |
|  |  | 12.33 | 12.33 | [67.8](file:///E:\millscripts\viewfeed.pl%3fviewer=viewMaster.jar&side=spectrumWin&spectrumFiles=msdataSM\140307-00027-DP\4432\cpick_in\4432-Pos-aMSMS-30_47.5152.5155.0.pkl&mstagHits=QPLALNVAYR&cycle=1&fixedMods=carbamidomethylation&varMods=) | 14.42 | (R)QPLALNVAYR(R) |
|  |  | 11.94 | 5.05 | [79.2](file:///E:\millscripts\viewfeed.pl%3fviewer=viewMaster.jar&side=spectrumWin&spectrumFiles=msdataSM\140307-00027-DP\4432\cpick_in\4432-Pos-aMSMS-30_47.5201.5206.0.pkl&mstagHits=VLQSALAAIR&cycle=1&fixedMods=carbamidomethylation&varMods=) | 14.59 | (K)VLQSALAAIR(H) |
|  |  | 10.83 | 1.68 | [74.2](file:///E:\millscripts\viewfeed.pl%3fviewer=viewMaster.jar&side=spectrumWin&spectrumFiles=msdataSM\140307-00027-DP\4432\cpick_in\4432-Pos-aMSMS-30_47.5112.5119.0.pkl&mstagHits=KLDPELHLDIK&cycle=1&fixedMods=carbamidomethylation&varMods=) | 14.27 | (R)KLDPELHLDIK(V) |
|  |  | 9.82 | 9.82 | [66.3](file:///E:\millscripts\viewfeed.pl%3fviewer=viewMaster.jar&side=spectrumWin&spectrumFiles=msdataSM\140307-00027-DP\4432\cpick_in\4432-Pos-aMSMS-30_47.5854.5854.0.pkl&mstagHits=ILITTVPPNLR&cycle=1&fixedMods=carbamidomethylation&varMods=) | 16.73 | (K)ILITTVPPNLR(K) |
|  |  | 8.69 | 8.69 | [60.2](file:///E:\millscripts\viewfeed.pl%3fviewer=viewMaster.jar&side=spectrumWin&spectrumFiles=msdataSM\140307-00027-DP\4432\cpick_in\4432-Pos-aMSMS-30_47.6970.6970.0.pkl&mstagHits=ILPTLEAVAALGNK&cycle=1&fixedMods=carbamidomethylation&varMods=) | 19.91 | (K)ILPTLEAVAALGNK(V) |
|  |  | 8.59 | 8.59 | [63.5](file:///E:\millscripts\viewfeed.pl%3fviewer=viewMaster.jar&side=spectrumWin&spectrumFiles=msdataSM\140307-00027-DP\4432\cpick_in\4432-Pos-aMSMS-30_47.5420.5420.0.pkl&mstagHits=LDPELHLDIK&cycle=1&fixedMods=carbamidomethylation&varMods=) | 15.24 | (K)LDPELHLDIK(V) |
|  |  | 6.98 | 6.98 | [69.1](file:///E:\millscripts\viewfeed.pl%3fviewer=viewMaster.jar&side=spectrumWin&spectrumFiles=msdataSM\140307-00027-DP\4432\cpick_in\4432-Pos-aMSMS-30_47.3208.3208.0.pkl&mstagHits=VVESLR&cycle=1&fixedMods=carbamidomethylation&varMods=) | 7.78 | (K)VVESLR(A) |
|  |  |  |  |  |  |  |
| 4 | Eukaryotic initiation | 21.43 | 21.43 | [92](file:///E:\millscripts\viewfeed.pl%3fviewer=viewMaster.jar&side=spectrumWin&spectrumFiles=msdataSM\140307-00027-DP\14188\cpick_in\14188-Pos-aMSMS-30_47.7443.7477.0.pkl&mstagHits=MFVLDEADEMLSR&cycle=1&fixedMods=carbamidomethylation&varMods=).0 | 20.12 | (R)VLITTDLLAR(G) |
|  | factor 4A-I | 21.09 | 21.09 | [97.6](file:///E:\millscripts\viewfeed.pl%3fviewer=viewMaster.jar&side=spectrumWin&spectrumFiles=msdataSM\140307-00027-DP\14188\cpick_in\14188-Pos-aMSMS-30_47.4381.4411.2.pkl&mstagHits=GYDVIAQAQSGTGK&cycle=1&fixedMods=carbamidomethylation&varMods=) | 11.24 | (R)VLITTDLLAR(G) |
|  |  | 19.14 | 19.14 | [84.2](file:///E:\millscripts\viewfeed.pl%3fviewer=viewMaster.jar&side=spectrumWin&spectrumFiles=msdataSM\140307-00027-DP\14188\cpick_in\14188-Pos-aMSMS-30_47.5181.5184.0.pkl&mstagHits=GIYAYGFEKPSAIQQR&cycle=1&fixedMods=carbamidomethylation&varMods=) | 14.02 | (R)VFDMLNR(R) |
|  |  | 18.74 | 10.00 | [91.3](file:///E:\millscripts\viewfeed.pl%3fviewer=viewMaster.jar&side=spectrumWin&spectrumFiles=msdataSM\140307-00027-DP\14188\cpick_in\14188-Pos-aMSMS-30_47.6288.6343.2.pkl&mstagHits=VLITTDLLAR&cycle=1&fixedMods=carbamidomethylation&varMods=) | 17.26 | (R)KVDWLTEK(M) |
|  |  | 17.54 | 17.54 | [94.2](file:///E:\millscripts\viewfeed.pl%3fviewer=viewMaster.jar&side=spectrumWin&spectrumFiles=msdataSM\140307-00027-DP\14188\cpick_in\14188-Pos-aMSMS-30_47.4766.4816.0.pkl&mstagHits=ATQALVLAPTR&cycle=1&fixedMods=carbamidomethylation&varMods=) | 12.61 | (R)KGVAINMVTEEDKR(T) |
|  |  | 16.19 | 9.05 | [90.5](file:///E:\millscripts\viewfeed.pl%3fviewer=viewMaster.jar&side=spectrumWin&spectrumFiles=msdataSM\140307-00027-DP\14188\cpick_in\14188-Pos-aMSMS-30_47.4861.4866.2.pkl&mstagHits=KEELTLEGIR&cycle=1&fixedMods=carbamidomethylation&varMods=) | 12.94 | (R)KGVAINMVTEEDKR(T) |
|  |  | 15.97 | 5.90 | [77.9](file:///E:\millscripts\viewfeed.pl%3fviewer=viewMaster.jar&side=spectrumWin&spectrumFiles=msdataSM\140307-00027-DP\14188\cpick_in\14188-Pos-aMSMS-30_47.4451.4534.0.pkl&mstagHits=KVDWLTEK&cycle=1&fixedMods=carbamidomethylation&varMods=) | 11.54 | (R)KGVAINFVTEEDKR(I) |
|  |  | 15.70 | 15.70 | [73.7](file:///E:\millscripts\viewfeed.pl%3fviewer=viewMaster.jar&side=spectrumWin&spectrumFiles=msdataSM\140307-00027-DP\14188\cpick_in\14188-Pos-aMSMS-30_47.5615.5619.0.pkl&mstagHits=LQMEAPHIIVGTPGR&cycle=1&fixedMods=carbamidomethylation&varMods=) | 15.41 | (R)GIYAYGFEKPSAIQQR(A) |
|  |  | 15.16 | 3.56 | [84.9](file:///E:\millscripts\viewfeed.pl%3fviewer=viewMaster.jar&side=spectrumWin&spectrumFiles=msdataSM\140307-00027-DP\14188\cpick_in\14188-Pos-aMSMS-30_47.5137.5188.2.pkl&mstagHits=AILPCIK&cycle=1&fixedMods=carbamidomethylation&varMods=) | 13.84 | (R)GIYAYGFEKPSAIQQR(A) + C |
|  |  | 15.10 | 7.65 | [79.8](file:///E:\millscripts\viewfeed.pl%3fviewer=viewMaster.jar&side=spectrumWin&spectrumFiles=msdataSM\140307-00027-DP\14188\cpick_in\14188-Pos-aMSMS-30_47.3265.3268.0.pkl&mstagHits=ELAQQIQK&cycle=1&fixedMods=carbamidomethylation&varMods=) | 8.04 | (R)ELAQQIQK(V) |
|  |  | 15.10 | 15.10 | [76.8](file:///E:\millscripts\viewfeed.pl%3fviewer=viewMaster.jar&side=spectrumWin&spectrumFiles=msdataSM\140307-00027-DP\14188\cpick_in\14188-Pos-aMSMS-30_47.5174.5245.0.pkl&mstagHits=GIYAYGFEKPSAIQQR&cycle=1&fixedMods=carbamidomethylation&varMods=) | 13.99 | (R)DFTVSAMHGDMDQK(E) |
|  |  | 15.00 | 15.00 | [73](file:///E:\millscripts\viewfeed.pl%3fviewer=viewMaster.jar&side=spectrumWin&spectrumFiles=msdataSM\140307-00027-DP\14188\cpick_in\14188-Pos-aMSMS-30_47.6031.6067.0.pkl&mstagHits=VVMALGDYMGASCHACIGGTNVR&cycle=1&fixedMods=carbamidomethylation&varMods=).0 | 16.53 | (R)DFTVSAMHGDMDQK(E) + C |
|  |  | 14.87 | 7.55 | [87.7](file:///E:\millscripts\viewfeed.pl%3fviewer=viewMaster.jar&side=spectrumWin&spectrumFiles=msdataSM\140307-00027-DP\14188\cpick_in\14188-Pos-aMSMS-30_47.4882.4886.0.pkl&mstagHits=VDWLTEK&cycle=1&fixedMods=carbamidomethylation&varMods=) | 13.04 | (R)AILPCIK(G) |
|  |  | 14.76 | 14.76 | 78.1 | 12.43 | (K)VVMALGDYMGASCHACIGGTNVR(A) |
|  |  | 14.54 | 7.55 | [73.1](file:///E:\millscripts\viewfeed.pl%3fviewer=viewMaster.jar&side=spectrumWin&spectrumFiles=msdataSM\140307-00027-DP\14188\cpick_in\14188-Pos-aMSMS-30_47.4826.4829.0.pkl&mstagHits=KEELTLEGIK&cycle=1&fixedMods=carbamidomethylation&varMods=) | 12.80 | (K)VVMALGDYMGASCHACIGGTNVR(A) |
|  |  | 13.85 | 7.66 | [72](file:///E:\millscripts\viewfeed.pl%3fviewer=viewMaster.jar&side=spectrumWin&spectrumFiles=msdataSM\140307-00027-DP\14188\cpick_in\14188-Pos-aMSMS-30_47.6036.6040.0.pkl&mstagHits=DQIYDIFQK&cycle=1&fixedMods=carbamidomethylation&varMods=).0 | 16.55 | (K)VDWLTEK(M) |
|  |  | 13.81 | 13.81 | [74.1](file:///E:\millscripts\viewfeed.pl%3fviewer=viewMaster.jar&side=spectrumWin&spectrumFiles=msdataSM\140307-00027-DP\14188\cpick_in\14188-Pos-aMSMS-30_47.6035.6041.0.pkl&mstagHits=VVMALGDYMGASCHACIGGTNVR&cycle=1&fixedMods=carbamidomethylation&varMods=) | 16.55 | (K)MFVLDEADEMLSR(G) + C |
|  |  | 13.68 | 13.68 | [73](file:///E:\millscripts\viewfeed.pl%3fviewer=viewMaster.jar&side=spectrumWin&spectrumFiles=msdataSM\140307-00027-DP\14188\cpick_in\14188-Pos-aMSMS-30_47.4565.4568.0.pkl&mstagHits=GVAINMVTEEDKR&cycle=1&fixedMods=carbamidomethylation&varMods=).0 | 11.92 | (K)LQMEAPHIIVGTPGR(V) |
|  |  | 13.17 | 13.17 | [66.1](file:///E:\millscripts\viewfeed.pl%3fviewer=viewMaster.jar&side=spectrumWin&spectrumFiles=msdataSM\140307-00027-DP\14188\cpick_in\14188-Pos-aMSMS-30_47.4728.4745.0.pkl&mstagHits=DFTVSAMHGDMDQK&cycle=1&fixedMods=carbamidomethylation&varMods=) | 12.45 | (K)LQAEAPHIVVGTPGR(V) |
|  |  | 13.13 | 13.13 | [68.5](file:///E:\millscripts\viewfeed.pl%3fviewer=viewMaster.jar&side=spectrumWin&spectrumFiles=msdataSM\140307-00027-DP\14188\cpick_in\14188-Pos-aMSMS-30_47.4252.4255.0.pkl&mstagHits=KGVAINMVTEEDKR&cycle=1&fixedMods=carbamidomethylation&varMods=) | 10.77 | (K)KEELTLEGIR(Q) |
|  |  | 12.97 | 12.97 | [71.8](file:///E:\millscripts\viewfeed.pl%3fviewer=viewMaster.jar&side=spectrumWin&spectrumFiles=msdataSM\140307-00027-DP\14188\cpick_in\14188-Pos-aMSMS-30_47.4736.4740.0.pkl&mstagHits=KGVAINFVTEEDKR&cycle=1&fixedMods=carbamidomethylation&varMods=) | 12.49 | (K)KEELTLEGIK(Q) |
|  |  | 12.92 | 12.92 | [74](file:///E:\millscripts\viewfeed.pl%3fviewer=viewMaster.jar&side=spectrumWin&spectrumFiles=msdataSM\140307-00027-DP\14188\cpick_in\14188-Pos-aMSMS-30_47.4863.4868.0.pkl&mstagHits=LQAEAPHIVVGTPGR&cycle=1&fixedMods=carbamidomethylation&varMods=).0 | 12.95 | (K)GYDVIAQAQSGTGK(T) |
|  |  | 12.09 | 12.09 | [64.5](file:///E:\millscripts\viewfeed.pl%3fviewer=viewMaster.jar&side=spectrumWin&spectrumFiles=msdataSM\140307-00027-DP\14188\cpick_in\14188-Pos-aMSMS-30_47.4569.4618.0.pkl&mstagHits=GVAINMVTEEDKR&cycle=1&fixedMods=carbamidomethylation&varMods=) | 11.94 | (K)GVAINMVTEEDKR(T) |
|  |  | 12.00 | 12.00 | [72.4](file:///E:\millscripts\viewfeed.pl%3fviewer=viewMaster.jar&side=spectrumWin&spectrumFiles=msdataSM\140307-00027-DP\14188\cpick_in\14188-Pos-aMSMS-30_47.3869.3902.0.pkl&mstagHits=GVAINFVTEEDKR&cycle=1&fixedMods=carbamidomethylation&varMods=) | 9.71 | (K)GVAINMVTEEDKR(T) |
|  |  | 11.37 | 11.37 | [74.7](file:///E:\millscripts\viewfeed.pl%3fviewer=viewMaster.jar&side=spectrumWin&spectrumFiles=msdataSM\140307-00027-DP\14188\cpick_in\14188-Pos-aMSMS-30_47.5089.5089.2.pkl&mstagHits=VFDMLNR&cycle=1&fixedMods=carbamidomethylation&varMods=) | 13.66 | (K)GVAINFVTEEDKR(I) |
|  |  | 11.23 | 11.23 | [70](file:///E:\millscripts\viewfeed.pl%3fviewer=viewMaster.jar&side=spectrumWin&spectrumFiles=msdataSM\140307-00027-DP\14188\cpick_in\14188-Pos-aMSMS-30_47.4832.4835.0.pkl&mstagHits=ETQALVLAPTR&cycle=1&fixedMods=carbamidomethylation&varMods=).0 | 12.82 | (K)FMRDPIR(I) |
|  |  | 11.04 | 5.65 | [78](file:///E:\millscripts\viewfeed.pl%3fviewer=viewMaster.jar&side=spectrumWin&spectrumFiles=msdataSM\140307-00027-DP\14188\cpick_in\14188-Pos-aMSMS-30_47.6043.6047.0.pkl&mstagHits=VLITTDLLAR&cycle=1&fixedMods=carbamidomethylation&varMods=).0 | 16.58 | (K)ETQALVLAPTR(E) |
|  |  | 10.13 | 10.13 | [66.6](file:///E:\millscripts\viewfeed.pl%3fviewer=viewMaster.jar&side=spectrumWin&spectrumFiles=msdataSM\140307-00027-DP\14188\cpick_in\14188-Pos-aMSMS-30_47.5294.5294.0.pkl&mstagHits=EELTLEGIR&cycle=1&fixedMods=carbamidomethylation&varMods=) | 14.41 | (K)EELTLEGIR(Q) |
|  |  | 8.84 | 4.86 | [69.5](file:///E:\millscripts\viewfeed.pl%3fviewer=viewMaster.jar&side=spectrumWin&spectrumFiles=msdataSM\140307-00027-DP\14188\cpick_in\14188-Pos-aMSMS-30_47.3963.3966.0.pkl&mstagHits=FMRDPIR&cycle=1&fixedMods=carbamidomethylation&varMods=) | 9.98 | (K)EELTLEGIR(Q) |
|  |  | 8.44 | 8.44 | [61.6](file:///E:\millscripts\viewfeed.pl%3fviewer=viewMaster.jar&side=spectrumWin&spectrumFiles=msdataSM\140307-00027-DP\14188\cpick_in\14188-Pos-aMSMS-30_47.5291.5291.0.pkl&mstagHits=EELTLEGIR&cycle=1&fixedMods=carbamidomethylation&varMods=) | 14.39 | (K)DQIYDIFQK(L) |
|  |  | 8.32 | 8.32 | [63.9](file:///E:\millscripts\viewfeed.pl%3fviewer=viewMaster.jar&side=spectrumWin&spectrumFiles=msdataSM\140307-00027-DP\14188\cpick_in\14188-Pos-aMSMS-30_47.4274.4274.0.pkl&mstagHits=KGVAINMVTEEDKR&cycle=1&fixedMods=carbamidomethylation&varMods=) | 10.85 | (K)ATQALVLAPTR(E) |
|  |  |  |  |  |  |  |
| 5 | Proliferation-associated protein 2G4 | 23.81 | 23.81 | [92.9](file:///E:\millscripts\viewfeed.pl%3fviewer=viewMaster.jar&side=spectrumWin&spectrumFiles=msdataSM\140307-00027-DP\16925\cpick_in\16925-Pos-aMSMS-30_47.6885.6923.0.pkl&mstagHits=SLVEASSSGVSVLSLCEK&cycle=1&fixedMods=carbamidomethylation&varMods=) | 16.85 | (R)SLVEASSSGVSVLSLCEK(G) + C |
|  |  | 21.44 | 21.44 | [88.2](file:///E:\millscripts\viewfeed.pl%3fviewer=viewMaster.jar&side=spectrumWin&spectrumFiles=msdataSM\140307-00027-DP\16925\cpick_in\16925-Pos-aMSMS-30_47.6284.6305.0.pkl&mstagHits=ITSGPFEPDLYK&cycle=1&fixedMods=carbamidomethylation&varMods=) | 15.19 | (R)ITSGPFEPDLYK(S) |
|  |  | 17.79 | 17.79 | [86.4](file:///E:\millscripts\viewfeed.pl%3fviewer=viewMaster.jar&side=spectrumWin&spectrumFiles=msdataSM\140307-00027-DP\16925\cpick_in\16925-Pos-aMSMS-30_47.7553.7553.0.pkl&mstagHits=HELLQPFNVLYEK&cycle=1&fixedMods=carbamidomethylation&varMods=) | 18.54 | (K)HELLQPFNVLYEK(E) |
|  |  | 15.97 | 11.27 | [90.8](file:///E:\millscripts\viewfeed.pl%3fviewer=viewMaster.jar&side=spectrumWin&spectrumFiles=msdataSM\140307-00027-DP\16925\cpick_in\16925-Pos-aMSMS-30_47.7550.7556.0.pkl&mstagHits=HELLQPFNVLYEK&cycle=1&fixedMods=carbamidomethylation&varMods=) | 18.49 | (K)HELLQPFNVLYEK(E) |
|  |  | 14.28 | 14.28 | [78](file:///E:\millscripts\viewfeed.pl%3fviewer=viewMaster.jar&side=spectrumWin&spectrumFiles=msdataSM\140307-00027-DP\16925\cpick_in\16925-Pos-aMSMS-30_47.7405.7414.0.pkl&mstagHits=FDAMPFTLR&cycle=1&fixedMods=carbamidomethylation&varMods=).0 | 18.16 | (R)FDAMPFTLR(A) |
|  |  |  |  |  |  |  |
| 6 | Inosinicase | 21.66 | 21.66 | [95.2](file:///E:\millscripts\viewfeed.pl%3fviewer=viewMaster.jar&side=spectrumWin&spectrumFiles=msdataSM\140210-00020-DP\18665\cpick_in\18665-Pos-aMSMS-30_47.6781.6826.2.pkl&mstagHits=NLTALGLNLVASGGTAK&cycle=1&fixedMods=carbamidomethylation&varMods=) | 18.49 | (R)NLTALGLNLVASGGTAK(A) |
|  |  | 21.05 | 15.49 | [97.1](file:///E:\millscripts\viewfeed.pl%3fviewer=viewMaster.jar&side=spectrumWin&spectrumFiles=msdataSM\140210-00020-DP\18665\cpick_in\18665-Pos-aMSMS-30_47.6979.7004.0.pkl&mstagHits=DVSELTGFPEMLGGR&cycle=1&fixedMods=carbamidomethylation&varMods=) | 19.22 | (R)DVSELTGFPEMLGGR(V) |
|  |  | 21.01 | 12.48 | [98](file:///E:\millscripts\viewfeed.pl%3fviewer=viewMaster.jar&side=spectrumWin&spectrumFiles=msdataSM\140210-00020-DP\18665\cpick_in\18665-Pos-aMSMS-30_47.7398.7438.3.pkl&mstagHits=ALFEEVPELLTEAEKK&cycle=1&fixedMods=carbamidomethylation&varMods=).0 | 20.50 | (K)ALFEEVPELLTEAEKK(E) |
|  |  | 20.18 | 9.37 | [93.7](file:///E:\millscripts\viewfeed.pl%3fviewer=viewMaster.jar&side=spectrumWin&spectrumFiles=msdataSM\140210-00020-DP\18665\cpick_in\18665-Pos-aMSMS-30_47.6297.6300.0.pkl&mstagHits=VVACNLYPFVK&cycle=1&fixedMods=carbamidomethylation&varMods=) | 16.54 | (R)VVACNLYPFVK(T) + C |
|  |  | 17.76 | 10.81 | [89.8](file:///E:\millscripts\viewfeed.pl%3fviewer=viewMaster.jar&side=spectrumWin&spectrumFiles=msdataSM\140210-00020-DP\18665\cpick_in\18665-Pos-aMSMS-30_47.6023.6027.0.pkl&mstagHits=EALGIPAAASFK&cycle=1&fixedMods=carbamidomethylation&varMods=) | 15.61 | (K)EALGIPAAASFK(H) |
|  |  | 17.54 | 9.24 | [87.2](file:///E:\millscripts\viewfeed.pl%3fviewer=viewMaster.jar&side=spectrumWin&spectrumFiles=msdataSM\140210-00020-DP\18665\cpick_in\18665-Pos-aMSMS-30_47.5198.5201.0.pkl&mstagHits=TLTPISAAYAR&cycle=1&fixedMods=carbamidomethylation&varMods=) | 13.12 | (K)TLTPISAAYAR(A) |
|  |  | 17.29 | 17.29 | [81.3](file:///E:\millscripts\viewfeed.pl%3fviewer=viewMaster.jar&side=spectrumWin&spectrumFiles=msdataSM\140210-00020-DP\18665\cpick_in\18665-Pos-aMSMS-30_47.6337.6403.0.pkl&mstagHits=AFTHTAQYDEAISDYFR&cycle=1&fixedMods=carbamidomethylation&varMods=) | 16.70 | (K)AFTHTAQYDEAISDYFR(K) |
|  |  | 16.92 | 16.92 | [84.6](file:///E:\millscripts\viewfeed.pl%3fviewer=viewMaster.jar&side=spectrumWin&spectrumFiles=msdataSM\140210-00020-DP\18665\cpick_in\18665-Pos-aMSMS-30_47.7184.7196.0.pkl&mstagHits=LTEVSISSDAFFPFRDNVDR&cycle=1&fixedMods=carbamidomethylation&varMods=) | 19.77 | (K)LTEVSISSDAFFPFRDNVDR  (A) |
|  |  | 16.71 | 16.71 | [76.6](file:///E:\millscripts\viewfeed.pl%3fviewer=viewMaster.jar&side=spectrumWin&spectrumFiles=msdataSM\140210-00020-DP\18665\cpick_in\18665-Pos-aMSMS-30_47.5898.5902.0.pkl&mstagHits=VCMVYDLYK&cycle=1&fixedMods=carbamidomethylation&varMods=) | 15.19 | (K)VCMVYDLYK(T) + C |
|  |  | 16.36 | 10.57 | [77.5](file:///E:\millscripts\viewfeed.pl%3fviewer=viewMaster.jar&side=spectrumWin&spectrumFiles=msdataSM\140210-00020-DP\18665\cpick_in\18665-Pos-aMSMS-30_47.5970.5973.0.pkl&mstagHits=TLFGLHLSQK&cycle=1&fixedMods=carbamidomethylation&varMods=) | 15.44 | (R)TLFGLHLSQK(R) |
|  |  | 15.88 | 15.88 | [89.7](file:///E:\millscripts\viewfeed.pl%3fviewer=viewMaster.jar&side=spectrumWin&spectrumFiles=msdataSM\140210-00020-DP\18665\cpick_in\18665-Pos-aMSMS-30_47.5383.5407.0.pkl&mstagHits=HVSPAGAAVGIPLSEDEAK&cycle=1&fixedMods=carbamidomethylation&varMods=) | 13.60 | (K)HVSPAGAAVGIPLSEDEAK  (V) |
|  |  | 15.58 | 9.56 | [88.6](file:///E:\millscripts\viewfeed.pl%3fviewer=viewMaster.jar&side=spectrumWin&spectrumFiles=msdataSM\140210-00020-DP\18665\cpick_in\18665-Pos-aMSMS-30_47.6360.6387.2.pkl&mstagHits=LDFNLIR&cycle=1&fixedMods=carbamidomethylation&varMods=) | 16.80 | (R)LDFNLIR(V) |
|  |  | 14.46 | 10.64 | [82.2](file:///E:\millscripts\viewfeed.pl%3fviewer=viewMaster.jar&side=spectrumWin&spectrumFiles=msdataSM\140210-00020-DP\18665\cpick_in\18665-Pos-aMSMS-30_47.7675.7689.0.pkl&mstagHits=ALFEEVPELLTEAEK&cycle=1&fixedMods=carbamidomethylation&varMods=) | 21.35 | (K)ALFEEVPELLTEAEK(K) |
|  |  | 14.24 | 14.24 | [77.9](file:///E:\millscripts\viewfeed.pl%3fviewer=viewMaster.jar&side=spectrumWin&spectrumFiles=msdataSM\140210-00020-DP\18665\cpick_in\18665-Pos-aMSMS-30_47.4572.4576.0.pkl&mstagHits=SGVAYIAAPSGSAADK&cycle=1&fixedMods=carbamidomethylation&varMods=) | 11.37 | (R)SGVAYIAAPSGSAADK(V) |
|  |  | 13.59 | 13.59 | [70.1](file:///E:\millscripts\viewfeed.pl%3fviewer=viewMaster.jar&side=spectrumWin&spectrumFiles=msdataSM\140210-00020-DP\18665\cpick_in\18665-Pos-aMSMS-30_47.7678.7678.0.pkl&mstagHits=ALFEEVPELLTEAEK&cycle=1&fixedMods=carbamidomethylation&varMods=) | 21.36 | (K)ALFEEVPELLTEAEK(K) |
|  |  | 13.23 | 13.23 | [78.6](file:///E:\millscripts\viewfeed.pl%3fviewer=viewMaster.jar&side=spectrumWin&spectrumFiles=msdataSM\140210-00020-DP\18665\cpick_in\18665-Pos-aMSMS-30_47.7719.7733.0.pkl&mstagHits=VVIEACDELGIILAHTNLR&cycle=1&fixedMods=carbamidomethylation&varMods=) | 21.48 | (K)VVIEACDELGIILAHTNLR(L) + C |
|  |  | 12.75 | 12.75 | [71.4](file:///E:\millscripts\viewfeed.pl%3fviewer=viewMaster.jar&side=spectrumWin&spectrumFiles=msdataSM\140210-00020-DP\18665\cpick_in\18665-Pos-aMSMS-30_47.6138.6142.0.pkl&mstagHits=ELKEALGIPAAASFK&cycle=1&fixedMods=carbamidomethylation&varMods=) | 16.00 | (K)ELKEALGIPAAASFK(H) |
|  |  | 12.26 | 12.26 | [62.9](file:///E:\millscripts\viewfeed.pl%3fviewer=viewMaster.jar&side=spectrumWin&spectrumFiles=msdataSM\140210-00020-DP\18665\cpick_in\18665-Pos-aMSMS-30_47.3162.3334.0.pkl&mstagHits=YTQSNSVCYAK&cycle=1&fixedMods=carbamidomethylation&varMods=) | 7.76 | (K)YTQSNSVCYAK(N) + C |
|  |  | 12.22 | 4.57 | [84.1](file:///E:\millscripts\viewfeed.pl%3fviewer=viewMaster.jar&side=spectrumWin&spectrumFiles=msdataSM\140210-00020-DP\18665\cpick_in\18665-Pos-aMSMS-30_47.3327.3330.0.pkl&mstagHits=DAGLAVR&cycle=1&fixedMods=carbamidomethylation&varMods=) | 8.13 | (R)DAGLAVR(D) |
|  |  | 12.19 | 9.06 | [75.1](file:///E:\millscripts\viewfeed.pl%3fviewer=viewMaster.jar&side=spectrumWin&spectrumFiles=msdataSM\140210-00020-DP\18665\cpick_in\18665-Pos-aMSMS-30_47.7643.7649.0.pkl&mstagHits=EVSDGIIAPGYEEEALTILSK&cycle=1&fixedMods=carbamidomethylation&varMods=) | 21.24 | (R)EVSDGIIAPGYEEEALTILSK  (K) |
|  |  | 12.09 | 8.91 | [73.5](file:///E:\millscripts\viewfeed.pl%3fviewer=viewMaster.jar&side=spectrumWin&spectrumFiles=msdataSM\140210-00020-DP\18665\cpick_in\18665-Pos-aMSMS-30_47.3721.3740.0.pkl&mstagHits=NIPEDNADMAR&cycle=1&fixedMods=carbamidomethylation&varMods=) | 9.09 | (R)NIPEDNADMAR(L) |
|  |  | 10.78 | 10.78 | [75.5](file:///E:\millscripts\viewfeed.pl%3fviewer=viewMaster.jar&side=spectrumWin&spectrumFiles=msdataSM\140210-00020-DP\18665\cpick_in\18665-Pos-aMSMS-30_47.6340.6340.0.pkl&mstagHits=AFTHTAQYDEAISDYFR&cycle=1&fixedMods=carbamidomethylation&varMods=) | 16.71 | (K)AFTHTAQYDEAISDYFR(K) |
|  |  | 10.54 | 4.94 | [70.5](file:///E:\millscripts\viewfeed.pl%3fviewer=viewMaster.jar&side=spectrumWin&spectrumFiles=msdataSM\140210-00020-DP\18665\cpick_in\18665-Pos-aMSMS-30_47.5604.5604.0.pkl&mstagHits=SLFSNVVTK&cycle=1&fixedMods=carbamidomethylation&varMods=) | 14.29 | (K)SLFSNVVTK(N) |
|  |  | 10.49 | 10.49 | [65.1](file:///E:\millscripts\viewfeed.pl%3fviewer=viewMaster.jar&side=spectrumWin&spectrumFiles=msdataSM\140210-00020-DP\18665\cpick_in\18665-Pos-aMSMS-30_47.7646.7646.0.pkl&mstagHits=EVSDGIIAPGYEEEALTILSK&cycle=1&fixedMods=carbamidomethylation&varMods=) | 21.26 | (R)EVSDGIIAPGYEEEALTILSK  (K) |
|  |  | 9.79 | 9.79 | [73.2](file:///E:\millscripts\viewfeed.pl%3fviewer=viewMaster.jar&side=spectrumWin&spectrumFiles=msdataSM\140210-00020-DP\18665\cpick_in\18665-Pos-aMSMS-30_47.5194.5197.0.pkl&mstagHits=TGLVEFAR&cycle=1&fixedMods=carbamidomethylation&varMods=) | 13.10 | (K)TGLVEFAR(N) |
|  |  | 16.71 | 16.71 | [76.6](file:///E:\millscripts\viewfeed.pl%3fviewer=viewMaster.jar&side=spectrumWin&spectrumFiles=msdataSM\140210-00020-DP\18665\cpick_in\18665-Pos-aMSMS-30_47.5898.5902.0.pkl&mstagHits=VCMVYDLYK&cycle=1&fixedMods=carbamidomethylation&varMods=) | 15.19 | (K)VCMVYDLYK(T) + C |
|  |  | 16.36 | 10.57 | [77.5](file:///E:\millscripts\viewfeed.pl%3fviewer=viewMaster.jar&side=spectrumWin&spectrumFiles=msdataSM\140210-00020-DP\18665\cpick_in\18665-Pos-aMSMS-30_47.5970.5973.0.pkl&mstagHits=TLFGLHLSQK&cycle=1&fixedMods=carbamidomethylation&varMods=) | 15.44 | (R)TLFGLHLSQK(R) |
|  |  | 15.88 | 15.88 | [89.7](file:///E:\millscripts\viewfeed.pl%3fviewer=viewMaster.jar&side=spectrumWin&spectrumFiles=msdataSM\140210-00020-DP\18665\cpick_in\18665-Pos-aMSMS-30_47.5383.5407.0.pkl&mstagHits=HVSPAGAAVGIPLSEDEAK&cycle=1&fixedMods=carbamidomethylation&varMods=) | 13.60 | (K)HVSPAGAAVGIPLSEDEAK  (V) |
|  |  | 15.58 | 9.56 | [88.6](file:///E:\millscripts\viewfeed.pl%3fviewer=viewMaster.jar&side=spectrumWin&spectrumFiles=msdataSM\140210-00020-DP\18665\cpick_in\18665-Pos-aMSMS-30_47.6360.6387.2.pkl&mstagHits=LDFNLIR&cycle=1&fixedMods=carbamidomethylation&varMods=) | 16.80 | (R)LDFNLIR(V) |
| 7 | Alanine aminotransferase | 18.96 | 18.96 | [81.7](file:///E:\millscripts\viewfeed.pl%3fviewer=viewMaster.jar&side=spectrumWin&spectrumFiles=msdataSM\140210-00020-DP\5951\cpick_in\5951-Pos-aMSMS-30_47.3174.3191.0.pkl&mstagHits=EGTYHFR&cycle=1&fixedMods=carbamidomethylation&varMods=) | 7.73 | (R)EGTYHFR(M) |
|  | 2 | 17.20 | 6.80 | [85](file:///E:\millscripts\viewfeed.pl%3fviewer=viewMaster.jar&side=spectrumWin&spectrumFiles=msdataSM\140210-00020-DP\5951\cpick_in\5951-Pos-aMSMS-30_47.5429.5432.0.pkl&mstagHits=AGEIELELQR&cycle=1&fixedMods=carbamidomethylation&varMods=).0 | 13.67 | (K)AGEIELELQR(G) |
|  |  | 16.75 | 16.75 | [81.9](file:///E:\millscripts\viewfeed.pl%3fviewer=viewMaster.jar&side=spectrumWin&spectrumFiles=msdataSM\140210-00020-DP\5951\cpick_in\5951-Pos-aMSMS-30_47.5843.5855.0.pkl&mstagHits=VLCIINPGNPTGQVQSR&cycle=1&fixedMods=carbamidomethylation&varMods=) | 14.88 | (K)VLCIINPGNPTGQVQSR(K)  + C |
|  |  | 13.59 | 5.91 | [87](file:///E:\millscripts\viewfeed.pl%3fviewer=viewMaster.jar&side=spectrumWin&spectrumFiles=msdataSM\140210-00020-DP\5951\cpick_in\5951-Pos-aMSMS-30_47.2990.2993.0.pkl&mstagHits=ILVSGGGK&cycle=1&fixedMods=carbamidomethylation&varMods=).0 | 7.30 | (K)ILVSGGGK(S) |
|  |  | 12.73 | 12.73 | [72.5](file:///E:\millscripts\viewfeed.pl%3fviewer=viewMaster.jar&side=spectrumWin&spectrumFiles=msdataSM\140210-00020-DP\5951\cpick_in\5951-Pos-aMSMS-30_47.6047.6072.0.pkl&mstagHits=ILTLESMNPQVK&cycle=1&fixedMods=carbamidomethylation&varMods=) | 15.64 | (R)ILTLESMNPQVK(A) |
|  |  | 11.60 | 1.58 | [80.7](file:///E:\millscripts\viewfeed.pl%3fviewer=viewMaster.jar&side=spectrumWin&spectrumFiles=msdataSM\140210-00020-DP\5951\cpick_in\5951-Pos-aMSMS-30_47.4581.4584.2.pkl&mstagHits=IFIPAK&cycle=1&fixedMods=carbamidomethylation&varMods=) | 11.35 | (R)IFIPAK(A) |
|  |  | 10.89 | 10.89 | [76.6](file:///E:\millscripts\viewfeed.pl%3fviewer=viewMaster.jar&side=spectrumWin&spectrumFiles=msdataSM\140210-00020-DP\5951\cpick_in\5951-Pos-aMSMS-30_47.5692.5811.0.pkl&mstagHits=MTILPPVEK&cycle=1&fixedMods=carbamidomethylation&varMods=) | 14.44 | (R)MTILPPVEK(L) |
|  |  | 10.78 | 5.35 | [77.3](file:///E:\millscripts\viewfeed.pl%3fviewer=viewMaster.jar&side=spectrumWin&spectrumFiles=msdataSM\140210-00020-DP\5951\cpick_in\5951-Pos-aMSMS-30_47.4363.4366.0.pkl&mstagHits=KPFTEVIR&cycle=1&fixedMods=carbamidomethylation&varMods=) | 10.75 | (K)KPFTEVIR(A) |
|  |  | 10.48 | 10.48 | [72.6](file:///E:\millscripts\viewfeed.pl%3fviewer=viewMaster.jar&side=spectrumWin&spectrumFiles=msdataSM\140210-00020-DP\5951\cpick_in\5951-Pos-aMSMS-30_47.4588.4593.0.pkl&mstagHits=EKESVLGNLAK&cycle=1&fixedMods=carbamidomethylation&varMods=) | 11.38 | (R)EKESVLGNLAK(K) |
|  |  | 10.23 | 3.30 | [81.9](file:///E:\millscripts\viewfeed.pl%3fviewer=viewMaster.jar&side=spectrumWin&spectrumFiles=msdataSM\140210-00020-DP\5951\cpick_in\5951-Pos-aMSMS-30_47.3470.3470.0.pkl&mstagHits=AVEYAVR&cycle=1&fixedMods=carbamidomethylation&varMods=) | 8.44 | (K)AVEYAVR(G) |
|  |  | 10.06 | 3.47 | [78.8](file:///E:\millscripts\viewfeed.pl%3fviewer=viewMaster.jar&side=spectrumWin&spectrumFiles=msdataSM\140210-00020-DP\5951\cpick_in\5951-Pos-aMSMS-30_47.4127.4130.0.pkl&mstagHits=GPIVLK&cycle=1&fixedMods=carbamidomethylation&varMods=) | 10.12 | (R)GPIVLK(A) |
|  |  |  |  |  |  |  |
| 8 | ATP synthase subunit | 14.91 | 14.91 | [81.5](file:///E:\millscripts\viewfeed.pl%3fviewer=viewMaster.jar&side=spectrumWin&spectrumFiles=msdataSM\140307-00027-DP\2575\cpick_in\2575-Pos-aMSMS-30_47.8371.8371.0.pkl&mstagHits=VALTGLTVAEYFR&cycle=1&fixedMods=carbamidomethylation&varMods=) | 20.41 | (R)VALTGLTVAEYFR(D) |
|  | beta | 8.62 | 8.62 | [64.3](file:///E:\millscripts\viewfeed.pl%3fviewer=viewMaster.jar&side=spectrumWin&spectrumFiles=msdataSM\140307-00027-DP\2575\cpick_in\2575-Pos-aMSMS-30_47.7972.7972.0.pkl&mstagHits=AIAELGIYPAVDPLDSTSR&cycle=1&fixedMods=carbamidomethylation&varMods=) | 19.53 | (R)AIAELGIYPAVDPLDSTSR(I) |
|  |  |  |  |  |  |  |
| 9 | leucineaminopeptidase 3 | 21.38 | 16.83 | [94.9](file:///E:\millscripts\viewfeed.pl%3fviewer=viewMaster.jar&side=spectrumWin&spectrumFiles=msdataSM\140307-00027-DP\5438\cpick_in\5438-Pos-aMSMS-30_47.5673.5676.0.pkl&mstagHits=GVLFASGQNLAR&cycle=1&fixedMods=carbamidomethylation&varMods=) | 14.08 | (K)GVLFASGQNLAR(Q) |
|  |  | 17.16 | 17.16 | [85.9](file:///E:\millscripts\viewfeed.pl%3fviewer=viewMaster.jar&side=spectrumWin&spectrumFiles=msdataSM\140307-00027-DP\5438\cpick_in\5438-Pos-aMSMS-30_47.6308.6337.0.pkl&mstagHits=GSPNANEPPLVFVGK&cycle=1&fixedMods=carbamidomethylation&varMods=) | 15.70 | (K)GSPNANEPPLVFVGK(G) |
|  |  | 15.31 | 15.31 | [85.5](file:///E:\millscripts\viewfeed.pl%3fviewer=viewMaster.jar&side=spectrumWin&spectrumFiles=msdataSM\140307-00027-DP\5438\cpick_in\5438-Pos-aMSMS-30_47.5160.5164.0.pkl&mstagHits=LFEASIETGDR&cycle=1&fixedMods=carbamidomethylation&varMods=) | 12.69 | (K)LFEASIETGDR(V) |
|  |  | 15.07 | 9.07 | [82.5](file:///E:\millscripts\viewfeed.pl%3fviewer=viewMaster.jar&side=spectrumWin&spectrumFiles=msdataSM\140307-00027-DP\5438\cpick_in\5438-Pos-aMSMS-30_47.6182.6216.0.pkl&mstagHits=GLVLGIYSK&cycle=1&fixedMods=carbamidomethylation&varMods=) | 15.32 | (K)GLVLGIYSK(E) |
|  |  | 14.05 | 14.05 | [76.7](file:///E:\millscripts\viewfeed.pl%3fviewer=viewMaster.jar&side=spectrumWin&spectrumFiles=msdataSM\140307-00027-DP\5438\cpick_in\5438-Pos-aMSMS-30_47.6319.6319.0.pkl&mstagHits=GSPNANEPPLVFVGK&cycle=1&fixedMods=carbamidomethylation&varMods=) | 15.73 | (K)GSPNANEPPLVFVGK(G) |
|  |  | 13.56 | 13.56 | [74.3](file:///E:\millscripts\viewfeed.pl%3fviewer=viewMaster.jar&side=spectrumWin&spectrumFiles=msdataSM\140307-00027-DP\5438\cpick_in\5438-Pos-aMSMS-30_47.5666.5669.0.pkl&mstagHits=ETLNISGPPLK&cycle=1&fixedMods=carbamidomethylation&varMods=) | 14.03 | (R)ETLNISGPPLK(A) |
|  |  | 11.05 | 11.05 | [61.8](file:///E:\millscripts\viewfeed.pl%3fviewer=viewMaster.jar&side=spectrumWin&spectrumFiles=msdataSM\140307-00027-DP\5438\cpick_in\5438-Pos-aMSMS-30_47.6778.6778.0.pkl&mstagHits=GSDEPPVFLEIHYK&cycle=1&fixedMods=carbamidomethylation&varMods=) | 17.07 | (K)GSDEPPVFLEIHYK(G) |
|  |  | 10.97 | 10.97 | [61.1](file:///E:\millscripts\viewfeed.pl%3fviewer=viewMaster.jar&side=spectrumWin&spectrumFiles=msdataSM\140307-00027-DP\5438\cpick_in\5438-Pos-aMSMS-30_47.6743.6743.0.pkl&mstagHits=ADMGGAATICSAIVSAAK&cycle=1&fixedMods=carbamidomethylation&varMods=) | 16.95 | (R)ADMGGAATICSAIVSAAK(L) + C |
|  |  | 8.85 | 8.85 | [63.5](file:///E:\millscripts\viewfeed.pl%3fviewer=viewMaster.jar&side=spectrumWin&spectrumFiles=msdataSM\140307-00027-DP\5438\cpick_in\5438-Pos-aMSMS-30_47.4378.4378.0.pkl&mstagHits=LYGSGDQEAWQK&cycle=1&fixedMods=carbamidomethylation&varMods=) | 10.66 | (K)LYGSGDQEAWQK(G) |
|  |  | 8.80 | 8.80 | [62.1](file:///E:\millscripts\viewfeed.pl%3fviewer=viewMaster.jar&side=spectrumWin&spectrumFiles=msdataSM\140307-00027-DP\5438\cpick_in\5438-Pos-aMSMS-30_47.5420.5420.0.pkl&mstagHits=SAGACTAAAFLK&cycle=1&fixedMods=carbamidomethylation&varMods=) | 13.37 | (R)SAGACTAAAFLK(E) + C |
|  |  | 8.79 | 0.80 | [68.9](file:///E:\millscripts\viewfeed.pl%3fviewer=viewMaster.jar&side=spectrumWin&spectrumFiles=msdataSM\140307-00027-DP\5438\cpick_in\5438-Pos-aMSMS-30_47.8492.8492.0.pkl&mstagHits=TLIEFLLR&cycle=1&fixedMods=carbamidomethylation&varMods=) | 21.16 | (R)TLIEFLLR(F) |
|  |  | 7.75 | 7.75 | [62.3](file:///E:\millscripts\viewfeed.pl%3fviewer=viewMaster.jar&side=spectrumWin&spectrumFiles=msdataSM\140307-00027-DP\5438\cpick_in\5438-Pos-aMSMS-30_47.7778.7778.0.pkl&mstagHits=LILADALCYAHTFNPK&cycle=1&fixedMods=carbamidomethylation&varMods=) | 19.43 | (R)LILADALCYAHTFNPK(V) + C |
|  |  |  |  |  |  |  |
| 10 | 26S protease regulatory | 14.99 | 14.99 | [73.3](file:///E:\millscripts\viewfeed.pl%3fviewer=viewMaster.jar&side=spectrumWin&spectrumFiles=msdataSM\140307-00027-DP\7864\cpick_in\7864-Pos-aMSMS-30_47.5506.5506.0.pkl&mstagHits=GVCTEAGMYALR&cycle=1&fixedMods=carbamidomethylation&varMods=) | 13.39 | (K)GVCTEAGMYALR(E) + C |
|  | subunit 8 | 10.30 | 10.30 | [75.3](file:///E:\millscripts\viewfeed.pl%3fviewer=viewMaster.jar&side=spectrumWin&spectrumFiles=msdataSM\140307-00027-DP\7864\cpick_in\7864-Pos-aMSMS-30_47.3380.3383.0.pkl&mstagHits=VSGSELVQK&cycle=1&fixedMods=carbamidomethylation&varMods=) | 8.29 | (R)VSGSELVQK(F) |
|  |  | 10.11 | 10.11 | [71.9](file:///E:\millscripts\viewfeed.pl%3fviewer=viewMaster.jar&side=spectrumWin&spectrumFiles=msdataSM\140307-00027-DP\7864\cpick_in\7864-Pos-aMSMS-30_47.5780.5780.0.pkl&mstagHits=GVLLYGPPGTGK&cycle=1&fixedMods=carbamidomethylation&varMods=) | 14.03 | (K)GVLLYGPPGTGK(T) |
|  |  | 10.08 | 10.08 | [73.5](file:///E:\millscripts\viewfeed.pl%3fviewer=viewMaster.jar&side=spectrumWin&spectrumFiles=msdataSM\140307-00027-DP\7864\cpick_in\7864-Pos-aMSMS-30_47.5979.5982.0.pkl&mstagHits=ELFVMAR&cycle=1&fixedMods=carbamidomethylation&varMods=) | 14.58 | (R)ELFVMAR(E) |
|  |  | 9.74 | 9.74 | [72](file:///E:\millscripts\viewfeed.pl%3fviewer=viewMaster.jar&side=spectrumWin&spectrumFiles=msdataSM\140307-00027-DP\7864\cpick_in\7864-Pos-aMSMS-30_47.5509.5514.0.pkl&mstagHits=IAELMPGASGAEVK&cycle=1&fixedMods=carbamidomethylation&varMods=).0 | 13.45 | (K)IAELMPGASGAEVK(G) |
|  |  |  |  |  |  |  |
| 11 | Glucose-6-phosphate 1- | 17.17 | 17.17 | [76.9](file:///E:\millscripts\viewfeed.pl%3fviewer=viewMaster.jar&side=spectrumWin&spectrumFiles=msdataSM\140307-00027-DP\19382\cpick_in\19382-Pos-aMSMS-30_47.3483.3486.0.pkl&mstagHits=VQPNEAVYTK&cycle=1&fixedMods=carbamidomethylation&varMods=) | 8.53 | (R)VQPNEAVYTK(M) |
|  | dehydrogenase | 15.32 | 15.32 | [74.5](file:///E:\millscripts\viewfeed.pl%3fviewer=viewMaster.jar&side=spectrumWin&spectrumFiles=msdataSM\140307-00027-DP\19382\cpick_in\19382-Pos-aMSMS-30_47.4804.4816.0.pkl&mstagHits=VGFQYEGTYK&cycle=1&fixedMods=carbamidomethylation&varMods=) | 12.12 | (R)VGFQYEGTYK(W) |
|  |  | 14.83 | 14.83 | [71.6](file:///E:\millscripts\viewfeed.pl%3fviewer=viewMaster.jar&side=spectrumWin&spectrumFiles=msdataSM\140307-00027-DP\19382\cpick_in\19382-Pos-aMSMS-30_47.3359.3364.0.pkl&mstagHits=EPFGTEGR&cycle=1&fixedMods=carbamidomethylation&varMods=) | 8.23 | (K)EPFGTEGR(G) |
|  |  | 14.58 | 14.58 | [85.1](file:///E:\millscripts\viewfeed.pl%3fviewer=viewMaster.jar&side=spectrumWin&spectrumFiles=msdataSM\140307-00027-DP\19382\cpick_in\19382-Pos-aMSMS-30_47.4902.4915.0.pkl&mstagHits=GYLDDPTVPR&cycle=1&fixedMods=carbamidomethylation&varMods=) | 12.38 | (K)GYLDDPTVPR(G) |
|  |  | 13.91 | 13.91 | [74.3](file:///E:\millscripts\viewfeed.pl%3fviewer=viewMaster.jar&side=spectrumWin&spectrumFiles=msdataSM\140307-00027-DP\19382\cpick_in\19382-Pos-aMSMS-30_47.4392.4419.0.pkl&mstagHits=GPTEADELMK&cycle=1&fixedMods=carbamidomethylation&varMods=) | 10.88 | (R)GPTEADELMK(R) |
|  |  | 12.98 | 12.98 | [82.2](file:///E:\millscripts\viewfeed.pl%3fviewer=viewMaster.jar&side=spectrumWin&spectrumFiles=msdataSM\140307-00027-DP\19382\cpick_in\19382-Pos-aMSMS-30_47.4446.4458.0.pkl&mstagHits=NSYVAGQYDDAASYQR&cycle=1&fixedMods=carbamidomethylation&varMods=) | 11.05 | (R)NSYVAGQYDDAASYQR(L) |
|  |  | 12.22 | 12.22 | [75.8](file:///E:\millscripts\viewfeed.pl%3fviewer=viewMaster.jar&side=spectrumWin&spectrumFiles=msdataSM\140307-00027-DP\19382\cpick_in\19382-Pos-aMSMS-30_47.6713.6716.0.pkl&mstagHits=IFGPIWNR&cycle=1&fixedMods=carbamidomethylation&varMods=) | 16.80 | (R)IFGPIWNR(D) |
|  |  | 11.48 | 6.49 | [74.5](file:///E:\millscripts\viewfeed.pl%3fviewer=viewMaster.jar&side=spectrumWin&spectrumFiles=msdataSM\140307-00027-DP\19382\cpick_in\19382-Pos-aMSMS-30_47.4790.4798.0.pkl&mstagHits=IIVEKPFGR&cycle=1&fixedMods=carbamidomethylation&varMods=) | 12.06 | (R)IIVEKPFGR(D) |
|  |  | 11.22 | 11.22 | [68.5](file:///E:\millscripts\viewfeed.pl%3fviewer=viewMaster.jar&side=spectrumWin&spectrumFiles=msdataSM\140307-00027-DP\19382\cpick_in\19382-Pos-aMSMS-30_47.4784.4789.0.pkl&mstagHits=IIVEKPFGR&cycle=1&fixedMods=carbamidomethylation&varMods=) | 12.03 | (R)IIVEKPFGR(D) |
|  |  | 10.61 | 10.61 | [64.1](file:///E:\millscripts\viewfeed.pl%3fviewer=viewMaster.jar&side=spectrumWin&spectrumFiles=msdataSM\140307-00027-DP\19382\cpick_in\19382-Pos-aMSMS-30_47.4682.4685.0.pkl&mstagHits=LTVADIR&cycle=1&fixedMods=carbamidomethylation&varMods=) | 11.78 | (R)LTVADIR(K) |
|  |  | 10.46 | 10.46 | [66.6](file:///E:\millscripts\viewfeed.pl%3fviewer=viewMaster.jar&side=spectrumWin&spectrumFiles=msdataSM\140307-00027-DP\19382\cpick_in\19382-Pos-aMSMS-30_47.4582.4585.0.pkl&mstagHits=TQVCGILR&cycle=1&fixedMods=carbamidomethylation&varMods=) | 11.44 | (R)TQVCGILR(E) + C |
|  |  | 10.26 | 10.26 | [63.7](file:///E:\millscripts\viewfeed.pl%3fviewer=viewMaster.jar&side=spectrumWin&spectrumFiles=msdataSM\140307-00027-DP\19382\cpick_in\19382-Pos-aMSMS-30_47.4807.4807.0.pkl&mstagHits=VGFQYEGTYK&cycle=1&fixedMods=carbamidomethylation&varMods=) | 12.14 | (R)VGFQYEGTYK(W) |
|  |  | 8.71 | 8.71 | [64](file:///E:\millscripts\viewfeed.pl%3fviewer=viewMaster.jar&side=spectrumWin&spectrumFiles=msdataSM\140307-00027-DP\19382\cpick_in\19382-Pos-aMSMS-30_47.4449.4449.0.pkl&mstagHits=NSYVAGQYDDAASYQR&cycle=1&fixedMods=carbamidomethylation&varMods=).0 | 11.06 | (R)NSYVAGQYDDAASYQR(L) |
|  |  | 8.41 | 8.41 | [61.4](file:///E:\millscripts\viewfeed.pl%3fviewer=viewMaster.jar&side=spectrumWin&spectrumFiles=msdataSM\140307-00027-DP\19382\cpick_in\19382-Pos-aMSMS-30_47.3576.3579.0.pkl&mstagHits=LPDAYER&cycle=1&fixedMods=carbamidomethylation&varMods=) | 8.77 | (K)LPDAYER(L) |
|  |  |  |  |  |  |  |
| 12 | Protein disulfide- | 25.30 | 25.30 | [97.2](file:///E:\millscripts\viewfeed.pl%3fviewer=viewMaster.jar&side=spectrumWin&spectrumFiles=msdataSM\140307-00027-DP\19613\cpick_in\19613-Pos-aMSMS-30_47.6385.6449.2.pkl&mstagHits=LAAVDATVNQVLASR&cycle=1&fixedMods=carbamidomethylation&varMods=) | 16.70 | (K)LAAVDATVNQVLASR(Y) |
|  | isomerase A6 | 23.15 | 17.42 | [96.9](file:///E:\millscripts\viewfeed.pl%3fviewer=viewMaster.jar&side=spectrumWin&spectrumFiles=msdataSM\140307-00027-DP\19613\cpick_in\19613-Pos-aMSMS-30_47.5584.5588.0.pkl&mstagHits=KDVIELTDDSFDK&cycle=1&fixedMods=carbamidomethylation&varMods=) | 14.28 | (K)KDVIELTDDSFDK(N) |
|  |  | 22.65 | 22.65 | [92.3](file:///E:\millscripts\viewfeed.pl%3fviewer=viewMaster.jar&side=spectrumWin&spectrumFiles=msdataSM\140307-00027-DP\19613\cpick_in\19613-Pos-aMSMS-30_47.6040.6120.2.pkl&mstagHits=GSTAPVGGGAFPTIVER&cycle=1&fixedMods=carbamidomethylation&varMods=) | 15.50 | (R)GSTAPVGGGAFPTIVER(E) |
|  |  | 21.16 | 13.28 | [94.6](file:///E:\millscripts\viewfeed.pl%3fviewer=viewMaster.jar&side=spectrumWin&spectrumFiles=msdataSM\140307-00027-DP\19613\cpick_in\19613-Pos-aMSMS-30_47.7895.7954.2.pkl&mstagHits=TGEAIVDAALSALR&cycle=1&fixedMods=carbamidomethylation&varMods=) | 20.76 | (R)TGEAIVDAALSALR(Q) |
|  |  | 20.41 | 20.41 | [90.7](file:///E:\millscripts\viewfeed.pl%3fviewer=viewMaster.jar&side=spectrumWin&spectrumFiles=msdataSM\140307-00027-DP\19613\cpick_in\19613-Pos-aMSMS-30_47.6542.6624.2.pkl&mstagHits=GSFSEQGINEFLR&cycle=1&fixedMods=carbamidomethylation&varMods=) | 17.21 | (K)GSFSEQGINEFLR(E) |
|  |  | 19.79 | 19.79 | [94](file:///E:\millscripts\viewfeed.pl%3fviewer=viewMaster.jar&side=spectrumWin&spectrumFiles=msdataSM\140307-00027-DP\19613\cpick_in\19613-Pos-aMSMS-30_47.3321.3484.0.pkl&mstagHits=GESPVDYDGGR&cycle=1&fixedMods=carbamidomethylation&varMods=) | 8.24 | (K)GESPVDYDGGR(T) |
|  |  | 17.63 | 17.63 | [92](file:///E:\millscripts\viewfeed.pl%3fviewer=viewMaster.jar&side=spectrumWin&spectrumFiles=msdataSM\140307-00027-DP\19613\cpick_in\19613-Pos-aMSMS-30_47.3466.3506.2.pkl&mstagHits=GESPVDYDGGR&cycle=1&fixedMods=carbamidomethylation&varMods=) | 8.62 | (K)GESPVDYDGGR(T) |
|  |  | 16.97 | 11.89 | [91](file:///E:\millscripts\viewfeed.pl%3fviewer=viewMaster.jar&side=spectrumWin&spectrumFiles=msdataSM\140307-00027-DP\19613\cpick_in\19613-Pos-aMSMS-30_47.6098.6142.2.pkl&mstagHits=NLEPEWAAAASEVK&cycle=1&fixedMods=carbamidomethylation&varMods=) | 15.75 | (K)NLEPEWAAAASEVK(E) |
|  |  | 15.71 | 15.71 | [80.2](file:///E:\millscripts\viewfeed.pl%3fviewer=viewMaster.jar&side=spectrumWin&spectrumFiles=msdataSM\140307-00027-DP\19613\cpick_in\19613-Pos-aMSMS-30_47.4159.4192.2.pkl&mstagHits=LTPEWK&cycle=1&fixedMods=carbamidomethylation&varMods=) | 10.54 | (R)LTPEWK(K) |
|  |  | 15.22 | 15.22 | [88.1](file:///E:\millscripts\viewfeed.pl%3fviewer=viewMaster.jar&side=spectrumWin&spectrumFiles=msdataSM\140307-00027-DP\19613\cpick_in\19613-Pos-aMSMS-30_47.6421.6482.0.pkl&mstagHits=LYSSSDDVIELTPSNFNR&cycle=1&fixedMods=carbamidomethylation&varMods=) | 16.84 | (-)LYSSSDDVIELTPSNFNR(E) |
|  |  | 14.84 | 14.84 | [83.4](file:///E:\millscripts\viewfeed.pl%3fviewer=viewMaster.jar&side=spectrumWin&spectrumFiles=msdataSM\140307-00027-DP\19613\cpick_in\19613-Pos-aMSMS-30_47.5589.5592.0.pkl&mstagHits=KDVIELTDDSFDK&cycle=1&fixedMods=carbamidomethylation&varMods=) | 14.30 | (K)KDVIELTDDSFDK(N) |
|  |  | 14.18 | 14.18 | [75.4](file:///E:\millscripts\viewfeed.pl%3fviewer=viewMaster.jar&side=spectrumWin&spectrumFiles=msdataSM\140307-00027-DP\19613\cpick_in\19613-Pos-aMSMS-30_47.6033.6036.0.pkl&mstagHits=DVIELTDDSFDK&cycle=1&fixedMods=carbamidomethylation&varMods=) | 15.47 | (K)DVIELTDDSFDK(N) |
|  |  | 13.62 | 1.61 | [90.5](file:///E:\millscripts\viewfeed.pl%3fviewer=viewMaster.jar&side=spectrumWin&spectrumFiles=msdataSM\140307-00027-DP\19613\cpick_in\19613-Pos-aMSMS-30_47.4497.4522.2.pkl&mstagHits=GFPTIK&cycle=1&fixedMods=carbamidomethylation&varMods=) | 11.48 | (R)GFPTIK(I) |
|  |  | 13.26 | 13.26 | [75.4](file:///E:\millscripts\viewfeed.pl%3fviewer=viewMaster.jar&side=spectrumWin&spectrumFiles=msdataSM\140307-00027-DP\19613\cpick_in\19613-Pos-aMSMS-30_47.3418.3460.2.pkl&mstagHits=EPWDGR&cycle=1&fixedMods=carbamidomethylation&varMods=) | 8.47 | (R)EPWDGR(D) |
|  |  | 13.25 | 13.25 | [81.3](file:///E:\millscripts\viewfeed.pl%3fviewer=viewMaster.jar&side=spectrumWin&spectrumFiles=msdataSM\140307-00027-DP\19613\cpick_in\19613-Pos-aMSMS-30_47.2220.2223.0.pkl&mstagHits=VGAVDADK&cycle=1&fixedMods=carbamidomethylation&varMods=) | 5.48 | (K)VGAVDADK(H) |
|  |  | 11.62 | 5.74 | [68.2](file:///E:\millscripts\viewfeed.pl%3fviewer=viewMaster.jar&side=spectrumWin&spectrumFiles=msdataSM\140307-00027-DP\19613\cpick_in\19613-Pos-aMSMS-30_47.3929.3932.0.pkl&mstagHits=AATALKDVVK&cycle=1&fixedMods=carbamidomethylation&varMods=) | 9.86 | (K)AATALKDVVK(V) |
|  |  | 10.94 | 0.00 | [78.2](file:///E:\millscripts\viewfeed.pl%3fviewer=viewMaster.jar&side=spectrumWin&spectrumFiles=msdataSM\140307-00027-DP\19613\cpick_in\19613-Pos-aMSMS-30_47.2968.2971.0.pkl&mstagHits=IFGSNK&cycle=1&fixedMods=carbamidomethylation&varMods=) | 7.20 | (K)IFGSNK(N) |
|  |  | 10.83 | 6.14 | [68.9](file:///E:\millscripts\viewfeed.pl%3fviewer=viewMaster.jar&side=spectrumWin&spectrumFiles=msdataSM\140307-00027-DP\19613\cpick_in\19613-Pos-aMSMS-30_47.6942.6945.2.pkl&mstagHits=NSYLEVLLK&cycle=1&fixedMods=carbamidomethylation&varMods=) | 18.39 | (R)NSYLEVLLK(L) |
|  |  | 10.68 | 3.36 | [66.6](file:///E:\millscripts\viewfeed.pl%3fviewer=viewMaster.jar&side=spectrumWin&spectrumFiles=msdataSM\140307-00027-DP\19613\cpick_in\19613-Pos-aMSMS-30_47.4114.4134.2.pkl&mstagHits=ELSFGR&cycle=1&fixedMods=carbamidomethylation&varMods=) | 10.37 | (R)ELSFGR(G) |
|  |  | 10.04 | 10.04 | [76.7](file:///E:\millscripts\viewfeed.pl%3fviewer=viewMaster.jar&side=spectrumWin&spectrumFiles=msdataSM\140307-00027-DP\19613\cpick_in\19613-Pos-aMSMS-30_47.2165.2165.0.pkl&mstagHits=NRPEDYQGGR&cycle=1&fixedMods=carbamidomethylation&varMods=) | 5.24 | (K)NRPEDYQGGR(T) |
|  |  | 7.73 | 7.73 | [65.1](file:///E:\millscripts\viewfeed.pl%3fviewer=viewMaster.jar&side=spectrumWin&spectrumFiles=msdataSM\140307-00027-DP\19613\cpick_in\19613-Pos-aMSMS-30_47.6648.6648.0.pkl&mstagHits=VKLAAVDATVNQVLASR&cycle=1&fixedMods=carbamidomethylation&varMods=) | 17.58 | (R)VKLAAVDATVNQVLASR(Y) |
|  |  |  |  |  |  |  |
| 13 | Elongation factor 2 | 19.91 | 19.91 | [88.6](file:///E:\millscripts\viewfeed.pl%3fviewer=viewMaster.jar&side=spectrumWin&spectrumFiles=msdataSM\140307-00027-DP\20108\cpick_in\20108-Pos-aMSMS-30_47.5311.5314.0.pkl&mstagHits=YEWDVAEAR&cycle=1&fixedMods=carbamidomethylation&varMods=) | 13.15 | (K)YEWDVAEAR(K) |
|  |  | 17.68 | 17.68 | [85.3](file:///E:\millscripts\viewfeed.pl%3fviewer=viewMaster.jar&side=spectrumWin&spectrumFiles=msdataSM\140307-00027-DP\20108\cpick_in\20108-Pos-aMSMS-30_47.3723.3726.0.pkl&mstagHits=EGALCEENMR&cycle=1&fixedMods=carbamidomethylation&varMods=) | 9.09 | (K)EGALCEENMR(G) + C |
|  |  | 17.68 | 12.63 | [82.8](file:///E:\millscripts\viewfeed.pl%3fviewer=viewMaster.jar&side=spectrumWin&spectrumFiles=msdataSM\140307-00027-DP\20108\cpick_in\20108-Pos-aMSMS-30_47.6021.6033.0.pkl&mstagHits=CLYASVLTAQPR&cycle=1&fixedMods=carbamidomethylation&varMods=) | 14.92 | (R)CLYASVLTAQPR(L) + C |
|  |  | 16.84 | 16.84 | [82.9](file:///E:\millscripts\viewfeed.pl%3fviewer=viewMaster.jar&side=spectrumWin&spectrumFiles=msdataSM\140307-00027-DP\20108\cpick_in\20108-Pos-aMSMS-30_47.5563.5571.0.pkl&mstagHits=ETVSEESNVLCLSK&cycle=1&fixedMods=carbamidomethylation&varMods=) | 13.73 | (R)ETVSEESNVLCLSK(S) + C |
|  |  | 15.77 | 6.10 | [82.6](file:///E:\millscripts\viewfeed.pl%3fviewer=viewMaster.jar&side=spectrumWin&spectrumFiles=msdataSM\140307-00027-DP\20108\cpick_in\20108-Pos-aMSMS-30_47.7154.7154.0.pkl&mstagHits=VFDAIMNFK&cycle=1&fixedMods=carbamidomethylation&varMods=) | 17.63 | (K)VFDAIMNFK(K) |
|  |  | 15.38 | 15.38 | [67.6](file:///E:\millscripts\viewfeed.pl%3fviewer=viewMaster.jar&side=spectrumWin&spectrumFiles=msdataSM\140307-00027-DP\20108\cpick_in\20108-Pos-aMSMS-30_47.5132.5135.0.pkl&mstagHits=QFAEMYVAK&cycle=1&fixedMods=carbamidomethylation&varMods=) | 12.68 | (K)QFAEMYVAK(F) |
|  |  | 13.99 | 13.99 | [82.8](file:///E:\millscripts\viewfeed.pl%3fviewer=viewMaster.jar&side=spectrumWin&spectrumFiles=msdataSM\140307-00027-DP\20108\cpick_in\20108-Pos-aMSMS-30_47.5307.5310.0.pkl&mstagHits=GVQYLNEIK&cycle=1&fixedMods=carbamidomethylation&varMods=) | 13.09 | (K)GVQYLNEIK(D) |
|  |  | 13.68 | 13.68 | [82.4](file:///E:\millscripts\viewfeed.pl%3fviewer=viewMaster.jar&side=spectrumWin&spectrumFiles=msdataSM\140307-00027-DP\20108\cpick_in\20108-Pos-aMSMS-30_47.5139.5142.0.pkl&mstagHits=EDLYLKPIQR&cycle=1&fixedMods=carbamidomethylation&varMods=) | 12.71 | (K)EDLYLKPIQR(T) |
|  |  | 12.62 | 12.62 | [71.9](file:///E:\millscripts\viewfeed.pl%3fviewer=viewMaster.jar&side=spectrumWin&spectrumFiles=msdataSM\140307-00027-DP\20108\cpick_in\20108-Pos-aMSMS-30_47.4949.4952.0.pkl&mstagHits=STLTDSLVCK&cycle=1&fixedMods=carbamidomethylation&varMods=) | 12.18 | (K)STLTDSLVCK(A) + C |
|  |  | 12.17 | 12.17 | [74.9](file:///E:\millscripts\viewfeed.pl%3fviewer=viewMaster.jar&side=spectrumWin&spectrumFiles=msdataSM\140307-00027-DP\20108\cpick_in\20108-Pos-aMSMS-30_47.5061.5064.0.pkl&mstagHits=IKPVLMMNK&cycle=1&fixedMods=carbamidomethylation&varMods=) | 12.47 | (R)IKPVLMMNK(M) |
|  |  | 12.13 | 4.68 | [76.8](file:///E:\millscripts\viewfeed.pl%3fviewer=viewMaster.jar&side=spectrumWin&spectrumFiles=msdataSM\140307-00027-DP\20108\cpick_in\20108-Pos-aMSMS-30_47.6278.6289.0.pkl&mstagHits=GPLMMYISK&cycle=1&fixedMods=carbamidomethylation&varMods=) | 15.54 | (K)GPLMMYISK(M) |
|  |  | 12.02 | 12.02 | [79](file:///E:\millscripts\viewfeed.pl%3fviewer=viewMaster.jar&side=spectrumWin&spectrumFiles=msdataSM\140307-00027-DP\20108\cpick_in\20108-Pos-aMSMS-30_47.6116.6119.0.pkl&mstagHits=VFSGLVSTGLK&cycle=1&fixedMods=carbamidomethylation&varMods=) | 15.15 | (R)VFSGLVSTGLK(V) |
|  |  | 11.13 | 11.13 | [77.6](file:///E:\millscripts\viewfeed.pl%3fviewer=viewMaster.jar&side=spectrumWin&spectrumFiles=msdataSM\140307-00027-DP\20108\cpick_in\20108-Pos-aMSMS-30_47.5163.5166.0.pkl&mstagHits=FSVSPVVR&cycle=1&fixedMods=carbamidomethylation&varMods=) | 12.78 | (K)FSVSPVVR(V) |
|  |  | 10.22 | 10.22 | [71.8](file:///E:\millscripts\viewfeed.pl%3fviewer=viewMaster.jar&side=spectrumWin&spectrumFiles=msdataSM\140307-00027-DP\20108\cpick_in\20108-Pos-aMSMS-30_47.3773.3781.0.pkl&mstagHits=SDPVVSYR&cycle=1&fixedMods=carbamidomethylation&varMods=) | 9.25 | (K)SDPVVSYR(E) |
|  |  | 9.61 | 2.95 | [75.6](file:///E:\millscripts\viewfeed.pl%3fviewer=viewMaster.jar&side=spectrumWin&spectrumFiles=msdataSM\140307-00027-DP\20108\cpick_in\20108-Pos-aMSMS-30_47.5325.5328.0.pkl&mstagHits=TILMMGR&cycle=1&fixedMods=carbamidomethylation&varMods=) | 13.21 | (R)TILMMGR(Y) |
|  |  | 8.83 | 8.83 | [65.3](file:///E:\millscripts\viewfeed.pl%3fviewer=viewMaster.jar&side=spectrumWin&spectrumFiles=msdataSM\140307-00027-DP\20108\cpick_in\20108-Pos-aMSMS-30_47.4260.4263.0.pkl&mstagHits=GGGQIIPTAR&cycle=1&fixedMods=carbamidomethylation&varMods=) | 10.43 | (R)GGGQIIPTAR(R) |
|  |  | 7.34 | 3.08 | [64.5](file:///E:\millscripts\viewfeed.pl%3fviewer=viewMaster.jar&side=spectrumWin&spectrumFiles=msdataSM\140307-00027-DP\20108\cpick_in\20108-Pos-aMSMS-30_47.4454.4463.0.pkl&mstagHits=DLEEDHACIPIKK&cycle=1&fixedMods=carbamidomethylation&varMods=) | 10.94 | (K)DLEEDHACIPIKK(S) + C |
|  |  |  |  |  |  |  |
| 14 | Protein disulfide- | 23.54 | 23.54 | [96.8](file:///E:\millscripts\viewfeed.pl%3fviewer=viewMaster.jar&side=spectrumWin&spectrumFiles=msdataSM\140307-00027-DP\5455\cpick_in\5455-Pos-aMSMS-30_47.6236.6252.0.pkl&mstagHits=VVVAENFDEIVNNENK&cycle=1&fixedMods=carbamidomethylation&varMods=) | 15.86 | (K)VVVAENFDEIVNNENK(D) |
|  | isomerase A3 | 22.04 | 22.04 | [97.1](file:///E:\millscripts\viewfeed.pl%3fviewer=viewMaster.jar&side=spectrumWin&spectrumFiles=msdataSM\140307-00027-DP\5455\cpick_in\5455-Pos-aMSMS-30_47.3960.3975.2.pkl&mstagHits=LAPEYEAAATR&cycle=1&fixedMods=carbamidomethylation&varMods=) | 9.83 | (R)LAPEYEAAATR(L) |
|  |  | 21.63 | 15.59 | [97.5](file:///E:\millscripts\viewfeed.pl%3fviewer=viewMaster.jar&side=spectrumWin&spectrumFiles=msdataSM\140307-00027-DP\5455\cpick_in\5455-Pos-aMSMS-30_47.4203.4203.2.pkl&mstagHits=TADGIVSHLK&cycle=1&fixedMods=carbamidomethylation&varMods=) | 10.56 | (R)TADGIVSHLK(K) |
|  |  | 21.29 | 21.29 | [90.4](file:///E:\millscripts\viewfeed.pl%3fviewer=viewMaster.jar&side=spectrumWin&spectrumFiles=msdataSM\140307-00027-DP\5455\cpick_in\5455-Pos-aMSMS-30_47.6239.6243.0.pkl&mstagHits=VVVAENFDEIVNNENK&cycle=1&fixedMods=carbamidomethylation&varMods=) | 15.87 | (K)VVVAENFDEIVNNENK(D) |
|  |  | 18.85 | 11.23 | [85.1](file:///E:\millscripts\viewfeed.pl%3fviewer=viewMaster.jar&side=spectrumWin&spectrumFiles=msdataSM\140307-00027-DP\5455\cpick_in\5455-Pos-aMSMS-30_47.5330.5353.0.pkl&mstagHits=FVMQEEFSR&cycle=1&fixedMods=carbamidomethylation&varMods=) | 13.57 | (K)FVMQEEFSR(D) |
|  |  | 18.80 | 18.80 | [91.1](file:///E:\millscripts\viewfeed.pl%3fviewer=viewMaster.jar&side=spectrumWin&spectrumFiles=msdataSM\140307-00027-DP\5455\cpick_in\5455-Pos-aMSMS-30_47.6932.6935.0.pkl&mstagHits=GFPTIYFSPANK&cycle=1&fixedMods=carbamidomethylation&varMods=) | 17.53 | (R)GFPTIYFSPANK(K) |
|  |  | 18.31 | 18.31 | [89.7](file:///E:\millscripts\viewfeed.pl%3fviewer=viewMaster.jar&side=spectrumWin&spectrumFiles=msdataSM\140307-00027-DP\5455\cpick_in\5455-Pos-aMSMS-30_47.4934.4986.2.pkl&mstagHits=YGVSGYPTLK&cycle=1&fixedMods=carbamidomethylation&varMods=) | 12.55 | (K)YGVSGYPTLK(I) |
|  |  | 18.05 | 18.05 | [89.7](file:///E:\millscripts\viewfeed.pl%3fviewer=viewMaster.jar&side=spectrumWin&spectrumFiles=msdataSM\140307-00027-DP\5455\cpick_in\5455-Pos-aMSMS-30_47.4277.4280.0.pkl&mstagHits=LSKDPNIVIAK&cycle=1&fixedMods=carbamidomethylation&varMods=) | 10.75 | (K)LSKDPNIVIAK(M) |
|  |  | 17.39 | 17.39 | [84.3](file:///E:\millscripts\viewfeed.pl%3fviewer=viewMaster.jar&side=spectrumWin&spectrumFiles=msdataSM\140307-00027-DP\5455\cpick_in\5455-Pos-aMSMS-30_47.6213.6216.0.pkl&mstagHits=SDVLELTDDNFESR&cycle=1&fixedMods=carbamidomethylation&varMods=) | 15.79 | (-)SDVLELTDDNFESR(I) |
|  |  | 16.73 | 16.73 | [85.6](file:///E:\millscripts\viewfeed.pl%3fviewer=viewMaster.jar&side=spectrumWin&spectrumFiles=msdataSM\140307-00027-DP\5455\cpick_in\5455-Pos-aMSMS-30_47.4127.4130.2.pkl&mstagHits=QAGPASVPLR&cycle=1&fixedMods=carbamidomethylation&varMods=) | 10.30 | (K)QAGPASVPLR(T) |
|  |  | 15.49 | 15.49 | [75.6](file:///E:\millscripts\viewfeed.pl%3fviewer=viewMaster.jar&side=spectrumWin&spectrumFiles=msdataSM\140307-00027-DP\5455\cpick_in\5455-Pos-aMSMS-30_47.6687.6756.0.pkl&mstagHits=FLQDYFDGNLK&cycle=1&fixedMods=carbamidomethylation&varMods=) | 16.97 | (R)FLQDYFDGNLK(R) |
|  |  | 15.17 | 15.17 | [85.2](file:///E:\millscripts\viewfeed.pl%3fviewer=viewMaster.jar&side=spectrumWin&spectrumFiles=msdataSM\140307-00027-DP\5455\cpick_in\5455-Pos-aMSMS-30_47.4794.4823.2.pkl&mstagHits=LNFAVASR&cycle=1&fixedMods=carbamidomethylation&varMods=) | 12.14 | (K)LNFAVASR(K) |
|  |  | 15.08 | 4.27 | [75.5](file:///E:\millscripts\viewfeed.pl%3fviewer=viewMaster.jar&side=spectrumWin&spectrumFiles=msdataSM\140307-00027-DP\5455\cpick_in\5455-Pos-aMSMS-30_47.2372.2375.0.pkl&mstagHits=YKELGEK&cycle=1&fixedMods=carbamidomethylation&varMods=) | 5.82 | (K)YKELGEK(L) |
|  |  | 14.84 | 14.84 | [80.3](file:///E:\millscripts\viewfeed.pl%3fviewer=viewMaster.jar&side=spectrumWin&spectrumFiles=msdataSM\140307-00027-DP\5455\cpick_in\5455-Pos-aMSMS-30_47.3201.3204.0.pkl&mstagHits=DGEEAGAYDGPR&cycle=1&fixedMods=carbamidomethylation&varMods=) | 7.90 | (R)DGEEAGAYDGPR(T) |
|  |  | 14.84 | 9.44 | [79.4](file:///E:\millscripts\viewfeed.pl%3fviewer=viewMaster.jar&side=spectrumWin&spectrumFiles=msdataSM\140307-00027-DP\5455\cpick_in\5455-Pos-aMSMS-30_47.3805.3810.0.pkl&mstagHits=EATNPPVIQEEKPK&cycle=1&fixedMods=carbamidomethylation&varMods=) | 9.39 | (R)EATNPPVIQEEKPK(K) |
|  |  | 14.40 | 5.77 | [80.5](file:///E:\millscripts\viewfeed.pl%3fviewer=viewMaster.jar&side=spectrumWin&spectrumFiles=msdataSM\140307-00027-DP\5455\cpick_in\5455-Pos-aMSMS-30_47.3806.3811.0.pkl&mstagHits=EATNPPVIQEEKPK&cycle=1&fixedMods=carbamidomethylation&varMods=) | 9.39 | (R)EATNPPVIQEEKPK(K) |
|  |  | 12.32 | 12.32 | [60.5](file:///E:\millscripts\viewfeed.pl%3fviewer=viewMaster.jar&side=spectrumWin&spectrumFiles=msdataSM\140307-00027-DP\5455\cpick_in\5455-Pos-aMSMS-30_47.5136.5136.0.pkl&mstagHits=MDATANDVPSPYEVR&cycle=1&fixedMods=carbamidomethylation&varMods=) | 13.10 | (K)MDATANDVPSPYEVR(G) |
|  |  | 12.31 | 12.31 | [77.2](file:///E:\millscripts\viewfeed.pl%3fviewer=viewMaster.jar&side=spectrumWin&spectrumFiles=msdataSM\140307-00027-DP\5455\cpick_in\5455-Pos-aMSMS-30_47.3985.4133.0.pkl&mstagHits=SEPIPESNDGPVK&cycle=1&fixedMods=carbamidomethylation&varMods=) | 9.92 | (K)SEPIPESNDGPVK(V) |
|  |  | 11.21 | 11.21 | [65.1](file:///E:\millscripts\viewfeed.pl%3fviewer=viewMaster.jar&side=spectrumWin&spectrumFiles=msdataSM\140307-00027-DP\5455\cpick_in\5455-Pos-aMSMS-30_47.4149.4149.0.pkl&mstagHits=TADGIVSHLK&cycle=1&fixedMods=carbamidomethylation&varMods=) | 10.39 | (R)TADGIVSHLK(K) |
|  |  | 10.49 | 3.11 | [74](file:///E:\millscripts\viewfeed.pl%3fviewer=viewMaster.jar&side=spectrumWin&spectrumFiles=msdataSM\140307-00027-DP\5455\cpick_in\5455-Pos-aMSMS-30_47.2236.2236.0.pkl&mstagHits=TEEEFKK&cycle=1&fixedMods=carbamidomethylation&varMods=).0 | 5.42 | (R)TEEEFKK(F) |
|  |  | 10.16 | 10.16 | [60.5](file:///E:\millscripts\viewfeed.pl%3fviewer=viewMaster.jar&side=spectrumWin&spectrumFiles=msdataSM\140307-00027-DP\5455\cpick_in\5455-Pos-aMSMS-30_47.7849.7849.0.pkl&mstagHits=ELSDFISYLQR&cycle=1&fixedMods=carbamidomethylation&varMods=) | 19.68 | (R)ELSDFISYLQR(E) |
|  |  | 10.15 | 10.15 | [69.3](file:///E:\millscripts\viewfeed.pl%3fviewer=viewMaster.jar&side=spectrumWin&spectrumFiles=msdataSM\140307-00027-DP\5455\cpick_in\5455-Pos-aMSMS-30_47.4339.4349.0.pkl&mstagHits=DPNIVIAK&cycle=1&fixedMods=carbamidomethylation&varMods=) | 10.91 | (K)DPNIVIAK(M) |
|  |  | 9.86 | 9.86 | [68.9](file:///E:\millscripts\viewfeed.pl%3fviewer=viewMaster.jar&side=spectrumWin&spectrumFiles=msdataSM\140307-00027-DP\5455\cpick_in\5455-Pos-aMSMS-30_47.5041.5041.0.pkl&mstagHits=MDATANDVPSPYEVR&cycle=1&fixedMods=carbamidomethylation&varMods=) | 12.84 | (K)MDATANDVPSPYEVR(G) |
|  |  | 9.56 | -0.25 | [63.2](file:///E:\millscripts\viewfeed.pl%3fviewer=viewMaster.jar&side=spectrumWin&spectrumFiles=msdataSM\140307-00027-DP\5455\cpick_in\5455-Pos-aMSMS-30_47.4669.4672.2.pkl&mstagHits=GIVPLAK&cycle=1&fixedMods=carbamidomethylation&varMods=) | 11.79 | (K)GIVPLAK(V) |
|  |  | 9.26 | 9.26 | [69.7](file:///E:\millscripts\viewfeed.pl%3fviewer=viewMaster.jar&side=spectrumWin&spectrumFiles=msdataSM\140307-00027-DP\5455\cpick_in\5455-Pos-aMSMS-30_47.3828.3831.0.pkl&mstagHits=RLAPEYEAAATR&cycle=1&fixedMods=carbamidomethylation&varMods=) | 9.49 | (K)RLAPEYEAAATR(L) |
|  |  | 8.25 | 8.25 | [61](file:///E:\millscripts\viewfeed.pl%3fviewer=viewMaster.jar&side=spectrumWin&spectrumFiles=msdataSM\140307-00027-DP\5455\cpick_in\5455-Pos-aMSMS-30_47.5038.5038.0.pkl&mstagHits=MDATANDVPSPYEVR&cycle=1&fixedMods=carbamidomethylation&varMods=).0 | 12.83 | (K)MDATANDVPSPYEVR(G) |
|  |  | 6.65 | 1.99 | [60.8](file:///E:\millscripts\viewfeed.pl%3fviewer=viewMaster.jar&side=spectrumWin&spectrumFiles=msdataSM\140307-00027-DP\5455\cpick_in\5455-Pos-aMSMS-30_47.2600.2600.0.pkl&mstagHits=TVAYTEQK&cycle=1&fixedMods=carbamidomethylation&varMods=) | 6.34 | (K)TVAYTEQK(M) |
|  |  |  |  |  |  |  |
| 15 | ATP synthase subunit | 27.25 | 27.25 | [100](file:///E:\millscripts\viewfeed.pl%3fviewer=viewMaster.jar&side=spectrumWin&spectrumFiles=msdataSM\140307-00027-DP\8100\cpick_in\8100-Pos-aMSMS-30_47.6294.6373.0.pkl&mstagHits=LVLEVAQHLGESTVR&cycle=1&fixedMods=carbamidomethylation&varMods=) | 15.74 | (R)LVLEVAQHLGESTVR(T) |
|  | beta | 24.63 | 15.14 | [96.8](file:///E:\millscripts\viewfeed.pl%3fviewer=viewMaster.jar&side=spectrumWin&spectrumFiles=msdataSM\140307-00027-DP\8100\cpick_in\8100-Pos-aMSMS-30_47.6293.6312.3.pkl&mstagHits=LVLEVAQHLGESTVR&cycle=1&fixedMods=carbamidomethylation&varMods=) | 15.73 | (R)LVLEVAQHLGESTVR(T) |
|  |  | 23.53 | 23.53 | [96.4](file:///E:\millscripts\viewfeed.pl%3fviewer=viewMaster.jar&side=spectrumWin&spectrumFiles=msdataSM\140307-00027-DP\8100\cpick_in\8100-Pos-aMSMS-30_47.6525.6576.2.pkl&mstagHits=FTQAGSEVSALLGR&cycle=1&fixedMods=carbamidomethylation&varMods=) | 16.45 | (R)FTQAGSEVSALLGR(I) |
|  |  | 21.94 | 16.77 | [95.1](file:///E:\millscripts\viewfeed.pl%3fviewer=viewMaster.jar&side=spectrumWin&spectrumFiles=msdataSM\140307-00027-DP\8100\cpick_in\8100-Pos-aMSMS-30_47.9463.9488.2.pkl&mstagHits=TVLIMELINNVAK&cycle=1&fixedMods=carbamidomethylation&varMods=) | 24.00 | (K)TVLIMELINNVAK(A) |
|  |  | 21.71 | 21.71 | [85.9](file:///E:\millscripts\viewfeed.pl%3fviewer=viewMaster.jar&side=spectrumWin&spectrumFiles=msdataSM\140307-00027-DP\8100\cpick_in\8100-Pos-aMSMS-30_47.5591.5610.3.pkl&mstagHits=IMDPNIVGSEHYDVAR&cycle=1&fixedMods=carbamidomethylation&varMods=) | 13.77 | (R)IMDPNIVGSEHYDVAR(G) |
|  |  | 20.87 | 20.87 | [94.7](file:///E:\millscripts\viewfeed.pl%3fviewer=viewMaster.jar&side=spectrumWin&spectrumFiles=msdataSM\140307-00027-DP\8100\cpick_in\8100-Pos-aMSMS-30_47.5566.5647.0.pkl&mstagHits=VALVYGQMNEPPGAR&cycle=1&fixedMods=carbamidomethylation&varMods=) | 13.67 | (K)VALVYGQMNEPPGAR(A) |
|  |  | 20.66 | 20.66 | [88.7](file:///E:\millscripts\viewfeed.pl%3fviewer=viewMaster.jar&side=spectrumWin&spectrumFiles=msdataSM\140307-00027-DP\8100\cpick_in\8100-Pos-aMSMS-30_47.7620.7633.0.pkl&mstagHits=AIAELGIYPAVDPLDSTSR&cycle=1&fixedMods=carbamidomethylation&varMods=) | 19.45 | (R)AIAELGIYPAVDPLDSTSR(I) |
|  |  | 20.37 | 20.37 | [76.8](file:///E:\millscripts\viewfeed.pl%3fviewer=viewMaster.jar&side=spectrumWin&spectrumFiles=msdataSM\140307-00027-DP\8100\cpick_in\8100-Pos-aMSMS-30_47.5572.5641.0.pkl&mstagHits=IMDPNIVGSEHYDVAR&cycle=1&fixedMods=carbamidomethylation&varMods=) | 13.69 | (R)IMDPNIVGSEHYDVAR(G) |
|  |  | 18.87 | 10.65 | [91.9](file:///E:\millscripts\viewfeed.pl%3fviewer=viewMaster.jar&side=spectrumWin&spectrumFiles=msdataSM\140307-00027-DP\8100\cpick_in\8100-Pos-aMSMS-30_47.7600.7657.0.pkl&mstagHits=AIAELGIYPAVDPLDSTSR&cycle=1&fixedMods=carbamidomethylation&varMods=) | 19.39 | (R)AIAELGIYPAVDPLDSTSR(I) |
|  |  | 18.29 | 11.79 | [89.8](file:///E:\millscripts\viewfeed.pl%3fviewer=viewMaster.jar&side=spectrumWin&spectrumFiles=msdataSM\140307-00027-DP\8100\cpick_in\8100-Pos-aMSMS-30_47.6499.6543.0.pkl&mstagHits=IPSAVGYQPTLATDMGTMQER&cycle=1&fixedMods=carbamidomethylation&varMods=) | 16.35 | (R)IPSAVGYQPTLATDMGTMQER(I) |
|  |  | 17.86 | 13.16 | [79](file:///E:\millscripts\viewfeed.pl%3fviewer=viewMaster.jar&side=spectrumWin&spectrumFiles=msdataSM\140307-00027-DP\8100\cpick_in\8100-Pos-aMSMS-30_47.5384.5387.0.pkl&mstagHits=AHGGYSVFAGVGER&cycle=1&fixedMods=carbamidomethylation&varMods=).0 | 13.14 | (K)AHGGYSVFAGVGER(T) |
|  |  | 17.18 | 17.18 | [82.5](file:///E:\millscripts\viewfeed.pl%3fviewer=viewMaster.jar&side=spectrumWin&spectrumFiles=msdataSM\140307-00027-DP\8100\cpick_in\8100-Pos-aMSMS-30_47.5579.5583.0.pkl&mstagHits=IMDPNIVGSEHYDVAR&cycle=1&fixedMods=carbamidomethylation&varMods=) | 13.73 | (R)IMDPNIVGSEHYDVAR(G) |
|  |  | 17.14 | 17.14 | [91](file:///E:\millscripts\viewfeed.pl%3fviewer=viewMaster.jar&side=spectrumWin&spectrumFiles=msdataSM\140307-00027-DP\8100\cpick_in\8100-Pos-aMSMS-30_47.5380.5383.0.pkl&mstagHits=AHGGYSVFAGVGER&cycle=1&fixedMods=carbamidomethylation&varMods=).0 | 13.09 | (K)AHGGYSVFAGVGER(T) |
|  |  | 16.73 | 9.39 | [82.9](file:///E:\millscripts\viewfeed.pl%3fviewer=viewMaster.jar&side=spectrumWin&spectrumFiles=msdataSM\140307-00027-DP\8100\cpick_in\8100-Pos-aMSMS-30_47.6024.6027.0.pkl&mstagHits=IMNVIGEPIDER&cycle=1&fixedMods=carbamidomethylation&varMods=) | 14.96 | (R)IMNVIGEPIDER(G) |
|  |  | 16.64 | 11.95 | [83.5](file:///E:\millscripts\viewfeed.pl%3fviewer=viewMaster.jar&side=spectrumWin&spectrumFiles=msdataSM\140307-00027-DP\8100\cpick_in\8100-Pos-aMSMS-30_47.5531.5534.0.pkl&mstagHits=TIAMDGTEGLVR&cycle=1&fixedMods=carbamidomethylation&varMods=) | 13.55 | (R)TIAMDGTEGLVR(G) |
|  |  | 16.15 | 16.15 | [88.3](file:///E:\millscripts\viewfeed.pl%3fviewer=viewMaster.jar&side=spectrumWin&spectrumFiles=msdataSM\140307-00027-DP\8100\cpick_in\8100-Pos-aMSMS-30_47.6530.6534.0.pkl&mstagHits=VVDLLAPYAK&cycle=1&fixedMods=carbamidomethylation&varMods=) | 16.47 | (K)VVDLLAPYAK(G) |
|  |  | 15.83 | 15.83 | [83.8](file:///E:\millscripts\viewfeed.pl%3fviewer=viewMaster.jar&side=spectrumWin&spectrumFiles=msdataSM\140307-00027-DP\8100\cpick_in\8100-Pos-aMSMS-30_47.6085.6151.0.pkl&mstagHits=IGLFGGAGVGK&cycle=1&fixedMods=carbamidomethylation&varMods=) | 15.11 | (K)IGLFGGAGVGK(T) |
|  |  | 15.59 | 15.59 | [89.5](file:///E:\millscripts\viewfeed.pl%3fviewer=viewMaster.jar&side=spectrumWin&spectrumFiles=msdataSM\140307-00027-DP\8100\cpick_in\8100-Pos-aMSMS-30_47.7528.7587.0.pkl&mstagHits=EGNDLYHEMIESGVINLK&cycle=1&fixedMods=carbamidomethylation&varMods=) | 19.14 | (R)EGNDLYHEMIESGVINLK(D) |
|  |  | 15.41 | 15.41 | [77](file:///E:\millscripts\viewfeed.pl%3fviewer=viewMaster.jar&side=spectrumWin&spectrumFiles=msdataSM\140307-00027-DP\8100\cpick_in\8100-Pos-aMSMS-30_47.6504.6507.0.pkl&mstagHits=IPSAVGYQPTLATDMGTMQER&cycle=1&fixedMods=carbamidomethylation&varMods=).0 | 16.37 | (R)IPSAVGYQPTLATDMGTMQER(I) |
|  |  | 14.13 | 14.13 | [79.9](file:///E:\millscripts\viewfeed.pl%3fviewer=viewMaster.jar&side=spectrumWin&spectrumFiles=msdataSM\140307-00027-DP\8100\cpick_in\8100-Pos-aMSMS-30_47.7544.7544.0.pkl&mstagHits=EGNDLYHEMIESGVINLK&cycle=1&fixedMods=carbamidomethylation&varMods=) | 19.24 | (R)EGNDLYHEMIESGVINLK(D) |
|  |  | 12.81 | 12.81 | [70.3](file:///E:\millscripts\viewfeed.pl%3fviewer=viewMaster.jar&side=spectrumWin&spectrumFiles=msdataSM\140307-00027-DP\8100\cpick_in\8100-Pos-aMSMS-30_47.7536.7536.0.pkl&mstagHits=EGNDLYHEMIESGVINLK&cycle=1&fixedMods=carbamidomethylation&varMods=) | 19.21 | (R)EGNDLYHEMIESGVINLK(D) |
|  |  | 11.05 | 11.05 | [68](file:///E:\millscripts\viewfeed.pl%3fviewer=viewMaster.jar&side=spectrumWin&spectrumFiles=msdataSM\140307-00027-DP\8100\cpick_in\8100-Pos-aMSMS-30_47.7532.7532.0.pkl&mstagHits=EGNDLYHEMIESGVINLK&cycle=1&fixedMods=carbamidomethylation&varMods=).0 | 19.19 | (R)EGNDLYHEMIESGVINLK(D) |
|  |  | 9.59 | 9.59 | [63.8](file:///E:\millscripts\viewfeed.pl%3fviewer=viewMaster.jar&side=spectrumWin&spectrumFiles=msdataSM\140307-00027-DP\8100\cpick_in\8100-Pos-aMSMS-30_47.7540.7540.0.pkl&mstagHits=EGNDLYHEMIESGVINLK&cycle=1&fixedMods=carbamidomethylation&varMods=) | 19.23 | (R)EGNDLYHEMIESGVINLK(D) |
|  |  | 9.03 | 9.03 | [66.3](file:///E:\millscripts\viewfeed.pl%3fviewer=viewMaster.jar&side=spectrumWin&spectrumFiles=msdataSM\140307-00027-DP\8100\cpick_in\8100-Pos-aMSMS-30_47.7863.7990.2.pkl&mstagHits=VALTGLTVAEYFR&cycle=1&fixedMods=carbamidomethylation&varMods=) | 20.13 | (R)VALTGLTVAEYFR(D) |
|  |  | 8.67 | 8.67 | [72.7](file:///E:\millscripts\viewfeed.pl%3fviewer=viewMaster.jar&side=spectrumWin&spectrumFiles=msdataSM\140307-00027-DP\8100\cpick_in\8100-Pos-aMSMS-30_47.6850.6899.0.pkl&mstagHits=VLDSGAPIKIPVGPETLGR&cycle=1&fixedMods=carbamidomethylation&varMods=) | 17.44 | (K)VLDSGAPIKIPVGPETLGR(I) |
|  |  |  |  |  |  |  |
| 16 | Tubulin alpha-1B chain | 27.47 | 27.47 | [95.9](file:///E:\millscripts\viewfeed.pl%3fviewer=viewMaster.jar&side=spectrumWin&spectrumFiles=msdataSM\140307-00027-DP\9080\cpick_in\9080-Pos-aMSMS-30_47.6169.6220.2.pkl&mstagHits=AVCMLSNTTAVAEAWAR&cycle=1&fixedMods=carbamidomethylation&varMods=) | 18.28 | (R)AVCMLSNTTAVAEAWAR(L) + C |
|  |  | 25.91 | 25.91 | [91.4](file:///E:\millscripts\viewfeed.pl%3fviewer=viewMaster.jar&side=spectrumWin&spectrumFiles=msdataSM\140307-00027-DP\9080\cpick_in\9080-Pos-aMSMS-30_47.6481.6551.2.pkl&mstagHits=AVCMLSNTTAIAEAWAR&cycle=1&fixedMods=carbamidomethylation&varMods=) | 19.61 | (R)AVCMLSNTTAIAEAWAR(L) + C |
|  |  | 23.82 | 23.82 | [94.3](file:///E:\millscripts\viewfeed.pl%3fviewer=viewMaster.jar&side=spectrumWin&spectrumFiles=msdataSM\140307-00027-DP\9080\cpick_in\9080-Pos-aMSMS-30_47.5475.5483.0.pkl&mstagHits=QLFHPEQLITGKEDAANNYAR&cycle=1&fixedMods=carbamidomethylation&varMods=) | 15.31 | (R)QLFHPEQLITGKEDAANNYAR(G) |
|  |  | 21.76 | 12.76 | [96.4](file:///E:\millscripts\viewfeed.pl%3fviewer=viewMaster.jar&side=spectrumWin&spectrumFiles=msdataSM\140307-00027-DP\9080\cpick_in\9080-Pos-aMSMS-30_47.7571.7644.2.pkl&mstagHits=LISQIVSSITASLR&cycle=1&fixedMods=carbamidomethylation&varMods=) | 22.76 | (R)LISQIVSSITASLR(F) |
|  |  | 21.25 | 16.82 | [92.8](file:///E:\millscripts\viewfeed.pl%3fviewer=viewMaster.jar&side=spectrumWin&spectrumFiles=msdataSM\140307-00027-DP\9080\cpick_in\9080-Pos-aMSMS-30_47.6529.6612.2.pkl&mstagHits=AVFVDLEPTVIDEVR&cycle=1&fixedMods=carbamidomethylation&varMods=) | 19.82 | (R)AVFVDLEPTVIDEVR(T) |
|  |  | 21.08 | 14.65 | [85.5](file:///E:\millscripts\viewfeed.pl%3fviewer=viewMaster.jar&side=spectrumWin&spectrumFiles=msdataSM\140307-00027-DP\9080\cpick_in\9080-Pos-aMSMS-30_47.6318.6323.0.pkl&mstagHits=IHFPLATYAPVISAEK&cycle=1&fixedMods=carbamidomethylation&varMods=) | 18.92 | (R)IHFPLATYAPVISAEK(A) |
|  |  | 20.48 | 20.48 | [89.5](file:///E:\millscripts\viewfeed.pl%3fviewer=viewMaster.jar&side=spectrumWin&spectrumFiles=msdataSM\140307-00027-DP\9080\cpick_in\9080-Pos-aMSMS-30_47.6023.6064.0.pkl&mstagHits=AFVHWYVGEGMEEGEFSEAR&cycle=1&fixedMods=carbamidomethylation&varMods=) | 17.65 | (R)AFVHWYVGEGMEEGEFSEAR(E) |
|  |  | 20.47 | 20.47 | [100](file:///E:\millscripts\viewfeed.pl%3fviewer=viewMaster.jar&side=spectrumWin&spectrumFiles=msdataSM\140307-00027-DP\9080\cpick_in\9080-Pos-aMSMS-30_47.5987.6046.3.pkl&mstagHits=AYHEQLSVAEITNACFEPANQMVK&cycle=1&fixedMods=carbamidomethylation&varMods=) | 17.50 | (K)AYHEQLSVAEITNACFEPANQMVK(C) + C |
|  |  | 20.43 | 15.00 | [88.3](file:///E:\millscripts\viewfeed.pl%3fviewer=viewMaster.jar&side=spectrumWin&spectrumFiles=msdataSM\140307-00027-DP\9080\cpick_in\9080-Pos-aMSMS-30_47.6872.6904.0.pkl&mstagHits=AVFVDLEPTVIDEIR&cycle=1&fixedMods=carbamidomethylation&varMods=) | 21.03 | (R)AVFVDLEPTVIDEIR(N) |
|  |  | 20.19 | 20.19 | [94](file:///E:\millscripts\viewfeed.pl%3fviewer=viewMaster.jar&side=spectrumWin&spectrumFiles=msdataSM\140307-00027-DP\9080\cpick_in\9080-Pos-aMSMS-30_47.6442.6476.2.pkl&mstagHits=TIQFVDWCPTGFK&cycle=1&fixedMods=carbamidomethylation&varMods=).0 | 19.44 | (R)TIQFVDWCPTGFK(V) + C |
|  |  | 19.96 | 19.96 | [68.6](file:///E:\millscripts\viewfeed.pl%3fviewer=viewMaster.jar&side=spectrumWin&spectrumFiles=msdataSM\140307-00027-DP\9080\cpick_in\9080-Pos-aMSMS-30_47.5356.5431.2.pkl&mstagHits=YMACCLLYR&cycle=1&fixedMods=carbamidomethylation&varMods=) | 14.84 | (K)YMACCLLYR(G) + C |
|  |  | 19.82 | 19.82 | [95.2](file:///E:\millscripts\viewfeed.pl%3fviewer=viewMaster.jar&side=spectrumWin&spectrumFiles=msdataSM\140307-00027-DP\9080\cpick_in\9080-Pos-aMSMS-30_47.3409.3413.0.pkl&mstagHits=LSVDYGKK&cycle=1&fixedMods=carbamidomethylation&varMods=) | 8.41 | (R)LSVDYGKK(S) |
|  |  | 19.48 | 19.48 | [92.6](file:///E:\millscripts\viewfeed.pl%3fviewer=viewMaster.jar&side=spectrumWin&spectrumFiles=msdataSM\140307-00027-DP\9080\cpick_in\9080-Pos-aMSMS-30_47.4957.4962.0.pkl&mstagHits=LDHKFDLMYAK&cycle=1&fixedMods=carbamidomethylation&varMods=) | 13.22 | (R)LDHKFDLMYAK(R) |
|  |  | 19.22 | 15.27 | [91.2](file:///E:\millscripts\viewfeed.pl%3fviewer=viewMaster.jar&side=spectrumWin&spectrumFiles=msdataSM\140307-00027-DP\9080\cpick_in\9080-Pos-aMSMS-30_47.6411.6459.2.pkl&mstagHits=SIQFVDWCPTGFK&cycle=1&fixedMods=carbamidomethylation&varMods=) | 19.30 | (R)SIQFVDWCPTGFK(V) + C |
|  |  | 18.92 | 18.92 | [93.3](file:///E:\millscripts\viewfeed.pl%3fviewer=viewMaster.jar&side=spectrumWin&spectrumFiles=msdataSM\140307-00027-DP\9080\cpick_in\9080-Pos-aMSMS-30_47.6235.6350.3.pkl&mstagHits=IHFPLATYAPVISAEK&cycle=1&fixedMods=carbamidomethylation&varMods=) | 18.56 | (R)IHFPLATYAPVISAEK(A) |
|  |  | 18.77 | 9.73 | [92.7](file:///E:\millscripts\viewfeed.pl%3fviewer=viewMaster.jar&side=spectrumWin&spectrumFiles=msdataSM\140307-00027-DP\9080\cpick_in\9080-Pos-aMSMS-30_47.4946.4976.3.pkl&mstagHits=LDHKFDLMYAK&cycle=1&fixedMods=carbamidomethylation&varMods=) | 13.17 | (R)LDHKFDLMYAK(R) |
|  |  | 18.15 | 18.15 | [93](file:///E:\millscripts\viewfeed.pl%3fviewer=viewMaster.jar&side=spectrumWin&spectrumFiles=msdataSM\140307-00027-DP\9080\cpick_in\9080-Pos-aMSMS-30_47.7394.7398.0.pkl&mstagHits=LIGQIVSSITASLR&cycle=1&fixedMods=carbamidomethylation&varMods=).0 | 22.35 | (R)LIGQIVSSITASLR(F) |
|  |  | 18.09 | 12.40 | [90.9](file:///E:\millscripts\viewfeed.pl%3fviewer=viewMaster.jar&side=spectrumWin&spectrumFiles=msdataSM\140307-00027-DP\9080\cpick_in\9080-Pos-aMSMS-30_47.4877.4986.2.pkl&mstagHits=DVNAAIATIK&cycle=1&fixedMods=carbamidomethylation&varMods=) | 12.88 | (K)DVNAAIATIK(T) |
|  |  | 17.76 | 17.76 | [89.6](file:///E:\millscripts\viewfeed.pl%3fviewer=viewMaster.jar&side=spectrumWin&spectrumFiles=msdataSM\140307-00027-DP\9080\cpick_in\9080-Pos-aMSMS-30_47.5531.5594.2.pkl&mstagHits=QLFHPEQLITGK&cycle=1&fixedMods=carbamidomethylation&varMods=) | 15.56 | (R)QLFHPEQLITGK(E) |
|  |  | 17.55 | 8.87 | [92](file:///E:\millscripts\viewfeed.pl%3fviewer=viewMaster.jar&side=spectrumWin&spectrumFiles=msdataSM\140307-00027-DP\9080\cpick_in\9080-Pos-aMSMS-30_47.6161.6219.2.pkl&mstagHits=EIIDLVLDR&cycle=1&fixedMods=carbamidomethylation&varMods=).0 | 18.25 | (K)EIIDLVLDR(I) |
|  |  | 17.43 | 7.97 | [79.5](file:///E:\millscripts\viewfeed.pl%3fviewer=viewMaster.jar&side=spectrumWin&spectrumFiles=msdataSM\140307-00027-DP\9080\cpick_in\9080-Pos-aMSMS-30_47.4919.4952.3.pkl&mstagHits=NLDIERPTYTNLNR&cycle=1&fixedMods=carbamidomethylation&varMods=) | 13.05 | (R)NLDIERPTYTNLNR(L) |
|  |  | 17.42 | 17.42 | [83.1](file:///E:\millscripts\viewfeed.pl%3fviewer=viewMaster.jar&side=spectrumWin&spectrumFiles=msdataSM\140307-00027-DP\9080\cpick_in\9080-Pos-aMSMS-30_47.5235.5271.2.pkl&mstagHits=FDLMYAK&cycle=1&fixedMods=carbamidomethylation&varMods=) | 14.33 | (K)FDLMYAK(R) |
|  |  | 17.38 | 9.32 | [93.7](file:///E:\millscripts\viewfeed.pl%3fviewer=viewMaster.jar&side=spectrumWin&spectrumFiles=msdataSM\140307-00027-DP\9080\cpick_in\9080-Pos-aMSMS-30_47.6082.6181.0.pkl&mstagHits=EIIDLVLDR&cycle=1&fixedMods=carbamidomethylation&varMods=) | 17.91 | (K)EIIDLVLDR(I) |
|  |  | 17.14 | 17.14 | [65.9](file:///E:\millscripts\viewfeed.pl%3fviewer=viewMaster.jar&side=spectrumWin&spectrumFiles=msdataSM\140307-00027-DP\9080\cpick_in\9080-Pos-aMSMS-30_47.6371.6405.0.pkl&mstagHits=SFGGGTGSGFTSLLMER&cycle=1&fixedMods=carbamidomethylation&varMods=) | 19.13 | (R)SFGGGTGSGFTSLLMER(L) |
|  |  | 17.02 | 17.02 | [72.6](file:///E:\millscripts\viewfeed.pl%3fviewer=viewMaster.jar&side=spectrumWin&spectrumFiles=msdataSM\140307-00027-DP\9080\cpick_in\9080-Pos-aMSMS-30_47.6492.6496.0.pkl&mstagHits=AVCMLSNTTAIAEAWAR&cycle=1&fixedMods=carbamidomethylation&varMods=) | 19.66 | (R)AVCMLSNTTAIAEAWAR(L) + C |
|  |  | 16.79 | 16.79 | [84](file:///E:\millscripts\viewfeed.pl%3fviewer=viewMaster.jar&side=spectrumWin&spectrumFiles=msdataSM\140307-00027-DP\9080\cpick_in\9080-Pos-aMSMS-30_47.6370.6370.3.pkl&mstagHits=IHFPLATYAPVISAEK&cycle=1&fixedMods=carbamidomethylation&varMods=).0 | 19.12 | (R)IHFPLATYAPVISAEK(A) |
|  |  | 16.57 | 9.40 | [94.2](file:///E:\millscripts\viewfeed.pl%3fviewer=viewMaster.jar&side=spectrumWin&spectrumFiles=msdataSM\140307-00027-DP\9080\cpick_in\9080-Pos-aMSMS-30_47.5598.5665.2.pkl&mstagHits=VGINYQPPTVVPGGDLAK&cycle=1&fixedMods=carbamidomethylation&varMods=) | 15.85 | (K)VGINYQPPTVVPGGDLAK(V) |
|  |  | 16.33 | 8.95 | [83.7](file:///E:\millscripts\viewfeed.pl%3fviewer=viewMaster.jar&side=spectrumWin&spectrumFiles=msdataSM\140307-00027-DP\9080\cpick_in\9080-Pos-aMSMS-30_47.4939.4944.2.pkl&mstagHits=NLDIERPTYTNLNR&cycle=1&fixedMods=carbamidomethylation&varMods=) | 13.14 | (R)NLDIERPTYTNLNR(L) |
|  |  | 15.83 | 15.83 | [86.4](file:///E:\millscripts\viewfeed.pl%3fviewer=viewMaster.jar&side=spectrumWin&spectrumFiles=msdataSM\140307-00027-DP\9080\cpick_in\9080-Pos-aMSMS-30_47.7575.7608.0.pkl&mstagHits=LISQIVSSITASLR&cycle=1&fixedMods=carbamidomethylation&varMods=) | 22.81 | (R)LISQIVSSITASLR(F) |
|  |  | 15.72 | 10.88 | [77.5](file:///E:\millscripts\viewfeed.pl%3fviewer=viewMaster.jar&side=spectrumWin&spectrumFiles=msdataSM\140307-00027-DP\9080\cpick_in\9080-Pos-aMSMS-30_47.6187.6192.0.pkl&mstagHits=AVCMLSNTTAVAEAWAR&cycle=1&fixedMods=carbamidomethylation&varMods=) | 18.36 | (R)AVCMLSNTTAVAEAWAR(L) + C |
|  |  | 15.53 | 15.53 | [63.8](file:///E:\millscripts\viewfeed.pl%3fviewer=viewMaster.jar&side=spectrumWin&spectrumFiles=msdataSM\140307-00027-DP\9080\cpick_in\9080-Pos-aMSMS-30_47.4862.4963.0.pkl&mstagHits=NLDIERPTYTNLNR&cycle=1&fixedMods=carbamidomethylation&varMods=) | 12.81 | (R)NLDIERPTYTNLNR(L) |
|  |  | 15.00 | 4.40 | [91.4](file:///E:\millscripts\viewfeed.pl%3fviewer=viewMaster.jar&side=spectrumWin&spectrumFiles=msdataSM\140307-00027-DP\9080\cpick_in\9080-Pos-aMSMS-30_47.3747.3770.2.pkl&mstagHits=LSVDYGK&cycle=1&fixedMods=carbamidomethylation&varMods=) | 9.27 | (R)LSVDYGK(K) |
|  |  | 14.89 | 9.90 | [88.1](file:///E:\millscripts\viewfeed.pl%3fviewer=viewMaster.jar&side=spectrumWin&spectrumFiles=msdataSM\140307-00027-DP\9080\cpick_in\9080-Pos-aMSMS-30_47.5603.5649.2.pkl&mstagHits=VGINYQPPTVVPGGDLAK&cycle=1&fixedMods=carbamidomethylation&varMods=) | 15.87 | (K)VGINYQPPTVVPGGDLAK(V) |
|  |  | 14.32 | 14.32 | [77.6](file:///E:\millscripts\viewfeed.pl%3fviewer=viewMaster.jar&side=spectrumWin&spectrumFiles=msdataSM\140307-00027-DP\9080\cpick_in\9080-Pos-aMSMS-30_47.2515.2515.0.pkl&mstagHits=EDAANNYAR&cycle=1&fixedMods=carbamidomethylation&varMods=) | 6.10 | (K)EDAANNYAR(G) |
|  |  | 14.16 | 9.41 | [81.7](file:///E:\millscripts\viewfeed.pl%3fviewer=viewMaster.jar&side=spectrumWin&spectrumFiles=msdataSM\140307-00027-DP\9080\cpick_in\9080-Pos-aMSMS-30_47.6381.6396.0.pkl&mstagHits=AVFVDLEPTVVDEVR&cycle=1&fixedMods=carbamidomethylation&varMods=) | 19.17 | (R)AVFVDLEPTVVDEVR(T) |
|  |  | 13.75 | 13.75 | [76.8](file:///E:\millscripts\viewfeed.pl%3fviewer=viewMaster.jar&side=spectrumWin&spectrumFiles=msdataSM\140307-00027-DP\9080\cpick_in\9080-Pos-aMSMS-30_47.6193.6198.0.pkl&mstagHits=IHFPLATYAPVISAEK&cycle=1&fixedMods=carbamidomethylation&varMods=) | 18.39 | (R)IHFPLATYAPVISAEK(A) |
|  |  | 13.68 | 13.68 | [81.6](file:///E:\millscripts\viewfeed.pl%3fviewer=viewMaster.jar&side=spectrumWin&spectrumFiles=msdataSM\140307-00027-DP\9080\cpick_in\9080-Pos-aMSMS-30_47.5314.5343.0.pkl&mstagHits=DYEEVGVDSVEGEGEEEGEEY&cycle=1&fixedMods=carbamidomethylation&varMods=) | 14.66 | (K)DYEEVGVDSVEGEGEEEGEEY(-) |
|  |  | 12.87 | 12.87 | [71.9](file:///E:\millscripts\viewfeed.pl%3fviewer=viewMaster.jar&side=spectrumWin&spectrumFiles=msdataSM\140307-00027-DP\9080\cpick_in\9080-Pos-aMSMS-30_47.6541.6631.0.pkl&mstagHits=AVFVDLEPTVIDEVR&cycle=1&fixedMods=carbamidomethylation&varMods=) | 19.87 | (R)AVFVDLEPTVIDEVR(T) |
|  |  | 12.84 | 12.84 | [74.2](file:///E:\millscripts\viewfeed.pl%3fviewer=viewMaster.jar&side=spectrumWin&spectrumFiles=msdataSM\140307-00027-DP\9080\cpick_in\9080-Pos-aMSMS-30_47.5112.5117.0.pkl&mstagHits=DVNAAIAAIK&cycle=1&fixedMods=carbamidomethylation&varMods=) | 13.84 | (K)DVNAAIAAIK(T) |
|  |  | 12.73 | 12.73 | [72.6](file:///E:\millscripts\viewfeed.pl%3fviewer=viewMaster.jar&side=spectrumWin&spectrumFiles=msdataSM\140307-00027-DP\9080\cpick_in\9080-Pos-aMSMS-30_47.5530.5576.3.pkl&mstagHits=QLFHPEQLITGK&cycle=1&fixedMods=carbamidomethylation&varMods=) | 15.55 | (R)QLFHPEQLITGK(E) |
|  |  | 12.36 | 12.36 | [72.6](file:///E:\millscripts\viewfeed.pl%3fviewer=viewMaster.jar&side=spectrumWin&spectrumFiles=msdataSM\140307-00027-DP\9080\cpick_in\9080-Pos-aMSMS-30_47.4367.4402.0.pkl&mstagHits=EDLAALEK&cycle=1&fixedMods=carbamidomethylation&varMods=) | 11.07 | (R)EDLAALEK(D) |
|  |  | 12.00 | 12.00 | [68](file:///E:\millscripts\viewfeed.pl%3fviewer=viewMaster.jar&side=spectrumWin&spectrumFiles=msdataSM\140307-00027-DP\9080\cpick_in\9080-Pos-aMSMS-30_47.3761.3817.2.pkl&mstagHits=EDMAALEK&cycle=1&fixedMods=carbamidomethylation&varMods=).0 | 9.34 | (R)EDMAALEK(D) |
|  |  | 11.90 | 11.90 | [69.8](file:///E:\millscripts\viewfeed.pl%3fviewer=viewMaster.jar&side=spectrumWin&spectrumFiles=msdataSM\140307-00027-DP\9080\cpick_in\9080-Pos-aMSMS-30_47.4622.4622.0.pkl&mstagHits=RNLDIERPTYTNLNR&cycle=1&fixedMods=carbamidomethylation&varMods=) | 12.01 | (R)RNLDIERPTYTNLNR(L) |
|  |  | 10.66 | 10.66 | [72](file:///E:\millscripts\viewfeed.pl%3fviewer=viewMaster.jar&side=spectrumWin&spectrumFiles=msdataSM\140307-00027-DP\9080\cpick_in\9080-Pos-aMSMS-30_47.5655.5688.0.pkl&mstagHits=VGINYQPPTVVPGGDLAK&cycle=1&fixedMods=carbamidomethylation&varMods=).0 | 16.09 | (K)VGINYQPPTVVPGGDLAK(V) |
|  |  | 10.36 | 10.36 | [77.5](file:///E:\millscripts\viewfeed.pl%3fviewer=viewMaster.jar&side=spectrumWin&spectrumFiles=msdataSM\140307-00027-DP\9080\cpick_in\9080-Pos-aMSMS-30_47.6006.6006.0.pkl&mstagHits=AYHEQLSVAEITNACFEPANQMVK&cycle=1&fixedMods=carbamidomethylation&varMods=) | 17.58 | (K)AYHEQLSVAEITNACFEPANQMVK(C) + C |
|  |  | 10.18 | 10.18 | [63.2](file:///E:\millscripts\viewfeed.pl%3fviewer=viewMaster.jar&side=spectrumWin&spectrumFiles=msdataSM\140307-00027-DP\9080\cpick_in\9080-Pos-aMSMS-30_47.6034.6042.0.pkl&mstagHits=RSIQFVDWCPTGFK&cycle=1&fixedMods=carbamidomethylation&varMods=) | 17.70 | (K)RSIQFVDWCPTGFK(V) + C |
|  |  | 9.86 | 9.86 | [69.7](file:///E:\millscripts\viewfeed.pl%3fviewer=viewMaster.jar&side=spectrumWin&spectrumFiles=msdataSM\140307-00027-DP\9080\cpick_in\9080-Pos-aMSMS-30_47.6011.6011.0.pkl&mstagHits=AYHEQLSVAEITNACFEPANQMVK&cycle=1&fixedMods=carbamidomethylation&varMods=) | 17.60 | (K)AYHEQLSVAEITNACFEPANQMVK(C) + C |
|  |  | 9.82 | 0.67 | [61.2](file:///E:\millscripts\viewfeed.pl%3fviewer=viewMaster.jar&side=spectrumWin&spectrumFiles=msdataSM\140307-00027-DP\9080\cpick_in\9080-Pos-aMSMS-30_47.5336.5339.0.pkl&mstagHits=EIIDPVLDR&cycle=1&fixedMods=carbamidomethylation&varMods=) | 14.76 | (K)EIIDPVLDR(I) |
|  |  | 9.79 | 9.79 | [70.7](file:///E:\millscripts\viewfeed.pl%3fviewer=viewMaster.jar&side=spectrumWin&spectrumFiles=msdataSM\140307-00027-DP\9080\cpick_in\9080-Pos-aMSMS-30_47.6688.6688.0.pkl&mstagHits=LISQIVSSITASLR&cycle=1&fixedMods=carbamidomethylation&varMods=) | 20.42 | (R)LISQIVSSITASLR(F) |
|  |  | 9.33 | 9.33 | [65](file:///E:\millscripts\viewfeed.pl%3fviewer=viewMaster.jar&side=spectrumWin&spectrumFiles=msdataSM\140307-00027-DP\9080\cpick_in\9080-Pos-aMSMS-30_47.4103.4103.0.pkl&mstagHits=EDMAALEK&cycle=1&fixedMods=carbamidomethylation&varMods=).0 | 10.29 | (R)EDMAALEK(D) |
|  |  | 9.00 | 9.00 | [67.7](file:///E:\millscripts\viewfeed.pl%3fviewer=viewMaster.jar&side=spectrumWin&spectrumFiles=msdataSM\140307-00027-DP\9080\cpick_in\9080-Pos-aMSMS-30_47.6695.6695.0.pkl&mstagHits=LISQIVSSITASLR&cycle=1&fixedMods=carbamidomethylation&varMods=) | 20.45 | (R)LISQIVSSITASLR(F) |
|  |  | 8.37 | 3.23 | [63.1](file:///E:\millscripts\viewfeed.pl%3fviewer=viewMaster.jar&side=spectrumWin&spectrumFiles=msdataSM\140307-00027-DP\9080\cpick_in\9080-Pos-aMSMS-30_47.5700.5700.0.pkl&mstagHits=EIIDLVLDR&cycle=1&fixedMods=carbamidomethylation&varMods=) | 16.29 | (K)EIIDLVLDR(I) |
|  |  | 7.77 | 7.77 | [66.9](file:///E:\millscripts\viewfeed.pl%3fviewer=viewMaster.jar&side=spectrumWin&spectrumFiles=msdataSM\140307-00027-DP\9080\cpick_in\9080-Pos-aMSMS-30_47.4595.4595.0.pkl&mstagHits=DYEEVGADSADGEDEGEEY&cycle=1&fixedMods=carbamidomethylation&varMods=) | 11.89 | (K)DYEEVGADSADGEDEGEEY  (-) |
|  |  |  |  |  |  |  |
| 17 | T-complex protein 1 | 21.11 | 21.11 | [92.9](file:///E:\millscripts\viewfeed.pl%3fviewer=viewMaster.jar&side=spectrumWin&spectrumFiles=msdataSM\140307-00027-DP\13670\cpick_in\13670-Pos-aMSMS-30_47.7008.7078.0.pkl&mstagHits=FATEAAITILR&cycle=1&fixedMods=carbamidomethylation&varMods=) | 17.21 | (K)FATEAAITILR(I) |
|  | subunit alpha | 16.69 | 16.69 | [80](file:///E:\millscripts\viewfeed.pl%3fviewer=viewMaster.jar&side=spectrumWin&spectrumFiles=msdataSM\140307-00027-DP\13670\cpick_in\13670-Pos-aMSMS-30_47.6637.6659.0.pkl&mstagHits=IACLDFSLQK&cycle=1&fixedMods=carbamidomethylation&varMods=).0 | 16.12 | (K)IACLDFSLQK(T) + C |
|  |  | 16.04 | 16.04 | [79.2](file:///E:\millscripts\viewfeed.pl%3fviewer=viewMaster.jar&side=spectrumWin&spectrumFiles=msdataSM\140307-00027-DP\13670\cpick_in\13670-Pos-aMSMS-30_47.6135.6147.0.pkl&mstagHits=LGVQVVITDPEK&cycle=1&fixedMods=carbamidomethylation&varMods=) | 14.89 | (K)LGVQVVITDPEK(L) |
|  |  | 12.17 | 12.17 | [69.7](file:///E:\millscripts\viewfeed.pl%3fviewer=viewMaster.jar&side=spectrumWin&spectrumFiles=msdataSM\140307-00027-DP\13670\cpick_in\13670-Pos-aMSMS-30_47.6792.6792.0.pkl&mstagHits=YINENLIVNTDELGR&cycle=1&fixedMods=carbamidomethylation&varMods=) | 16.56 | (R)YINENLIVNTDELGR(D) |
|  |  | 11.23 | 11.23 | [62.9](file:///E:\millscripts\viewfeed.pl%3fviewer=viewMaster.jar&side=spectrumWin&spectrumFiles=msdataSM\140307-00027-DP\13670\cpick_in\13670-Pos-aMSMS-30_47.6640.6640.0.pkl&mstagHits=IACLDFSLQK&cycle=1&fixedMods=carbamidomethylation&varMods=) | 16.14 | (K)IACLDFSLQK(T) + C |
|  |  |  |  |  |  |  |
| 18 | Elongation factor Tu | 22.19 | 22.19 | [99.2](file:///E:\millscripts\viewfeed.pl%3fviewer=viewMaster.jar&side=spectrumWin&spectrumFiles=msdataSM\140307-00027-DP\18459\cpick_in\18459-Pos-aMSMS-30_47.6877.6926.0.pkl&mstagHits=LLDAVDTYIPVPAR&cycle=1&fixedMods=carbamidomethylation&varMods=) | 18.11 | (K)LLDAVDTYIPVPAR(D) |
|  |  | 21.99 | 11.08 | [92](file:///E:\millscripts\viewfeed.pl%3fviewer=viewMaster.jar&side=spectrumWin&spectrumFiles=msdataSM\140307-00027-DP\18459\cpick_in\18459-Pos-aMSMS-30_47.5498.5513.0.pkl&mstagHits=TVVTGIEMFHK&cycle=1&fixedMods=carbamidomethylation&varMods=).0 | 14.41 | (R)TVVTGIEMFHK(S) |
|  |  | 21.60 | 21.60 | [92.1](file:///E:\millscripts\viewfeed.pl%3fviewer=viewMaster.jar&side=spectrumWin&spectrumFiles=msdataSM\140307-00027-DP\18459\cpick_in\18459-Pos-aMSMS-30_47.7842.7927.0.pkl&mstagHits=GEETPVIVGSALCALEGR&cycle=1&fixedMods=carbamidomethylation&varMods=) | 20.53 | (K)GEETPVIVGSALCALEGR(D) + C |
|  |  | 21.23 | 21.23 | [96.4](file:///E:\millscripts\viewfeed.pl%3fviewer=viewMaster.jar&side=spectrumWin&spectrumFiles=msdataSM\140307-00027-DP\18459\cpick_in\18459-Pos-aMSMS-30_47.6764.6856.0.pkl&mstagHits=TIGTGLVTNTLAMTEEEK&cycle=1&fixedMods=carbamidomethylation&varMods=) | 17.80 | (R)TIGTGLVTNTLAMTEEEK(N) |
|  |  | 20.21 | 11.52 | [80](file:///E:\millscripts\viewfeed.pl%3fviewer=viewMaster.jar&side=spectrumWin&spectrumFiles=msdataSM\140307-00027-DP\18459\cpick_in\18459-Pos-aMSMS-30_47.4920.5087.0.pkl&mstagHits=GITINAAHVEYSTAAR&cycle=1&fixedMods=carbamidomethylation&varMods=).0 | 12.59 | (R)GITINAAHVEYSTAAR(H) |
|  |  | 19.53 | 14.56 | [89.4](file:///E:\millscripts\viewfeed.pl%3fviewer=viewMaster.jar&side=spectrumWin&spectrumFiles=msdataSM\140307-00027-DP\18459\cpick_in\18459-Pos-aMSMS-30_47.5081.5142.2.pkl&mstagHits=AEAGDNLGALVR&cycle=1&fixedMods=carbamidomethylation&varMods=) | 13.07 | (R)AEAGDNLGALVR(G) |
|  |  | 19.48 | 8.91 | [91.5](file:///E:\millscripts\viewfeed.pl%3fviewer=viewMaster.jar&side=spectrumWin&spectrumFiles=msdataSM\140307-00027-DP\18459\cpick_in\18459-Pos-aMSMS-30_47.4924.4960.0.pkl&mstagHits=GITINAAHVEYSTAAR&cycle=1&fixedMods=carbamidomethylation&varMods=) | 12.62 | (R)GITINAAHVEYSTAAR(H) |
|  |  | 19.08 | 7.49 | [90.9](file:///E:\millscripts\viewfeed.pl%3fviewer=viewMaster.jar&side=spectrumWin&spectrumFiles=msdataSM\140307-00027-DP\18459\cpick_in\18459-Pos-aMSMS-30_47.3692.3695.0.pkl&mstagHits=YEEIDNAPEER&cycle=1&fixedMods=carbamidomethylation&varMods=) | 9.07 | (K)YEEIDNAPEER(A) |
|  |  | 18.44 | 9.98 | [81.6](file:///E:\millscripts\viewfeed.pl%3fviewer=viewMaster.jar&side=spectrumWin&spectrumFiles=msdataSM\140307-00027-DP\18459\cpick_in\18459-Pos-aMSMS-30_47.5497.5520.0.pkl&mstagHits=TVVTGIEMFHK&cycle=1&fixedMods=carbamidomethylation&varMods=) | 14.41 | (R)TVVTGIEMFHK(S) |
|  |  | 17.97 | 9.33 | [87.3](file:///E:\millscripts\viewfeed.pl%3fviewer=viewMaster.jar&side=spectrumWin&spectrumFiles=msdataSM\140307-00027-DP\18459\cpick_in\18459-Pos-aMSMS-30_47.5986.5989.0.pkl&mstagHits=ELLTEFGYK&cycle=1&fixedMods=carbamidomethylation&varMods=) | 15.67 | (R)ELLTEFGYK(G) |
|  |  | 17.13 | 3.31 | [89.8](file:///E:\millscripts\viewfeed.pl%3fviewer=viewMaster.jar&side=spectrumWin&spectrumFiles=msdataSM\140307-00027-DP\18459\cpick_in\18459-Pos-aMSMS-30_47.4397.4435.0.pkl&mstagHits=TTLTAAITK&cycle=1&fixedMods=carbamidomethylation&varMods=) | 11.02 | (K)TTLTAAITK(I) |
|  |  | 17.08 | 12.62 | [86.5](file:///E:\millscripts\viewfeed.pl%3fviewer=viewMaster.jar&side=spectrumWin&spectrumFiles=msdataSM\140307-00027-DP\18459\cpick_in\18459-Pos-aMSMS-30_47.5082.5103.2.pkl&mstagHits=ELAMPGEDLK&cycle=1&fixedMods=carbamidomethylation&varMods=) | 13.07 | (K)ELAMPGEDLK(F) |
|  |  | 16.95 | 6.21 | [92.2](file:///E:\millscripts\viewfeed.pl%3fviewer=viewMaster.jar&side=spectrumWin&spectrumFiles=msdataSM\140307-00027-DP\18459\cpick_in\18459-Pos-aMSMS-30_47.4621.4624.0.pkl&mstagHits=IILPPEK&cycle=1&fixedMods=carbamidomethylation&varMods=) | 11.70 | (R)IILPPEK(E) |
|  |  | 16.80 | 16.80 | [82.3](file:///E:\millscripts\viewfeed.pl%3fviewer=viewMaster.jar&side=spectrumWin&spectrumFiles=msdataSM\140307-00027-DP\18459\cpick_in\18459-Pos-aMSMS-30_47.3437.3440.0.pkl&mstagHits=KYEEIDNAPEER&cycle=1&fixedMods=carbamidomethylation&varMods=) | 8.51 | (K)KYEEIDNAPEER(A) |
|  |  | 16.09 | 8.69 | [81](file:///E:\millscripts\viewfeed.pl%3fviewer=viewMaster.jar&side=spectrumWin&spectrumFiles=msdataSM\140307-00027-DP\18459\cpick_in\18459-Pos-aMSMS-30_47.5255.5255.2.pkl&mstagHits=VEAQVYILSK&cycle=1&fixedMods=carbamidomethylation&varMods=).0 | 13.68 | (K)VEAQVYILSK(E) |
|  |  | 15.59 | 15.59 | [81.4](file:///E:\millscripts\viewfeed.pl%3fviewer=viewMaster.jar&side=spectrumWin&spectrumFiles=msdataSM\140307-00027-DP\18459\cpick_in\18459-Pos-aMSMS-30_47.5145.5153.0.pkl&mstagHits=QIGVEHVVVYVNK&cycle=1&fixedMods=carbamidomethylation&varMods=) | 13.30 | (R)QIGVEHVVVYVNK(A) |
|  |  | 14.55 | 14.55 | [71](file:///E:\millscripts\viewfeed.pl%3fviewer=viewMaster.jar&side=spectrumWin&spectrumFiles=msdataSM\140307-00027-DP\18459\cpick_in\18459-Pos-aMSMS-30_47.5136.5163.0.pkl&mstagHits=QIGVEHVVVYVNK&cycle=1&fixedMods=carbamidomethylation&varMods=).0 | 13.26 | (R)QIGVEHVVVYVNK(A) |
|  |  | 13.85 | 13.85 | [83.8](file:///E:\millscripts\viewfeed.pl%3fviewer=viewMaster.jar&side=spectrumWin&spectrumFiles=msdataSM\140307-00027-DP\18459\cpick_in\18459-Pos-aMSMS-30_47.3433.3436.0.pkl&mstagHits=KYEEIDNAPEER&cycle=1&fixedMods=carbamidomethylation&varMods=) | 8.49 | (K)KYEEIDNAPEER(A) |
|  |  | 13.77 | 13.77 | [82.9](file:///E:\millscripts\viewfeed.pl%3fviewer=viewMaster.jar&side=spectrumWin&spectrumFiles=msdataSM\140307-00027-DP\18459\cpick_in\18459-Pos-aMSMS-30_47.8264.8264.0.pkl&mstagHits=DLEKPFLLPVEAVYSVPGR&cycle=1&fixedMods=carbamidomethylation&varMods=) | 21.45 | (R)DLEKPFLLPVEAVYSVPGR  (G) |
|  |  | 13.64 | 13.64 | [61.8](file:///E:\millscripts\viewfeed.pl%3fviewer=viewMaster.jar&side=spectrumWin&spectrumFiles=msdataSM\140307-00027-DP\18459\cpick_in\18459-Pos-aMSMS-30_47.3754.3754.0.pkl&mstagHits=GDECELLGHSK&cycle=1&fixedMods=carbamidomethylation&varMods=) | 9.31 | (K)GDECELLGHSK(N) + C |
|  |  | 13.57 | 6.53 | [85.7](file:///E:\millscripts\viewfeed.pl%3fviewer=viewMaster.jar&side=spectrumWin&spectrumFiles=msdataSM\140307-00027-DP\18459\cpick_in\18459-Pos-aMSMS-30_47.4091.4101.2.pkl&mstagHits=GTVVTGTLER&cycle=1&fixedMods=carbamidomethylation&varMods=) | 10.15 | (R)GTVVTGTLER(G) |
|  |  | 13.53 | 13.53 | [73.1](file:///E:\millscripts\viewfeed.pl%3fviewer=viewMaster.jar&side=spectrumWin&spectrumFiles=msdataSM\140307-00027-DP\18459\cpick_in\18459-Pos-aMSMS-30_47.3345.3348.0.pkl&mstagHits=KGDECELLGHSK&cycle=1&fixedMods=carbamidomethylation&varMods=) | 8.25 | (K)KGDECELLGHSK(N) + C |
|  |  | 13.43 | 4.61 | [82.2](file:///E:\millscripts\viewfeed.pl%3fviewer=viewMaster.jar&side=spectrumWin&spectrumFiles=msdataSM\140307-00027-DP\18459\cpick_in\18459-Pos-aMSMS-30_47.6074.6104.2.pkl&mstagHits=FNLILR&cycle=1&fixedMods=carbamidomethylation&varMods=) | 15.93 | (K)FNLILR(Q) |
|  |  | 13.32 | 13.32 | [72.9](file:///E:\millscripts\viewfeed.pl%3fviewer=viewMaster.jar&side=spectrumWin&spectrumFiles=msdataSM\140307-00027-DP\18459\cpick_in\18459-Pos-aMSMS-30_47.7845.7845.0.pkl&mstagHits=GEETPVIVGSALCALEGR&cycle=1&fixedMods=carbamidomethylation&varMods=) | 20.54 | (K)GEETPVIVGSALCALEGR(D) + C |
|  |  | 12.95 | 5.28 | [77.1](file:///E:\millscripts\viewfeed.pl%3fviewer=viewMaster.jar&side=spectrumWin&spectrumFiles=msdataSM\140307-00027-DP\18459\cpick_in\18459-Pos-aMSMS-30_47.4202.4205.0.pkl&mstagHits=DPELGLK&cycle=1&fixedMods=carbamidomethylation&varMods=) | 10.49 | (R)DPELGLK(S) |
|  |  | 12.50 | 2.43 | [78.9](file:///E:\millscripts\viewfeed.pl%3fviewer=viewMaster.jar&side=spectrumWin&spectrumFiles=msdataSM\140307-00027-DP\18459\cpick_in\18459-Pos-aMSMS-30_47.4206.4209.0.pkl&mstagHits=EHLLLAR&cycle=1&fixedMods=carbamidomethylation&varMods=) | 10.50 | (R)EHLLLAR(Q) |
|  |  | 9.15 | 9.15 | [78.9](file:///E:\millscripts\viewfeed.pl%3fviewer=viewMaster.jar&side=spectrumWin&spectrumFiles=msdataSM\140307-00027-DP\18459\cpick_in\18459-Pos-aMSMS-30_47.6905.6908.0.pkl&mstagHits=LLDAVDTYIPVPAR&cycle=1&fixedMods=carbamidomethylation&varMods=) | 18.20 | (K)LLDAVDTYIPVPAR(D) |
|  |  | 7.88 | 7.88 | [74.4](file:///E:\millscripts\viewfeed.pl%3fviewer=viewMaster.jar&side=spectrumWin&spectrumFiles=msdataSM\140307-00027-DP\18459\cpick_in\18459-Pos-aMSMS-30_47.4458.4461.0.pkl&mstagHits=IILPPEK&cycle=1&fixedMods=carbamidomethylation&varMods=) | 11.18 | (R)IILPPEK(E) |
|  |  | 7.56 | 7.56 | [71.9](file:///E:\millscripts\viewfeed.pl%3fviewer=viewMaster.jar&side=spectrumWin&spectrumFiles=msdataSM\140307-00027-DP\18459\cpick_in\18459-Pos-aMSMS-30_47.4788.4792.0.pkl&mstagHits=IILPPEK&cycle=1&fixedMods=carbamidomethylation&varMods=) | 12.23 | (R)IILPPEK(E) |
|  |  | 7.32 | 7.32 | [62](file:///E:\millscripts\viewfeed.pl%3fviewer=viewMaster.jar&side=spectrumWin&spectrumFiles=msdataSM\140307-00027-DP\18459\cpick_in\18459-Pos-aMSMS-30_47.5252.5252.2.pkl&mstagHits=VEAQVYILSK&cycle=1&fixedMods=carbamidomethylation&varMods=).0 | 13.67 | (K)VEAQVYILSK(E) |
|  |  |  |  |  |  |  |
| 19 | Protein disulfide- | 18.87 | 18.87 | [90.8](file:///E:\millscripts\viewfeed.pl%3fviewer=viewMaster.jar&side=spectrumWin&spectrumFiles=msdataSM\140307-00027-DP\18942\cpick_in\18942-Pos-aMSMS-30_47.7010.7022.0.pkl&mstagHits=LYSSSDDVIELTPSNFNR&cycle=1&fixedMods=carbamidomethylation&varMods=) | 17.05 | (-)LYSSSDDVIELTPSNFNR(E) |
|  | isomerase A | 16.49 | 16.49 | [77.4](file:///E:\millscripts\viewfeed.pl%3fviewer=viewMaster.jar&side=spectrumWin&spectrumFiles=msdataSM\140307-00027-DP\18942\cpick_in\18942-Pos-aMSMS-30_47.6511.6511.0.pkl&mstagHits=GSTAPVGGGAFPTIVER&cycle=1&fixedMods=carbamidomethylation&varMods=) | 15.84 | (R)GSTAPVGGGAFPTIVER(E) |
|  |  | 11.82 | 11.82 | [70.4](file:///E:\millscripts\viewfeed.pl%3fviewer=viewMaster.jar&side=spectrumWin&spectrumFiles=msdataSM\140307-00027-DP\18942\cpick_in\18942-Pos-aMSMS-30_47.8730.8730.0.pkl&mstagHits=TGEAIVDAALSALR&cycle=1&fixedMods=carbamidomethylation&varMods=) | 21.09 | (R)TGEAIVDAALSALR(Q) |
|  |  | 10.87 | 10.87 | [66](file:///E:\millscripts\viewfeed.pl%3fviewer=viewMaster.jar&side=spectrumWin&spectrumFiles=msdataSM\140307-00027-DP\18942\cpick_in\18942-Pos-aMSMS-30_47.6501.6507.0.pkl&mstagHits=GSTAPVGGGAFPTIVER&cycle=1&fixedMods=carbamidomethylation&varMods=).0 | 15.79 | (R)GSTAPVGGGAFPTIVER(E) |
|  |  |  |  |  |  |  |
| 20 | Glutamate | 25.40 | 25.40 | [98.5](file:///E:\millscripts\viewfeed.pl%3fviewer=viewMaster.jar&side=spectrumWin&spectrumFiles=msdataSM\140307-00027-DP\8729\cpick_in\8729-Pos-aMSMS-30_47.6942.6964.0.pkl&mstagHits=TFVVQGFGNVGLHSMR&cycle=1&fixedMods=carbamidomethylation&varMods=) | 17.23 | (K)TFVVQGFGNVGLHSMR(Y) |
|  | dehydrogenase 1 | 21.88 | 21.88 | [88.1](file:///E:\millscripts\viewfeed.pl%3fviewer=viewMaster.jar&side=spectrumWin&spectrumFiles=msdataSM\140307-00027-DP\8729\cpick_in\8729-Pos-aMSMS-30_47.6456.6527.0.pkl&mstagHits=IIAEGANGPTTPEADKIFLER&cycle=1&fixedMods=carbamidomethylation&varMods=) | 15.82 | (K)IIAEGANGPTTPEADKIFLER  (N) |
|  |  | 20.66 | 20.66 | [85.3](file:///E:\millscripts\viewfeed.pl%3fviewer=viewMaster.jar&side=spectrumWin&spectrumFiles=msdataSM\140307-00027-DP\8729\cpick_in\8729-Pos-aMSMS-30_47.7159.7172.0.pkl&mstagHits=GFIGPGIDVPAPDMSTGER&cycle=1&fixedMods=carbamidomethylation&varMods=) | 17.91 | (K)GFIGPGIDVPAPDMSTGER(E) |
|  |  | 19.13 | 19.13 | [91.2](file:///E:\millscripts\viewfeed.pl%3fviewer=viewMaster.jar&side=spectrumWin&spectrumFiles=msdataSM\140307-00027-DP\8729\cpick_in\8729-Pos-aMSMS-30_47.6103.6107.0.pkl&mstagHits=DIVHSGLAYTMER&cycle=1&fixedMods=carbamidomethylation&varMods=) | 14.88 | (K)DIVHSGLAYTMER(S) |
|  |  | 18.04 | 13.23 | [82.4](file:///E:\millscripts\viewfeed.pl%3fviewer=viewMaster.jar&side=spectrumWin&spectrumFiles=msdataSM\140307-00027-DP\8729\cpick_in\8729-Pos-aMSMS-30_47.6945.6949.0.pkl&mstagHits=TFVVQGFGNVGLHSMR&cycle=1&fixedMods=carbamidomethylation&varMods=) | 17.27 | (K)TFVVQGFGNVGLHSMR(Y) |
|  |  | 16.14 | 16.14 | [85.1](file:///E:\millscripts\viewfeed.pl%3fviewer=viewMaster.jar&side=spectrumWin&spectrumFiles=msdataSM\140307-00027-DP\8729\cpick_in\8729-Pos-aMSMS-30_47.6385.6389.0.pkl&mstagHits=DDGSWEVIEGYR&cycle=1&fixedMods=carbamidomethylation&varMods=) | 15.63 | (R)DDGSWEVIEGYR(A) |
|  |  | 16.04 | 16.04 | [91.1](file:///E:\millscripts\viewfeed.pl%3fviewer=viewMaster.jar&side=spectrumWin&spectrumFiles=msdataSM\140307-00027-DP\8729\cpick_in\8729-Pos-aMSMS-30_47.6386.6397.0.pkl&mstagHits=HGGTIPIVPTAEFQDR&cycle=1&fixedMods=carbamidomethylation&varMods=) | 15.63 | (K)HGGTIPIVPTAEFQDR(I) |
|  |  | 15.80 | 15.80 | [72](file:///E:\millscripts\viewfeed.pl%3fviewer=viewMaster.jar&side=spectrumWin&spectrumFiles=msdataSM\140307-00027-DP\8729\cpick_in\8729-Pos-aMSMS-30_47.6793.6793.0.pkl&mstagHits=CIAVGESDGSIWNPDGIDPK&cycle=1&fixedMods=carbamidomethylation&varMods=).0 | 16.89 | (K)CIAVGESDGSIWNPDGIDPK  (E) + C |
|  |  | 15.73 | 15.73 | [86.9](file:///E:\millscripts\viewfeed.pl%3fviewer=viewMaster.jar&side=spectrumWin&spectrumFiles=msdataSM\140307-00027-DP\8729\cpick_in\8729-Pos-aMSMS-30_47.7305.7375.0.pkl&mstagHits=AKPYEGSILEADCDILIPAASEK&cycle=1&fixedMods=carbamidomethylation&varMods=) | 18.31 | (K)AKPYEGSILEADCDILIPAASEK(Q) + C |
|  |  | 14.14 | 7.12 | [86.6](file:///E:\millscripts\viewfeed.pl%3fviewer=viewMaster.jar&side=spectrumWin&spectrumFiles=msdataSM\140307-00027-DP\8729\cpick_in\8729-Pos-aMSMS-30_47.6394.6406.0.pkl&mstagHits=HGGTIPIVPTAEFQDR&cycle=1&fixedMods=carbamidomethylation&varMods=) | 15.67 | (K)HGGTIPIVPTAEFQDR(I) |
|  |  | 13.73 | 13.73 | [84.3](file:///E:\millscripts\viewfeed.pl%3fviewer=viewMaster.jar&side=spectrumWin&spectrumFiles=msdataSM\140307-00027-DP\8729\cpick_in\8729-Pos-aMSMS-30_47.6721.6745.0.pkl&mstagHits=KGFIGPGIDVPAPDMSTGER&cycle=1&fixedMods=carbamidomethylation&varMods=) | 16.62 | (K)KGFIGPGIDVPAPDMSTGER  (E) |
|  |  | 12.20 | 1.78 | [70.1](file:///E:\millscripts\viewfeed.pl%3fviewer=viewMaster.jar&side=spectrumWin&spectrumFiles=msdataSM\140307-00027-DP\8729\cpick_in\8729-Pos-aMSMS-30_47.6381.6384.0.pkl&mstagHits=YNLGLDLR&cycle=1&fixedMods=carbamidomethylation&varMods=) | 15.60 | (K)YNLGLDLR(T) |
|  |  | 12.15 | 12.15 | [70](file:///E:\millscripts\viewfeed.pl%3fviewer=viewMaster.jar&side=spectrumWin&spectrumFiles=msdataSM\140307-00027-DP\8729\cpick_in\8729-Pos-aMSMS-30_47.7278.7301.0.pkl&mstagHits=DSNYHLLMSVQESLER&cycle=1&fixedMods=carbamidomethylation&varMods=).0 | 18.22 | (R)DSNYHLLMSVQESLER(K) |
|  |  | 12.11 | 12.11 | [73.7](file:///E:\millscripts\viewfeed.pl%3fviewer=viewMaster.jar&side=spectrumWin&spectrumFiles=msdataSM\140307-00027-DP\8729\cpick_in\8729-Pos-aMSMS-30_47.7277.7314.0.pkl&mstagHits=DSNYHLLMSVQESLER&cycle=1&fixedMods=carbamidomethylation&varMods=) | 18.22 | (R)DSNYHLLMSVQESLER(K) |
|  |  | 11.77 | 11.77 | [71.3](file:///E:\millscripts\viewfeed.pl%3fviewer=viewMaster.jar&side=spectrumWin&spectrumFiles=msdataSM\140307-00027-DP\8729\cpick_in\8729-Pos-aMSMS-30_47.7281.7281.0.pkl&mstagHits=DSNYHLLMSVQESLER&cycle=1&fixedMods=carbamidomethylation&varMods=) | 18.23 | (R)DSNYHLLMSVQESLER(K) |
|  |  | 11.54 | 11.54 | [74.4](file:///E:\millscripts\viewfeed.pl%3fviewer=viewMaster.jar&side=spectrumWin&spectrumFiles=msdataSM\140307-00027-DP\8729\cpick_in\8729-Pos-aMSMS-30_47.7287.7287.0.pkl&mstagHits=DSNYHLLMSVQESLER&cycle=1&fixedMods=carbamidomethylation&varMods=) | 18.25 | (R)DSNYHLLMSVQESLER(K) |
|  |  | 11.47 | 11.47 | [75.1](file:///E:\millscripts\viewfeed.pl%3fviewer=viewMaster.jar&side=spectrumWin&spectrumFiles=msdataSM\140307-00027-DP\8729\cpick_in\8729-Pos-aMSMS-30_47.6074.6082.0.pkl&mstagHits=MVEGFFDR&cycle=1&fixedMods=carbamidomethylation&varMods=) | 14.79 | (K)MVEGFFDR(G) |
|  |  | 10.79 | 10.79 | [66.7](file:///E:\millscripts\viewfeed.pl%3fviewer=viewMaster.jar&side=spectrumWin&spectrumFiles=msdataSM\140307-00027-DP\8729\cpick_in\8729-Pos-aMSMS-30_47.7472.7472.0.pkl&mstagHits=MSILGMTPGFGDK&cycle=1&fixedMods=carbamidomethylation&varMods=) | 18.75 | (-)MSILGMTPGFGDK(T) |
|  |  | 9.24 | 9.24 | [73.1](file:///E:\millscripts\viewfeed.pl%3fviewer=viewMaster.jar&side=spectrumWin&spectrumFiles=msdataSM\140307-00027-DP\8729\cpick_in\8729-Pos-aMSMS-30_47.6104.6104.0.pkl&mstagHits=DIVHSGLAYTMER&cycle=1&fixedMods=carbamidomethylation&varMods=) | 14.88 | (K)DIVHSGLAYTMER(S) |
|  |  |  |  |  |  |  |
| 21 | Annexin-A5 | 21.59 | 10.45 | [96.8](file:///E:\millscripts\viewfeed.pl%3fviewer=viewMaster.jar&side=spectrumWin&spectrumFiles=msdataSM\140611-00095-DP\9733\cpick_in\9733-Pos-aMSMS-30_47.5555.5605.0.pkl&mstagHits=LIVALMKPSR&cycle=1&fixedMods=carbamidomethylation&varMods=) | 14.07 | (K)LIVALMKPSR(L) |
|  |  | 20.14 | 20.14 | [97.5](file:///E:\millscripts\viewfeed.pl%3fviewer=viewMaster.jar&side=spectrumWin&spectrumFiles=msdataSM\140611-00095-DP\9733\cpick_in\9733-Pos-aMSMS-30_47.4963.4978.2.pkl&mstagHits=LYDAYELK&cycle=1&fixedMods=carbamidomethylation&varMods=) | 12.42 | (R)LYDAYELK(H) |
|  |  | 19.50 | 19.50 | [95.6](file:///E:\millscripts\viewfeed.pl%3fviewer=viewMaster.jar&side=spectrumWin&spectrumFiles=msdataSM\140611-00095-DP\9733\cpick_in\9733-Pos-aMSMS-30_47.5841.5918.0.pkl&mstagHits=GTVTDFPGFDER&cycle=1&fixedMods=carbamidomethylation&varMods=) | 14.89 | (R)GTVTDFPGFDER(A) |
|  |  | 19.48 | 19.48 | [85.9](file:///E:\millscripts\viewfeed.pl%3fviewer=viewMaster.jar&side=spectrumWin&spectrumFiles=msdataSM\140611-00095-DP\9733\cpick_in\9733-Pos-aMSMS-30_47.3404.3445.0.pkl&mstagHits=GAGTDDHTLIR&cycle=1&fixedMods=carbamidomethylation&varMods=) | 8.41 | (K)GAGTDDHTLIR(V) |
|  |  | 19.46 | 19.46 | [95.8](file:///E:\millscripts\viewfeed.pl%3fviewer=viewMaster.jar&side=spectrumWin&spectrumFiles=msdataSM\140611-00095-DP\9733\cpick_in\9733-Pos-aMSMS-30_47.6648.6653.0.pkl&mstagHits=MLVVLLQANR&cycle=1&fixedMods=carbamidomethylation&varMods=) | 17.20 | (R)MLVVLLQANR(D) |
|  |  | 19.05 | 12.39 | [96.9](file:///E:\millscripts\viewfeed.pl%3fviewer=viewMaster.jar&side=spectrumWin&spectrumFiles=msdataSM\140611-00095-DP\9733\cpick_in\9733-Pos-aMSMS-30_47.5252.5307.2.pkl&mstagHits=VLTEIIASR&cycle=1&fixedMods=carbamidomethylation&varMods=) | 13.16 | (K)VLTEIIASR(T) |
|  |  | 15.81 | 15.81 | [85.7](file:///E:\millscripts\viewfeed.pl%3fviewer=viewMaster.jar&side=spectrumWin&spectrumFiles=msdataSM\140611-00095-DP\9733\cpick_in\9733-Pos-aMSMS-30_47.6984.7038.2.pkl&mstagHits=SEIDLFNIR&cycle=1&fixedMods=carbamidomethylation&varMods=) | 18.15 | (R)SEIDLFNIR(K) |
|  |  | 14.90 | 14.90 | [72.2](file:///E:\millscripts\viewfeed.pl%3fviewer=viewMaster.jar&side=spectrumWin&spectrumFiles=msdataSM\140611-00095-DP\9733\cpick_in\9733-Pos-aMSMS-30_47.7857.7918.0.pkl&mstagHits=NFATSLYSMIK&cycle=1&fixedMods=carbamidomethylation&varMods=) | 20.38 | (K)NFATSLYSMIK(G) |
|  |  | 14.81 | 10.40 | [88.1](file:///E:\millscripts\viewfeed.pl%3fviewer=viewMaster.jar&side=spectrumWin&spectrumFiles=msdataSM\140611-00095-DP\9733\cpick_in\9733-Pos-aMSMS-30_47.4309.4318.2.pkl&mstagHits=QEISAAFK&cycle=1&fixedMods=carbamidomethylation&varMods=) | 10.70 | (R)QEISAAFK(T) |
|  |  | 14.75 | 14.75 | [72.2](file:///E:\millscripts\viewfeed.pl%3fviewer=viewMaster.jar&side=spectrumWin&spectrumFiles=msdataSM\140611-00095-DP\9733\cpick_in\9733-Pos-aMSMS-30_47.3410.3410.0.pkl&mstagHits=GAGTDDHTLIR&cycle=1&fixedMods=carbamidomethylation&varMods=) | 8.44 | (K)GAGTDDHTLIR(V) |
|  |  | 14.27 | 14.27 | [84.4](file:///E:\millscripts\viewfeed.pl%3fviewer=viewMaster.jar&side=spectrumWin&spectrumFiles=msdataSM\140611-00095-DP\9733\cpick_in\9733-Pos-aMSMS-30_47.4273.4276.0.pkl&mstagHits=QEISAAFK&cycle=1&fixedMods=carbamidomethylation&varMods=) | 10.60 | (R)QEISAAFK(T) |
|  |  | 14.10 | 14.10 | [76](file:///E:\millscripts\viewfeed.pl%3fviewer=viewMaster.jar&side=spectrumWin&spectrumFiles=msdataSM\140611-00095-DP\9733\cpick_in\9733-Pos-aMSMS-30_47.6197.6200.0.pkl&mstagHits=SEIDLFNIRK&cycle=1&fixedMods=carbamidomethylation&varMods=).0 | 15.88 | (R)SEIDLFNIRK(E) |
|  |  | 13.93 | 5.81 | [82.5](file:///E:\millscripts\viewfeed.pl%3fviewer=viewMaster.jar&side=spectrumWin&spectrumFiles=msdataSM\140611-00095-DP\9733\cpick_in\9733-Pos-aMSMS-30_47.5566.5572.0.pkl&mstagHits=LIVALMKPSR&cycle=1&fixedMods=carbamidomethylation&varMods=) | 14.10 | (K)LIVALMKPSR(L) |
|  |  | 13.91 | 13.91 | [85.1](file:///E:\millscripts\viewfeed.pl%3fviewer=viewMaster.jar&side=spectrumWin&spectrumFiles=msdataSM\140611-00095-DP\9733\cpick_in\9733-Pos-aMSMS-30_47.2722.2725.0.pkl&mstagHits=TPEELR&cycle=1&fixedMods=carbamidomethylation&varMods=) | 6.70 | (R)TPEELR(A) |
|  |  | 12.24 | 12.24 | [70.9](file:///E:\millscripts\viewfeed.pl%3fviewer=viewMaster.jar&side=spectrumWin&spectrumFiles=msdataSM\140611-00095-DP\9733\cpick_in\9733-Pos-aMSMS-30_47.7285.7285.0.pkl&mstagHits=YMTISGFQIEETIDR&cycle=1&fixedMods=carbamidomethylation&varMods=) | 18.86 | (K)YMTISGFQIEETIDR(E) |
|  |  | 11.57 | 5.44 | [79.9](file:///E:\millscripts\viewfeed.pl%3fviewer=viewMaster.jar&side=spectrumWin&spectrumFiles=msdataSM\140611-00095-DP\9733\cpick_in\9733-Pos-aMSMS-30_47.6013.6017.0.pkl&mstagHits=LIVALMK&cycle=1&fixedMods=carbamidomethylation&varMods=) | 15.38 | (K)LIVALMK(X) |
|  |  | 11.43 | 0.09 | [83.1](file:///E:\millscripts\viewfeed.pl%3fviewer=viewMaster.jar&side=spectrumWin&spectrumFiles=msdataSM\140611-00095-DP\9733\cpick_in\9733-Pos-aMSMS-30_47.6185.6188.0.pkl&mstagHits=DLLDDLK&cycle=1&fixedMods=carbamidomethylation&varMods=) | 15.83 | (R)DLLDDLK(S) |
|  |  | 8.55 | 2.79 | [65.3](file:///E:\millscripts\viewfeed.pl%3fviewer=viewMaster.jar&side=spectrumWin&spectrumFiles=msdataSM\140611-00095-DP\9733\cpick_in\9733-Pos-aMSMS-30_47.6722.6727.0.pkl&mstagHits=FITIFGTR&cycle=1&fixedMods=carbamidomethylation&varMods=) | 17.47 | (K)FITIFGTR(S) |
|  |  |  |  |  |  |  |
| 22 | Heat shock 70kDa | 24.95 | 19.42 | [100](file:///E:\millscripts\viewfeed.pl%3fviewer=viewMaster.jar&side=spectrumWin&spectrumFiles=msdataSM\140611-00095-DP\10544\cpick_in\10544-Pos-aMSMS-30_47.8125.8192.0.pkl&mstagHits=LYSPSQIGAFVLMK&cycle=1&fixedMods=carbamidomethylation&varMods=) | 20.63 | (K)LYSPSQIGAFVLMK(M) |
|  | protein 9 | 21.57 | 21.57 | [98.1](file:///E:\millscripts\viewfeed.pl%3fviewer=viewMaster.jar&side=spectrumWin&spectrumFiles=msdataSM\140611-00095-DP\10544\cpick_in\10544-Pos-aMSMS-30_47.7090.7125.0.pkl&mstagHits=SDIGEVILVGGMTR&cycle=1&fixedMods=carbamidomethylation&varMods=) | 17.76 | (K)SDIGEVILVGGMTR(M) |
|  |  | 20.64 | 14.04 | [94.6](file:///E:\millscripts\viewfeed.pl%3fviewer=viewMaster.jar&side=spectrumWin&spectrumFiles=msdataSM\140611-00095-DP\10544\cpick_in\10544-Pos-aMSMS-30_47.6840.6893.0.pkl&mstagHits=VEAVNMAEGIIHDTETK&cycle=1&fixedMods=carbamidomethylation&varMods=) | 17.11 | (R)VEAVNMAEGIIHDTETK(M) |
|  |  | 18.04 | 18.04 | [81.7](file:///E:\millscripts\viewfeed.pl%3fviewer=viewMaster.jar&side=spectrumWin&spectrumFiles=msdataSM\140611-00095-DP\10544\cpick_in\10544-Pos-aMSMS-30_47.7832.7895.2.pkl&mstagHits=AQFEGIVTDLIR&cycle=1&fixedMods=carbamidomethylation&varMods=) | 19.77 | (R)AQFEGIVTDLIR(R) |
|  |  | 17.28 | 17.28 | [87.8](file:///E:\millscripts\viewfeed.pl%3fviewer=viewMaster.jar&side=spectrumWin&spectrumFiles=msdataSM\140611-00095-DP\10544\cpick_in\10544-Pos-aMSMS-30_47.6453.6494.0.pkl&mstagHits=EQQIVIQSSGGLSKDDIENMVK&cycle=1&fixedMods=carbamidomethylation&varMods=) | 15.90 | (R)EQQIVIQSSGGLSKDDIENMVK(N) |
|  |  | 16.72 | 16.72 | [84](file:///E:\millscripts\viewfeed.pl%3fviewer=viewMaster.jar&side=spectrumWin&spectrumFiles=msdataSM\140611-00095-DP\10544\cpick_in\10544-Pos-aMSMS-30_47.8220.8401.0.pkl&mstagHits=LLGQFTLIGIPPAPR&cycle=1&fixedMods=carbamidomethylation&varMods=).0 | 20.92 | (K)LLGQFTLIGIPPAPR(G) |
|  |  | 16.09 | 9.98 | [87.5](file:///E:\millscripts\viewfeed.pl%3fviewer=viewMaster.jar&side=spectrumWin&spectrumFiles=msdataSM\140611-00095-DP\10544\cpick_in\10544-Pos-aMSMS-30_47.6679.6726.0.pkl&mstagHits=VINEPTAAALAYGLDK&cycle=1&fixedMods=carbamidomethylation&varMods=) | 16.60 | (R)VINEPTAAALAYGLDK(S) |
|  |  | 14.72 | 14.72 | [72.8](file:///E:\millscripts\viewfeed.pl%3fviewer=viewMaster.jar&side=spectrumWin&spectrumFiles=msdataSM\140611-00095-DP\10544\cpick_in\10544-Pos-aMSMS-30_47.8418.8571.0.pkl&mstagHits=LLGQFTLIGIPPAPR&cycle=1&fixedMods=carbamidomethylation&varMods=) | 21.43 | (K)LLGQFTLIGIPPAPR(G) |
|  |  | 14.45 | 14.45 | [71.5](file:///E:\millscripts\viewfeed.pl%3fviewer=viewMaster.jar&side=spectrumWin&spectrumFiles=msdataSM\140611-00095-DP\10544\cpick_in\10544-Pos-aMSMS-30_47.6102.6105.0.pkl&mstagHits=VQQTVQDLFGR&cycle=1&fixedMods=carbamidomethylation&varMods=) | 14.95 | (K)VQQTVQDLFGR(A) |
|  |  | 14.40 | 14.40 | [77.2](file:///E:\millscripts\viewfeed.pl%3fviewer=viewMaster.jar&side=spectrumWin&spectrumFiles=msdataSM\140611-00095-DP\10544\cpick_in\10544-Pos-aMSMS-30_47.6641.6650.0.pkl&mstagHits=NAVITVPAYFNDSQR&cycle=1&fixedMods=carbamidomethylation&varMods=) | 16.48 | (K)NAVITVPAYFNDSQR(Q) |
|  |  | 14.34 | 14.34 | [73](file:///E:\millscripts\viewfeed.pl%3fviewer=viewMaster.jar&side=spectrumWin&spectrumFiles=msdataSM\140611-00095-DP\10544\cpick_in\10544-Pos-aMSMS-30_47.6870.6870.0.pkl&mstagHits=VEAVNMAEGIIHDTETK&cycle=1&fixedMods=carbamidomethylation&varMods=).0 | 17.24 | (R)VEAVNMAEGIIHDTETK(M) |
|  |  | 14.29 | 10.62 | [79.8](file:///E:\millscripts\viewfeed.pl%3fviewer=viewMaster.jar&side=spectrumWin&spectrumFiles=msdataSM\140611-00095-DP\10544\cpick_in\10544-Pos-aMSMS-30_47.6844.6844.0.pkl&mstagHits=VEAVNMAEGIIHDTETK&cycle=1&fixedMods=carbamidomethylation&varMods=) | 17.12 | (R)VEAVNMAEGIIHDTETK(M) |
|  |  | 13.95 | 13.95 | [84.6](file:///E:\millscripts\viewfeed.pl%3fviewer=viewMaster.jar&side=spectrumWin&spectrumFiles=msdataSM\140611-00095-DP\10544\cpick_in\10544-Pos-aMSMS-30_47.8410.8520.0.pkl&mstagHits=LLGQFTLIGIPPAPR&cycle=1&fixedMods=carbamidomethylation&varMods=) | 21.40 | (K)LLGQFTLIGIPPAPR(G) |
|  |  | 13.73 | 13.73 | [83.4](file:///E:\millscripts\viewfeed.pl%3fviewer=viewMaster.jar&side=spectrumWin&spectrumFiles=msdataSM\140611-00095-DP\10544\cpick_in\10544-Pos-aMSMS-30_47.6194.6232.0.pkl&mstagHits=DAGQISGLNVLR&cycle=1&fixedMods=carbamidomethylation&varMods=) | 15.22 | (K)DAGQISGLNVLR(V) |
|  |  | 12.15 | 12.15 | [74.2](file:///E:\millscripts\viewfeed.pl%3fviewer=viewMaster.jar&side=spectrumWin&spectrumFiles=msdataSM\140611-00095-DP\10544\cpick_in\10544-Pos-aMSMS-30_47.8199.8199.0.pkl&mstagHits=LLGQFTLIGIPPAPR&cycle=1&fixedMods=carbamidomethylation&varMods=) | 20.85 | (K)LLGQFTLIGIPPAPR(G) |
|  |  | 11.21 | 11.21 | [66.6](file:///E:\millscripts\viewfeed.pl%3fviewer=viewMaster.jar&side=spectrumWin&spectrumFiles=msdataSM\140611-00095-DP\10544\cpick_in\10544-Pos-aMSMS-30_47.6718.6718.0.pkl&mstagHits=ERVEAVNMAEGIIHDTETK&cycle=1&fixedMods=carbamidomethylation&varMods=) | 16.75 | (K)ERVEAVNMAEGIIHDTETK  (M) |
|  |  | 10.35 | 10.35 | [73.4](file:///E:\millscripts\viewfeed.pl%3fviewer=viewMaster.jar&side=spectrumWin&spectrumFiles=msdataSM\140611-00095-DP\10544\cpick_in\10544-Pos-aMSMS-30_47.8198.8282.0.pkl&mstagHits=LLGQFTLIGIPPAPR&cycle=1&fixedMods=carbamidomethylation&varMods=) | 20.85 | (K)LLGQFTLIGIPPAPR(G) |
|  |  | 9.59 | 9.59 | [65.7](file:///E:\millscripts\viewfeed.pl%3fviewer=viewMaster.jar&side=spectrumWin&spectrumFiles=msdataSM\140611-00095-DP\10544\cpick_in\10544-Pos-aMSMS-30_47.8128.8128.0.pkl&mstagHits=LYSPSQIGAFVLMK&cycle=1&fixedMods=carbamidomethylation&varMods=) | 20.64 | (K)LYSPSQIGAFVLMK(M) |
|  |  | 8.76 | 8.76 | [60.5](file:///E:\millscripts\viewfeed.pl%3fviewer=viewMaster.jar&side=spectrumWin&spectrumFiles=msdataSM\140611-00095-DP\10544\cpick_in\10544-Pos-aMSMS-30_47.5373.5373.0.pkl&mstagHits=SQVFSTAADGQTQVEIK&cycle=1&fixedMods=carbamidomethylation&varMods=) | 13.18 | (K)SQVFSTAADGQTQVEIK(V) |
|  |  | 7.89 | 7.89 | [67.7](file:///E:\millscripts\viewfeed.pl%3fviewer=viewMaster.jar&side=spectrumWin&spectrumFiles=msdataSM\140611-00095-DP\10544\cpick_in\10544-Pos-aMSMS-30_47.6197.6197.0.pkl&mstagHits=DAGQISGLNVLR&cycle=1&fixedMods=carbamidomethylation&varMods=) | 15.25 | (K)DAGQISGLNVLR(V) |
|  |  | 7.27 | 7.27 | [61.5](file:///E:\millscripts\viewfeed.pl%3fviewer=viewMaster.jar&side=spectrumWin&spectrumFiles=msdataSM\140611-00095-DP\10544\cpick_in\10544-Pos-aMSMS-30_47.6632.6632.0.pkl&mstagHits=NAVITVPAYFNDSQR&cycle=1&fixedMods=carbamidomethylation&varMods=) | 16.45 | (K)NAVITVPAYFNDSQR(Q) |
|  |  |  |  |  |  |  |
| 23 | 26S proteasome non- | 15.48 | 15.48 | [78.4](file:///E:\millscripts\viewfeed.pl%3fviewer=viewMaster.jar&side=spectrumWin&spectrumFiles=msdataSM\140611-00095-DP\15749\cpick_in\15749-Pos-aMSMS-30_47.7912.7923.0.pkl&mstagHits=VVVHPLVLLSVVDHFNR&cycle=1&fixedMods=carbamidomethylation&varMods=) | 20.59 | (K)VVVHPLVLLSVVDHFNR(I) |
|  | ATPase regulatory | 13.36 | 13.36 | [60.6](file:///E:\millscripts\viewfeed.pl%3fviewer=viewMaster.jar&side=spectrumWin&spectrumFiles=msdataSM\140611-00095-DP\15749\cpick_in\15749-Pos-aMSMS-30_47.6905.6922.0.pkl&mstagHits=YCPNSVLVIIDVKPK&cycle=1&fixedMods=carbamidomethylation&varMods=) | 17.82 | (R)YCPNSVLVIIDVKPK(D) + C |
|  | subunit 7 | 12.54 | 8.77 | [80.4](file:///E:\millscripts\viewfeed.pl%3fviewer=viewMaster.jar&side=spectrumWin&spectrumFiles=msdataSM\140611-00095-DP\15749\cpick_in\15749-Pos-aMSMS-30_47.6581.6584.0.pkl&mstagHits=VVGVLLGSWQK&cycle=1&fixedMods=carbamidomethylation&varMods=) | 16.96 | (R)VVGVLLGSWQK(K) |
|  |  | 9.90 | 6.05 | [70.7](file:///E:\millscripts\viewfeed.pl%3fviewer=viewMaster.jar&side=spectrumWin&spectrumFiles=msdataSM\140611-00095-DP\15749\cpick_in\15749-Pos-aMSMS-30_47.5974.6020.0.pkl&mstagHits=NDIAINELMK&cycle=1&fixedMods=carbamidomethylation&varMods=) | 15.31 | (K)NDIAINELMK(R) |
|  |  | 9.78 | 9.78 | [62.7](file:///E:\millscripts\viewfeed.pl%3fviewer=viewMaster.jar&side=spectrumWin&spectrumFiles=msdataSM\140611-00095-DP\15749\cpick_in\15749-Pos-aMSMS-30_47.7877.7877.0.pkl&mstagHits=VVVHPLVLLSVVDHFNR&cycle=1&fixedMods=carbamidomethylation&varMods=) | 20.50 | (K)VVVHPLVLLSVVDHFNR(I) |
|  |  | 8.29 | 8.29 | [61.6](file:///E:\millscripts\viewfeed.pl%3fviewer=viewMaster.jar&side=spectrumWin&spectrumFiles=msdataSM\140611-00095-DP\15749\cpick_in\15749-Pos-aMSMS-30_47.4587.4587.0.pkl&mstagHits=IVGWYHTGPK&cycle=1&fixedMods=carbamidomethylation&varMods=) | 11.53 | (R)IVGWYHTGPK(L) |
|  |  |  |  |  |  |  |
| 24 | Transaldolase 1 | 14.22 | 14.22 | [84.5](file:///E:\millscripts\viewfeed.pl%3fviewer=viewMaster.jar&side=spectrumWin&spectrumFiles=msdataSM\140611-00095-DP\16185\cpick_in\16185-Pos-aMSMS-30_47.6433.6433.0.pkl&mstagHits=LLGELLQDNAK&cycle=1&fixedMods=carbamidomethylation&varMods=) | 15.64 | (K)LLGELLQDNAK(L) |
|  |  | 10.89 | 5.30 | [79.6](file:///E:\millscripts\viewfeed.pl%3fviewer=viewMaster.jar&side=spectrumWin&spectrumFiles=msdataSM\140611-00095-DP\16185\cpick_in\16185-Pos-aMSMS-30_47.5334.5342.0.pkl&mstagHits=LVPVLSAK&cycle=1&fixedMods=carbamidomethylation&varMods=) | 13.06 | (K)LVPVLSAK(A) |
|  |  |  |  |  |  |  |
| 25 | Proteasome activator | 10.77 | 10.77 | [64.6](file:///E:\millscripts\viewfeed.pl%3fviewer=viewMaster.jar&side=spectrumWin&spectrumFiles=msdataSM\140611-00095-DP\17235\cpick_in\17235-Pos-aMSMS-30_47.9458.9458.0.pkl&mstagHits=NAYAVLYDIILK&cycle=1&fixedMods=carbamidomethylation&varMods=) | 22.99 | (R)NAYAVLYDIILK(N) |
|  | complex subunit 1 | 9.25 | 9.25 | [61.7](file:///E:\millscripts\viewfeed.pl%3fviewer=viewMaster.jar&side=spectrumWin&spectrumFiles=msdataSM\140611-00095-DP\17235\cpick_in\17235-Pos-aMSMS-30_47.7272.7272.0.pkl&mstagHits=TENLLGSYFPK&cycle=1&fixedMods=carbamidomethylation&varMods=) | 17.68 | (K)TENLLGSYFPK(K) |
|  |  |  |  |  |  |  |
| 26 | ATP-citrate synthase, | 19.98 | 19.98 | [96.8](file:///E:\millscripts\viewfeed.pl%3fviewer=viewMaster.jar&side=spectrumWin&spectrumFiles=msdataSM\140611-00095-DP\18720\cpick_in\18720-Pos-aMSMS-30_47.6795.6835.0.pkl&mstagHits=DGVYVLDLAAK&cycle=1&fixedMods=carbamidomethylation&varMods=) | 16.93 | (K)DGVYVLDLAAK(V) |
|  | isoform X1 | 17.42 | 17.42 | [78.8](file:///E:\millscripts\viewfeed.pl%3fviewer=viewMaster.jar&side=spectrumWin&spectrumFiles=msdataSM\140611-00095-DP\18720\cpick_in\18720-Pos-aMSMS-30_47.4312.4333.0.pkl&mstagHits=VDATADYICK&cycle=1&fixedMods=carbamidomethylation&varMods=) | 10.54 | (K)VDATADYICK(V) + C |
|  |  | 17.26 | 11.65 | [80.1](file:///E:\millscripts\viewfeed.pl%3fviewer=viewMaster.jar&side=spectrumWin&spectrumFiles=msdataSM\140611-00095-DP\18720\cpick_in\18720-Pos-aMSMS-30_47.4619.4622.0.pkl&mstagHits=FICTTSAIQNR&cycle=1&fixedMods=carbamidomethylation&varMods=) | 11.35 | (K)FICTTSAIQNR(F) + C |
|  |  | 14.31 | 14.31 | [79.9](file:///E:\millscripts\viewfeed.pl%3fviewer=viewMaster.jar&side=spectrumWin&spectrumFiles=msdataSM\140611-00095-DP\18720\cpick_in\18720-Pos-aMSMS-30_47.5247.5250.0.pkl&mstagHits=LTLLNPK&cycle=1&fixedMods=carbamidomethylation&varMods=) | 12.93 | (K)LTLLNPK(G) |
|  |  | 14.06 | 14.06 | [75.1](file:///E:\millscripts\viewfeed.pl%3fviewer=viewMaster.jar&side=spectrumWin&spectrumFiles=msdataSM\140611-00095-DP\18720\cpick_in\18720-Pos-aMSMS-30_47.7807.7891.0.pkl&mstagHits=LGLVGVNLTLDGVK&cycle=1&fixedMods=carbamidomethylation&varMods=) | 19.40 | (K)LGLVGVNLTLDGVK(S) |
|  |  | 13.46 | 13.46 | [64.2](file:///E:\millscripts\viewfeed.pl%3fviewer=viewMaster.jar&side=spectrumWin&spectrumFiles=msdataSM\140611-00095-DP\18720\cpick_in\18720-Pos-aMSMS-30_47.6604.6604.0.pkl&mstagHits=EAYPEEAYIADLDAK&cycle=1&fixedMods=carbamidomethylation&varMods=) | 16.40 | (R)EAYPEEAYIADLDAK(S) |
|  |  | 13.00 | 5.37 | [84.1](file:///E:\millscripts\viewfeed.pl%3fviewer=viewMaster.jar&side=spectrumWin&spectrumFiles=msdataSM\140611-00095-DP\18720\cpick_in\18720-Pos-aMSMS-30_47.4677.4680.0.pkl&mstagHits=LLVGVDEK&cycle=1&fixedMods=carbamidomethylation&varMods=) | 11.56 | (K)LLVGVDEK(L) |
|  |  | 12.15 | 12.15 | [70.6](file:///E:\millscripts\viewfeed.pl%3fviewer=viewMaster.jar&side=spectrumWin&spectrumFiles=msdataSM\140611-00095-DP\18720\cpick_in\18720-Pos-aMSMS-30_47.3875.3875.0.pkl&mstagHits=GGPNYQEGLR&cycle=1&fixedMods=carbamidomethylation&varMods=) | 9.54 | (R)GGPNYQEGLR(V) |
|  |  | 11.97 | 7.27 | [77.1](file:///E:\millscripts\viewfeed.pl%3fviewer=viewMaster.jar&side=spectrumWin&spectrumFiles=msdataSM\140611-00095-DP\18720\cpick_in\18720-Pos-aMSMS-30_47.6649.6652.0.pkl&mstagHits=TILSLMTR&cycle=1&fixedMods=carbamidomethylation&varMods=) | 16.54 | (K)TILSLMTR(E) |
|  |  | 9.17 | 9.17 | [60.6](file:///E:\millscripts\viewfeed.pl%3fviewer=viewMaster.jar&side=spectrumWin&spectrumFiles=msdataSM\140611-00095-DP\18720\cpick_in\18720-Pos-aMSMS-30_47.6798.6798.0.pkl&mstagHits=DGVYVLDLAAK&cycle=1&fixedMods=carbamidomethylation&varMods=) | 16.95 | (K)DGVYVLDLAAK(V) |
|  |  |  |  |  |  |  |
| 27 | Biliverdin IX alpha | 15.00 | 5.55 | [80.5](file:///E:\millscripts\viewfeed.pl%3fviewer=viewMaster.jar&side=spectrumWin&spectrumFiles=msdataSM\140611-00095-DP\18732\cpick_in\18732-Pos-aMSMS-30_47.5934.5943.0.pkl&mstagHits=FGVVVVGVGR&cycle=1&fixedMods=carbamidomethylation&varMods=) | 15.20 | (K)FGVVVVGVGR(A) |
|  | reductase | 14.22 | 14.22 | [71.3](file:///E:\millscripts\viewfeed.pl%3fviewer=viewMaster.jar&side=spectrumWin&spectrumFiles=msdataSM\140611-00095-DP\18732\cpick_in\18732-Pos-aMSMS-30_47.7102.7199.0.pkl&mstagHits=FGFPAFSGISR&cycle=1&fixedMods=carbamidomethylation&varMods=) | 18.79 | (R)FGFPAFSGISR(L) |
|  |  | 9.92 | 9.92 | [73.8](file:///E:\millscripts\viewfeed.pl%3fviewer=viewMaster.jar&side=spectrumWin&spectrumFiles=msdataSM\140611-00095-DP\18732\cpick_in\18732-Pos-aMSMS-30_47.4721.4725.0.pkl&mstagHits=LLGQFSEK&cycle=1&fixedMods=carbamidomethylation&varMods=) | 11.87 | (K)LLGQFSEK(E) |
|  |  | 8.27 | 8.27 | [65.4](file:///E:\millscripts\viewfeed.pl%3fviewer=viewMaster.jar&side=spectrumWin&spectrumFiles=msdataSM\140611-00095-DP\18732\cpick_in\18732-Pos-aMSMS-30_47.5937.5937.0.pkl&mstagHits=FGVVVVGVGR&cycle=1&fixedMods=carbamidomethylation&varMods=) | 15.21 | (K)FGVVVVGVGR(A) |
|  |  |  |  |  |  |  |
| 28 | Electron-transfer- | 15.16 | 7.27 | [83.6](file:///E:\millscripts\viewfeed.pl%3fviewer=viewMaster.jar&side=spectrumWin&spectrumFiles=msdataSM\140611-00095-DP\19672\cpick_in\19672-Pos-aMSMS-30_47.5261.5264.0.pkl&mstagHits=EIDGGLETLR&cycle=1&fixedMods=carbamidomethylation&varMods=) | 12.92 | (R)EIDGGLETLR(L) |
|  | flavoprotein, beta | 13.41 | 7.26 | [88.2](file:///E:\millscripts\viewfeed.pl%3fviewer=viewMaster.jar&side=spectrumWin&spectrumFiles=msdataSM\140611-00095-DP\19672\cpick_in\19672-Pos-aMSMS-30_47.5493.5502.0.pkl&mstagHits=LSVISVEDPPQR&cycle=1&fixedMods=carbamidomethylation&varMods=) | 13.53 | (K)LSVISVEDPPQR(T) |
|  | polypeptide | 12.18 | 12.18 | [70.9](file:///E:\millscripts\viewfeed.pl%3fviewer=viewMaster.jar&side=spectrumWin&spectrumFiles=msdataSM\140611-00095-DP\19672\cpick_in\19672-Pos-aMSMS-30_47.6699.6699.0.pkl&mstagHits=VDLVLLGK&cycle=1&fixedMods=carbamidomethylation&varMods=) | 16.43 | (K)VDLVLLGK(Q) |
|  |  | 10.06 | 10.06 | [64.4](file:///E:\millscripts\viewfeed.pl%3fviewer=viewMaster.jar&side=spectrumWin&spectrumFiles=msdataSM\140611-00095-DP\19672\cpick_in\19672-Pos-aMSMS-30_47.4057.4061.0.pkl&mstagHits=VIDYAVK&cycle=1&fixedMods=carbamidomethylation&varMods=) | 9.97 | (R)VIDYAVK(I) |
|  |  |  |  |  |  |  |
| 29 | Actin-related protein 2/3 | 14.86 | 8.16 | [83.7](file:///E:\millscripts\viewfeed.pl%3fviewer=viewMaster.jar&side=spectrumWin&spectrumFiles=msdataSM\140611-00095-DP\19699\cpick_in\19699-Pos-aMSMS-30_47.7064.7067.0.pkl&mstagHits=AENFFILR&cycle=1&fixedMods=carbamidomethylation&varMods=) | 17.80 | (R)AENFFILR(R) |
|  | complex subunit 4 | 13.47 | 7.51 | [80.7](file:///E:\millscripts\viewfeed.pl%3fviewer=viewMaster.jar&side=spectrumWin&spectrumFiles=msdataSM\140611-00095-DP\19699\cpick_in\19699-Pos-aMSMS-30_47.6647.6650.0.pkl&mstagHits=ELLLQPVTISR&cycle=1&fixedMods=carbamidomethylation&varMods=) | 16.82 | (K)ELLLQPVTISR(N) |
|  |  | 11.79 | 11.79 | [83.6](file:///E:\millscripts\viewfeed.pl%3fviewer=viewMaster.jar&side=spectrumWin&spectrumFiles=msdataSM\140611-00095-DP\19699\cpick_in\19699-Pos-aMSMS-30_47.5830.5833.0.pkl&mstagHits=IVAEEFLK&cycle=1&fixedMods=carbamidomethylation&varMods=) | 14.72 | (R)IVAEEFLK(N) |
|  |  | 9.63 | 3.76 | [74.7](file:///E:\millscripts\viewfeed.pl%3fviewer=viewMaster.jar&side=spectrumWin&spectrumFiles=msdataSM\140611-00095-DP\19699\cpick_in\19699-Pos-aMSMS-30_47.5268.5272.0.pkl&mstagHits=VLIEGSINSVR&cycle=1&fixedMods=carbamidomethylation&varMods=) | 13.29 | (K)VLIEGSINSVR(V) |
|  |  |  |  |  |  |  |
| 30 | Mitogen-activated | 12.38 | 12.38 | [71.5](file:///E:\millscripts\viewfeed.pl%3fviewer=viewMaster.jar&side=spectrumWin&spectrumFiles=msdataSM\140611-00095-DP\19727\cpick_in\19727-Pos-aMSMS-30_47.4598.4598.0.pkl&mstagHits=APEIMLNSK&cycle=1&fixedMods=carbamidomethylation&varMods=) | 11.53 | (R)APEIMLNSK(G) |
|  | protein kinase 1 | 11.61 | 11.61 | [67.3](file:///E:\millscripts\viewfeed.pl%3fviewer=viewMaster.jar&side=spectrumWin&spectrumFiles=msdataSM\140611-00095-DP\19727\cpick_in\19727-Pos-aMSMS-30_47.4733.4736.0.pkl&mstagHits=GQVFDVGPR&cycle=1&fixedMods=carbamidomethylation&varMods=) | 11.93 | (R)GQVFDVGPR(Y) |
|  |  | 11.31 | 11.31 | [66.2](file:///E:\millscripts\viewfeed.pl%3fviewer=viewMaster.jar&side=spectrumWin&spectrumFiles=msdataSM\140611-00095-DP\19727\cpick_in\19727-Pos-aMSMS-30_47.5657.5671.0.pkl&mstagHits=NYLLSLPHK&cycle=1&fixedMods=carbamidomethylation&varMods=) | 14.37 | (R)NYLLSLPHK(N) |
|  |  | 10.28 | 5.29 | [74.3](file:///E:\millscripts\viewfeed.pl%3fviewer=viewMaster.jar&side=spectrumWin&spectrumFiles=msdataSM\140611-00095-DP\19727\cpick_in\19727-Pos-aMSMS-30_47.5325.5328.0.pkl&mstagHits=ALDLLDK&cycle=1&fixedMods=carbamidomethylation&varMods=) | 13.46 | (K)ALDLLDK(M) |
|  |  | 8.52 | 2.88 | [60.7](file:///E:\millscripts\viewfeed.pl%3fviewer=viewMaster.jar&side=spectrumWin&spectrumFiles=msdataSM\140611-00095-DP\19727\cpick_in\19727-Pos-aMSMS-30_47.5656.5656.0.pkl&mstagHits=ELIFEETAR&cycle=1&fixedMods=carbamidomethylation&varMods=) | 14.37 | (K)ELIFEETAR(F) |
|  |  | 7.94 | 7.94 | [61.1](file:///E:\millscripts\viewfeed.pl%3fviewer=viewMaster.jar&side=spectrumWin&spectrumFiles=msdataSM\140611-00095-DP\19727\cpick_in\19727-Pos-aMSMS-30_47.6789.6792.0.pkl&mstagHits=DLKPSNLLLNTTCDLK&cycle=1&fixedMods=carbamidomethylation&varMods=) | 17.31 | (R)DLKPSNLLLNTTCDLK(I) + C |
|  |  |  |  |  |  |  |

* **C** represents carbamidomethylation modification on peptide sequence.
