# Supplementary material for: Comparative proteomics reveals that YK51, a 4-Hydroxypandurantin-A analogue, downregulates the expression of proteins associated with dengue virus infection
Source: PeerJ. 2018 Jan 30;5:e3939. doi: 10.7717/peerj.3939 (PMC5796277; doi:10.7717/peerj.3939)
Supplement: Table S3 [file peerj-06-3939-s003.docx]

**Supplementary Table S3:** List of identified protein spots in Table 2 and their functional categorization.

| **Protein functions** | **Spot ID** | **Protein Name (Gene Symbols)** | **Swiss-Prot Accession** |
| --- | --- | --- | --- |
| Protein Biosyntheis 34% | 4 | Eukaryotic initiation factor 4A-I (eIF4A-1) | P60842 |
|  | 7 | Alanine aminotransferase 2 (ALAT2) | Q8TD30 |
|  | 9 | leucine aminopeptidase 3 (AMPL) | P28838 |
|  | 12 | Protein disulfide-isomerase A6 (PDIA6) | Q15084 |
|  | 13 | Elongation factor 2 (EF-2) | P13639 |
|  | 14 | Protein disulfide-isomerase A3 (PDIA3) | P30101 |
|  | 17 | T-complex protein 1 subunit alpha (TCP1A) | P17987 |
|  | 18 | Elongation factor Tu (EF-Tu) | P49411 |
|  | 19 | Protein disulfide-isomerase A6 (PDIA6) | Q15084 |
|  | 20 | Glutamate dehydrogenase 1 (GDH 1) | P00367 |
| Protein ubiquitination 10% | 10 | 26S protease regulatory subunit 8 (PRS8) | P62195 |
|  | 23 | 26S proteasome non-ATPase regulatory subunit 7 (PSMD7) | P51665 |
|  | 25 | Proteasome activator complex subunit 1(PSME1) | Q06323 |
| Transcription 7% | 3 | Interleukin enhancer-binding factor 2 (ILF2) | Q12905 |
|  | 5 | Proliferation-associated protein 2G4 (PA2G4) | Q9UQ80 |
| Carbon metabolism 7% | 11 | Glucose-6-phosphate 1-dehydrogenase (G6PDH) | P11413 |
|  | 24 | Transaldolase, 1 (TALDO1) | P37837 |
| Cell cycle progression 7% | 16 | Tubulin alpha-1B chain (TBA1B) | P68363 |
|  | 22 | Heat shock 70kDa protein 9 (HSPA9) | P38646 |
| ATP synthesis 7% | 8 | ATP synthase subunit beta (ATPB) | P06576 |
|  | 15 | ATP synthase subunit beta (ATPB) | P06576 |
| Retinol metabolism 7% | 1 | Retinal dehydrogenase 1 (AL1A1) | P00352 |
|  | 2 | Retinal dehydrogenase 1 (AL1A1) | P00352 |
| Respiratory electron transport 3% | 28 | Electron-transfer-flavoprotein, beta polypeptide (EFTB) | P38117 |
| MAPK signaling 3% | 30 | Mitogen-activated protein kinase 1 (MAPK 1) | P28482 |
| **Supplementary Table S3:** Continued | | | |
| Anticoagulation 3% | 21 | Annexin A5 (ANXA5) | P08758 |
| Purine biosynthesis 3% | 6 | Inosinicase (PUR9) | P31939 |
| Heme metabolism 3% | 27 | biliverdin IX alpha reductase ( BVR A) | P53004 |
| Intracellular transport 3% | 29 | Actin-related protein 2/3 complex subunit 4 (p20-ARC) | P59998 |
| Lipid metabolism 3% | 26 | ATP-citrate synthase, isoform X1 (ACL) | P53396 |
